# C-Reactive Protein Apheresis as Anti-inflammatory Therapy in Acute Myocardial Infarction: Results of the CAMI-1 Study

**DOI:** 10.3389/fcvm.2021.591714

**Published:** 2021-03-10

**Authors:** Wolfgang Ries, Jan Torzewski, Franz Heigl, Christian Pfluecke, Sebastian Kelle, Harald Darius, Hueseyin Ince, Steffen Mitzner, Peter Nordbeck, Christian Butter, Horst Skarabis, Ahmed Sheriff, Christoph D. Garlichs

**Affiliations:** ^1^Medical Clinic, Diakonissenhospital Flensburg, Flensburg, Germany; ^2^Cardiovascular Center Oberallgäu-Kempten, Kempten, Germany; ^3^Medical Care Center Kempten-Allgäu, Kempten, Germany; ^4^Department for Internal Medicine/Cardiology, Heart Center Dresden, Dresden, Germany; ^5^Department of Internal Medicine/Cardiology, German Heart Center Berlin, Berlin, Germany; ^6^Department of Internal Medicine/Cardiology, Charité University Medicine Berlin, Campus Virchow, Berlin, Germany; ^7^Deutsches Zentrum für Herz-Kreislauf-Forschung (German Centre for Cardiovascular Research), Partner Site Berlin, Berlin, Germany; ^8^Clinic for Cardiology, Angiology, Nephrology, Intensive Care Medicine, Vivantes Clinic Neukölln, Berlin, Germany; ^9^Divisions of Cardiology and Nephrology, Department of Internal Medicine, University Medicine Rostock, Rostock, Germany; ^10^Medical Clinic (Cardiology), University Clinic Würzburg, Würzburg, Germany; ^11^Immanuel Clinic Bernau, Heart Center Brandenburg, Bernau, Germany; ^12^Statistical Consultant, Groß-Oesingen, Germany; ^13^Department of Gastroenterology/Infectiology/Rheumatology, Charité University Medicine Berlin, Berlin, Germany

**Keywords:** apheresis, myocardial infarction, CRP, immunoadsorption, inflammation

## Abstract

**Background:** C-reactive protein (CRP) is a well-known marker of inflammation. It is less known that CRP mediates tissue damage in acute myocardial infarction (AMI) thus potentially worsening prognosis. A newly developed specific CRP adsorber allows efficient lowering of CRP levels and may improve survival.

**Objectives:** Aim of this multi-center, controlled, non-randomized first-in-man *CRP apheresis in Acute Myocardial Infarction study (CAMI-1)* was to investigate the relationship between CRP levels (CRP gradient), myocardial infarct size and function as well as safety and efficacy of CRP apheresis in the setting of acute ST-segment Elevation Myocardial Infarction (STEMI) in humans.

**Methods:** Eighty-three patients (45 apheresis, 38 controls) were recruited. CRP apheresis was performed 24 ± 12, 48 ± 12, and optionally 72 ± 12 h after onset of symptoms. First aphereses were performed at a median CRP concentration of 23.0 mg/L (range 9–279). In each apheresis session, 5,900 ± 400 mL plasma was processed *via* peripheral venous access. Primary study endpoint was a reduction in myocardial infarct size after STEMI as determined by cardiovascular magnetic resonance (CMR).

**Results:** In controls, the CRP concentration significantly correlated with infarct size (*p* = 0.002) and decreased myocardial function (*p* ≤ 0.001). The CRP concentration in apheresis patients did not correlate with infarct size (*p* = 0.66) or left ventricular (LV) function (*p* = 0.79) and global strains and therefore significantly differed from controls (*p* = 0.03 and *p* = 0.002). Three major adverse cardiac events occurred in the control group after 12 months, none occurred in the apheresis group. Mean CRP depletion achieved over all apheresis procedures was 53.0 ± 15.1%. Apheresis sessions were well-tolerated. Reduced infarct size in the apheresis group compared to the control group (primary endpoint) was not achieved according to the original statistical analysis plan. Taking into account the individual CRP levels, however, revealed significant results. Modifications of the analysis plan were introduced in order to recruit a sufficient number of patients.

**Conclusions:** This pilot study in humans reveals a correlation between CRP concentration and myocardial infarct size. CRP concentrations in STEMI can effectively be reduced by CRP apheresis without relevant side effects. CRP apheresis has the potential to interfere with deleterious aspects of STEMI. By lowering CRP levels, it resulted in the loss of correlation of CRP concentrations with myocardial infarct sizes as well as LV function. These results encourage a larger, randomized clinical trial.

**Clinical Trial Registration:** https://www.drks.de/drks_web/navigate.do?navigationId=trial.HTML&TRIAL_ID=DRKS00008988, DRKS00008988.

## Introduction

Inflammation plays a central role in the pathophysiology of many diseases. C-reactive protein (CRP) is a well-established sensitive *marker* of inflammation. Regardless of that, a growing body of data has identified CRP as a direct *mediator* of inflammation and immune response (1). This less known role of CRP is based on two immunological functions: (a) activation of the classical complement pathway *via* C1q binding and (b) binding to immunoglobulin Fcγ receptors after opsonisation of biological particles (2–5). Different isoforms of CRP, e.g., monomeric CRP, are attributed with even stronger inflammatory potential, further contributing to inflamed microenvironments (6, 7). Thus, CRP may trigger harm in various human diseases (8–11). Nevertheless, the specific contribution of CRP in human disease remains a matter of debate (10, 12–14).

Coronary artery disease, with acute ST-segment Elevation Myocardial Infarction (STEMI) as one of its manifestations, is an inflammatory disease (15). STEMI experiments in animals confirmed CRP deposits in ischemic myocardium (8). In humans, post-mortem specimens of myocardial tissue after STEMI exhibit CRP deposits colocalizing with activated complement fragments (16). These findings suggest augmented tissue damage *via* complement and macrophage activation after STEMI (17). Clinically, STEMI is frequently accompanied by a steep increase of CRP serum levels during the acute phase response after the onset of myocardial ischemia reaching its maximum plasma concentration between 36 and 72 h (18).

Recently, a CRP adsorber for apheresis has been developed allowing highly selective removal of CRP from patients' blood plasma. Beforehand, the authors had demonstrated the potential benefit of extracorporeal reduction of elevated CRP levels in sham controlled pre-clinical studies using an experimental STEMI pig model. Pigs, in terms of anatomy, size and circulation, largely correspond to the biological situation of humans. These pre-clinical studies had shown a significant positive influence of CRP apheresis on myocardial infarct size and LV function as determined by CMR and macroscopic analyses (17).

Given this background, the CAMI-1 study was designed to test the hypothesis, whether specific depletion of CRP is a means to reduce myocardial infarct size in humans. In addition, CAMI-1 investigates the feasibility and safety of adsorber-based CRP removal in acute STEMI. STEMI was selected for several reasons: (a) despite recent improvements, STEMI mortality is still high, demanding innovative approaches to further improve survival (19), (b) pre-clinical data on CRP's pathological function in AMI are consistent in several species (8, 9, 11), and (c) short time application of CRP removal by apheresis may aid to obtain a clinical benefit (17).

Aim of this pilot study was to explore the relationship between CRP concentration and myocardial infarct size and left ventricular function, to demonstrate the benefit of CRP reduction on these myocardial features and to evaluate the feasibility and safety of CRP apheresis.

## Methods

### Study Design and Patient Population

**Figure 1:**
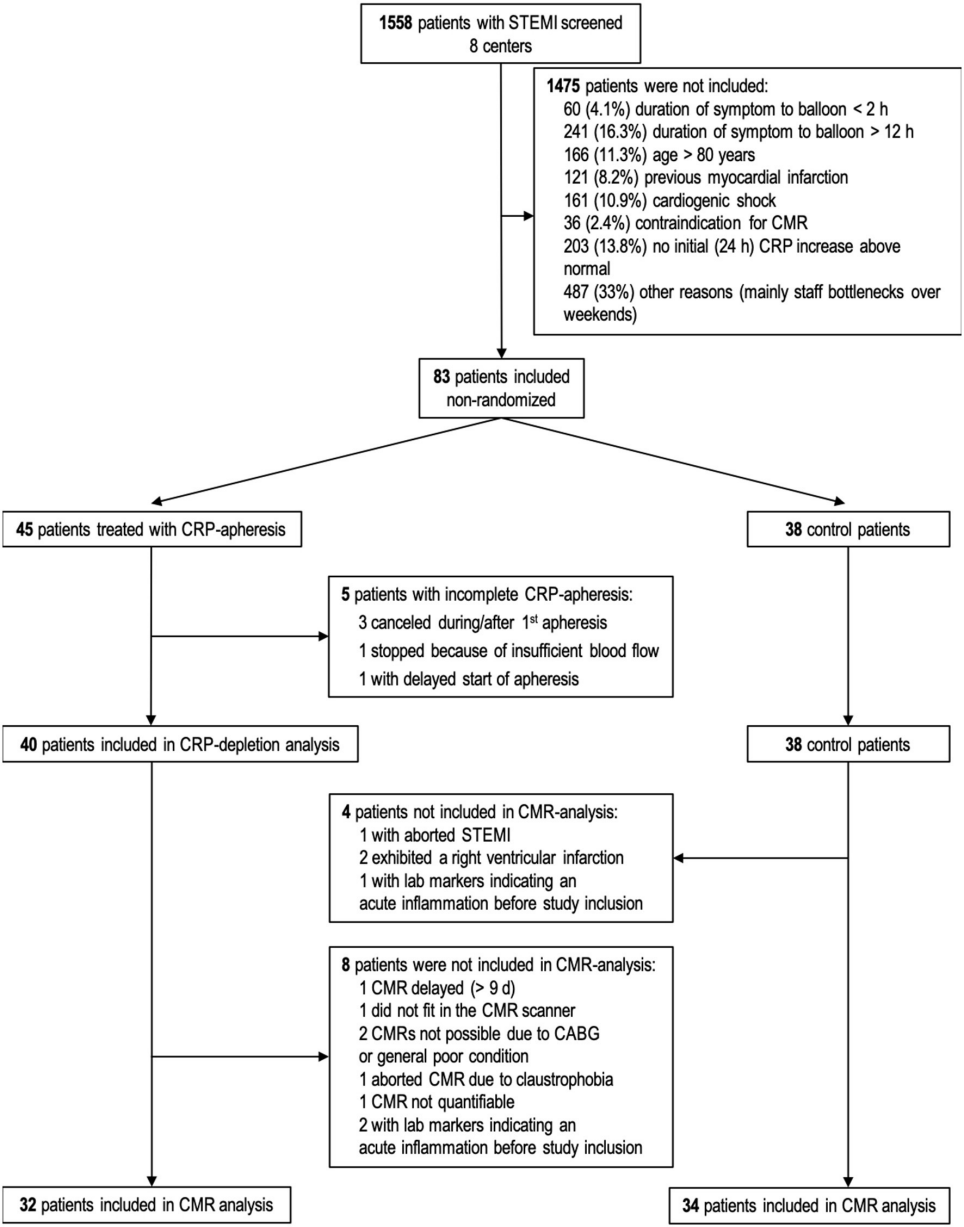
Flow chart of patients' recruitment and drop outs. Sixty-six patients (32 CRP apheresis group and 34 control group) attended final statistical analysis for CMR parameters. CABG, Coronary artery bypass graft; STEMI, ST-segment elevation myocardial infarction; CMR, cardiac magnetic resonance; CRP, C-reactive protein.

CAMI-1 is a prospective, explorative, non-randomized, multicenter pilot trial of consecutive patients with acute STEMI that underwent therapeutic CRP apheresis at 8 sites in Germany between November 2015 and November 2018. Mandatory requirement for inclusion was guideline-oriented therapy with successful percutaneous coronary intervention (PCI) as primary treatment. As CRP apheresis is quite intensive regarding time and personnel of clinical centers, non-randomization was deliberately chosen for this initial pilot study to optimize recruiting rates. The local infrastructure in most of the clinics permitted the inclusion of apheresis patients from Monday to Wednesday, greatly limiting the recruiting efficiency (see also Figure 1).

Further, the original statistical strategy planned to analyze the patients as matched pairs, which resulted in a calculated patient number (sample size) of 34 patients per group. The statistical power was set as 80% and the significance level as 5%. Toward the end of the study it was realized that inclusion of matched pair patients was not feasible in the clinic and in the setting of a multi-center study. Therefore, the statistical analysis was changed and is described in detail under section **Statistical Analysis**.

Inclusion criteria were: age range 18–80 years, STEMI as defined by ESC guidelines (20), Killip-class ≤ II at admission, symptom onset-to-balloon time 2–12 h, TIMI (Thrombolysis In Myocardial Infarction classification) III flow after PCI, patient informed consent and legal capacity.

Exclusion criteria were: previous myocardial infarction or bypass surgery, acute infectious diseases, cardiogenic shock, hemodialysis, malignant or chronic inflammatory diseases, pregnancy or lactation period, contra-indications for magnetic resonance imaging, impossibility of follow up examinations and participation in other clinical trials. The trial complies with the Declaration of Helsinki and was approved by the national regulatory authority and the ethics committees of the participating centers.

#### Trial Protocol

All patients received standard therapeutic treatment for STEMI according to the guidelines of the European Society of Cardiology (20). Thereafter, patients were assigned to either the apheresis group or to the control group. Apheresis was performed if the patient confirmed to the apheresis and the personal and technical resources were available. Participants of the treatment arm received 2-3 apheresis sessions depending on the CRP concentration. Enrolled patients underwent CRP measurements at least daily until end of the study. CRP of patients who received apheresis was measured directly before and after apheresis additionally. They were further examined for adverse events during apheresis procedures. Infarct parameters were measured with cardiovascular magnetic resonance (CMR) between the 2nd and 9th day (CMR1) and week 12 ± 2 (CMR2) after STEMI. Patients were followed-up for 12 months to report major adverse cardiac events (MACEs).

#### Outcomes

Primary outcome parameter was a reduced infarct size in apheresis patients compared to control patients according to the two CMR measurements. Secondary outcomes were the safety of the procedure in AMI patients (measured by the incidence of side effects), improved left ventricular ejection fraction (LVEF, measured by CMR) and incidence of MACEs 6 and 12 months after STEMI. Definition of MACEs: death, reinfarction or stroke, instable angina pectoris, congestive heart insufficiency, which requires stationary treatment and coronary revascularization.

### CRP Quantification

CRP was quantified with the respective standard procedures of the routine diagnostic laboratories of the clinical sites, which are accredited by German/European law. Within the respective clinical centers, the procedure remained the same throughout the whole study. The CRP concentration determined during apheresis was adjusted by the hematocrit of the same blood sample. In detail, the hematocrit before and after apheresis was measured and the percentage difference calculated to account for blood loss and/or infusion of liquid as e.g., physiological salt solution during the procedure. The measured CRP concentration was then normalized to this difference.

CRP kinetics of each patient are included in the Supplementary Material. As expected, absolute CRP concentrations differed widely between patients. To identify and compare the CRP exposure of each patient, the area under the curve (AUC) of each kinetic for 72 h after symptom onset was calculated, which represented the individual CRP exposure.

In addition, the increase of the CRP concentration during the first 32 h after symptom onset (mean 31.38 ± 5.9 h) was used as rate of CRP over ~20 h (mean 19.65 ± 6.4 h). This rate (“CRP gradient”) was calculated as the delta between the CRP concentration at the time points 36 ± 12 and 12 ± 12 h, divided by the time frame (in h) between the two measurements. The CRP gradient of the apheresis group needed to be determined from CRP quantifications before the first CRP apheresis.

### CRP Apheresis

CRP apheresis has been described in detail before (21). For CRP depletion, a reusable single-adsorber-system was used (PentraSorb® CRP, Pentracor GmbH, Germany). The PentraSorb resin is capable of CRP depletion from blood plasma with an efficiency of up to 94% (22). Patients received two CRP aphereses after PCI. The 1st apheresis started 10–36 h after onset of symptoms, the 2nd apheresis took place 24 ± 12 h after the 1st apheresis. A 3rd apheresis was performed if ~12 h after the end of the 2nd apheresis the CRP-concentration rose to values above 30 mg/L. Blood was drawn *via* peripheral venous access (cubital vein). Up to 6,000 mL of plasma was processed in each apheresis, preferentially in 12 cycles of 500 mL (change of loading and regeneration of the adsorber). Patients of the control group received no CRP apheresis and were treated according to the standard therapeutic guidelines for STEMI (20).

### CMR

Cardiovascular magnetic resonance (CMR) was performed locally between the 2nd and 9th day (CMR1) and week 12 ± 2 (CMR2) after STEMI. CMRs followed a rigorous protocol locally and were thereafter evaluated centrally and blinded by the CMR-Core-Lab at the Department of Internal Medicine/Cardiology, German Heart Center Berlin (DHZB), Germany.

#### Image Acquisition

All patients were examined with a clinical 1.5 CMR scanner. Cine and late-enhancement (LGE) imaging were performed. Cine images (25–30 cardiac phases) were acquired using a retrospectively gated cine-CMR in cardiac short-axis, vertical long-axis and horizontal long-axis orientations using a steady-state free precession (SSFP) sequence. The short axis slices covered the entire left ventricular myocardium from the mitral valve to the apex (slice thickness: 8 mm with no gap). A contrast-enhanced inversion recovery gradient echo sequence was then performed in the same slice locations using the same thickness/gap parameters as steady-state free precession images (TI: optimized to null remote myocardium signal intensity) around 10 min after intravenous injection of 0.2 mmol/kg BW of a gadolinium-based contrast agent.

#### Image Analysis

Image analysis of left ventricular function and mass was performed offline using commercially available software (see the section Methods in the Supplementary Material). Left ventricular myocardial strain was analyzed in accordance with a recent consensus document for the quantification of LV function (23). (Details see in the Supplementary Material). Outcomes of interest evaluated for all patients were infarct size, LVEF, longitudinal and circumferential strain.

### Statistical Analysis

Baseline characteristics and CMR parameters were analyzed descriptively by routine methods, which are described in detail in the respective table legends.

CRP kinetics for each patient are represented *via* calculation of AUC (CRP 0–72 h). CRP gradient (increase of CRP concentration within first 32 h) was calculated for each patient and either plotted normally or as ln(CRP gradient) for better graphical presentation of data points.

The analysis of the primary and secondary outcome measures infarct size and LVEF between control patients and apheresis patients turned out to be more complex as a direct comparison was not possible. Linear regression of ln(CRP gradient) vs. four different CMR parameters (infarct size, LVEF, longitudinal, and circumferential strain) for both CMRs was therefore statistically modeled and evaluated.

As this was a non-randomized study, it was evaluated by propensity score analysis whether the allocation of patients to the apheresis/control group was influenced by possible confounding variables. Propensity was estimated based on the following variables: time between onset of symptoms and percutaneous coronary intervention including a stent (time to stent), age and infarct location (anterior vs. posterior). These three variables were chosen as they are established clinically relevant factors strongly related to the infarct outcome of the patients (24–26).

For the conditional probability *p* = P (Apheresis|patient-characteristics) the propensity score was estimated using a logit-model (x_i_ representing patient-characteristics, p estimated using glm-routine in R).

$$\begin{array}{l} Propensity=\frac{e^{\beta 0\;+\;\beta 1\;*\;timetostent\;+\;\beta 2\;*\;infarctloc\;+\;\beta 3\;*\;age}}{1+\;e^{\beta 0\;+\;\beta 1\;*\;timetostent\;+\;\beta 2\;*\;infarctloc\;+\;\beta 3\;*\;age}} \end{array}$$

The estimated propensity scores were then taken to correct the used linear models by means of regression adjustment.

Linear model (infarct parameter~gradient) vs. Linear model (infarct parameter~gradient + propensity).

As there was no difference between the “raw” and the “propensity-corrected” linear models, the “raw” models were used for further analyses.

All statistical analyses were performed using R version 3.6.1.

### Study Approval

The CAMI-1 study was approved by the Ethics Committee No.: 042/15 (I), Medical Association Schleswig-Holstein, Germany. The study was registered under the number WHO ICTRP: DRKS00008988. Written informed consent was received from all participants prior to inclusion in the study.

## Results

In the 3-year period of CAMI-1 recruitment, a total of 1,558 patients diagnosed with STEMI were screened. Finally, 83 patients were enrolled in the trial (Figure 1). Lastly, 66 patients (32 in CRP apheresis group and 34 in control group) were included in the final statistical analysis (reasons for exclusion see Figure 1). Patient characteristics are reported in Table 1. Though no randomization was performed in this pilot study, both groups appeared largely comparable at baseline. The only two variables which were significantly higher in the control group were BMI (*p* = 0.05) and diabetes (*p* = 0.03; Table 1).

**Table 1:** Baseline characteristics of the study population.

**Characteristic**	**CRP apheresis group**	**Control group**	***p-*value^*a*^**
No. of cases	32	34	
Age—years	56 ± 10.6	59 ± 9.6	0.2
Male—no. (%)	25 (78.1)	30 (88.2)	0.3
**Cardiovascular risk factors**			
BMI*	29.3 ± 4.5	27.2 ± 4.1	0.05
Smoking—packages per year	20.5 ± 20.8	21.9 ± 16.2	0.8
Smoker—no. (%)	23 (71.9)	28 (82.4)	0.38
Hypertension—no. (%)	20 (62.5)	17 (50)	0.3
Diabetes—no. (%)	10 (31.3)	3 (8.8)	0.03
Dyslipidemia—no. (%)	18 (56.3)	19 (55.9)	1
Pre-infarction angina^#^–no. (%)	9 (28.1)	14 (41.2)	0.3
**Killip-class on admission—no. (%)**			
1	28 (87.5)	29 (85.3)	1
2	4 (12.5)	5 (14.7)	
Anterior infarction—no. (%)	17 (53.1)	19 (55.9)	1
**No. of diseased vessels^†^—no. (%)**			
1 vessel	16 (50)	13 (38.2)	
2 vessels	12 (37.5)	14 (41.2)	0.5
3 vessels	4 (12.5)	7 (20.6)	
Onset of symptoms to stent—h	4.7 ± 2.8	4.4 ± 2.5	0.7
CRP_1_ on admission—mg/L	5.0 (1.2–32)	3.1 (0.4–23)	0.2
Onset of symptoms to CRP_1_–h	3.7 ± 2.6	3.8 ± 2.7	0.8
CRP_3_ ~24 h after onset of symptoms—mg/L	15.0 (5.2–102)	16.1 (2.5–150)	0.7
Onset of symptoms to CRP_3_–h	22.5 ± 3.2	23.2 ± 2.7	0.4

a *The statistical comparability of both patient groups was demonstrated both univariately, i.e., feature by feature, and multivariate based on a logistic model. As the table shows, significant univariate differences were observed only for BMI and diabetes. In BMI and diabetes, the difference between the two groups is also significant in favor of the controls. Data are presented as mean ± SD, n (%) or median (range). P-values were calculated using student's t-test with bootstrap analysis or Fisher's exact-test*.

* *Body-mass index is the weight in kilograms divided by the square of the height in meters*.

# *Angina had to occur <1 week before symptom onset of STEMI*.

† *Stenosis ≥ 50%*.

All apheresis sessions were well-tolerated without severe apheresis-associated side effects. Only in a few cases patients reported mild to moderate symptoms, which are listed in Table 2. During one apheresis, a typical angina pectoris occurred, most probably due to simultaneous strong anguish of mind. In this case, treatment could be continued the next day without complications and only a slight feeling of pressure in the chest. All listed side effects correspond to known events for extracorporeal apheresis and were considered not to be device related.

**Table 2:** Overview of reported adverse events during apheresis treatments.

**Adverse event**	**CTC grade^*a*^**	**No. of patients**
Pain (pressure in chest)	1	3
Blood pressure drop	2	1
Blood pressure rise	1	1
Pain (at venous needle)	1	1
Headache	2	1
Edema at hand	1	1
Temperature increase	1	1
Sweating	2	1

a *All reported adverse events were mild or moderate side effects and correspond to known events for extracorporeal apheresis. They were evaluated not to be device related. CTC Common Toxicity Criteria (0–4)*.

In the 12 months follow-up, 3 adverse cardiac events were reported in the control group (death, coronary revascularization, pacemaker).

### Performance and Timing of CRP Apheresis

Forty patients with completed CRP apheresis were enrolled. They received a total of 86 apheresis sessions. In average, the 1st apheresis started 27.1 ± 6.5 h after onset of symptoms at a median CRP concentration of 23.0 mg/L (range 9–279) and led to a mean reduction of serum CRP levels of 50.3 ± 16.4%. The 2nd apheresis resulted in a mean reduction of 56.9 ± 13.1%. Seven patients received a 3rd apheresis due to persistently high levels of CRP (>30 mg/L) leading to a mean CRP-reduction of 47.8 ± 11.9%. Averaged over all apheresis sessions, CRP depletion amounted to 53.0 ± 15.1%. CRP apheresis was performed during a phase of a sharp increase of CRP plasma concentration.

Average time interval between start of 1st and 2nd CRP apheresis was 21.1 ± 2.9 h. Mean duration of all apheresis sessions was 4.9 ± 0.8 h. During this time, 5,900 ± 400 mL of blood plasma was processed (mainly in 12 loading cycles of 500 mL), corresponding to 1.8 ± 0.2 patient plasma volumes.

Selectivity of CRP apheresis with regard to other plasma proteins was investigated using γ-globulin and fibrinogen as exemplary parameters: we observed an average reduction of total protein concentration, γ-globulin and fibrinogen after apheresis of 8.4 ± 6.2, 9.4 ± 6.0, and 6.4 ± 12.2%, respectively.

### CRP Gradient and Area Under the Curve

**Figure 2:**
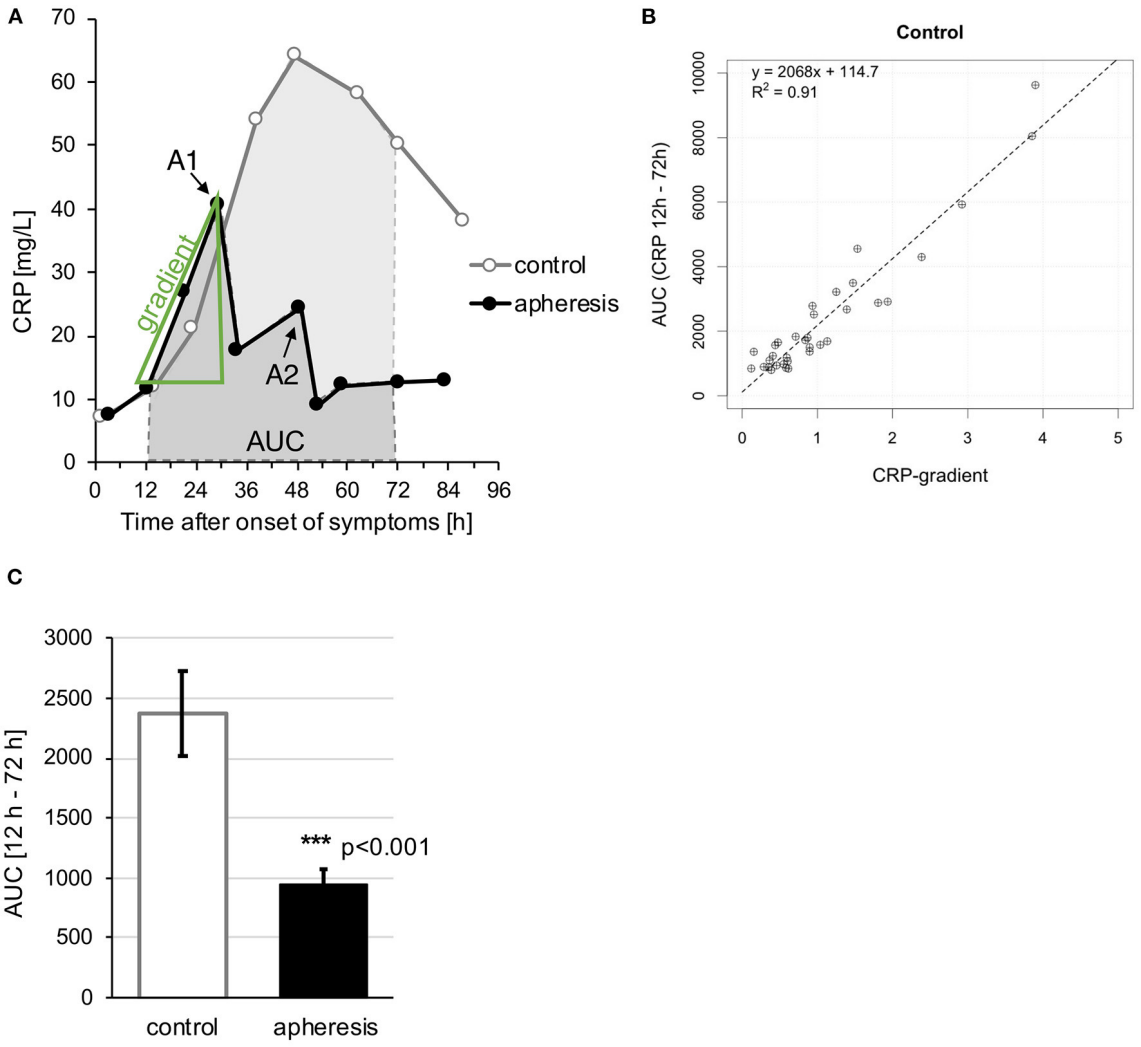
“CRP gradient” and “Area Under the Curve (AUC).” **(A)** Exemplary schematic analysis of CRP kinetics. Typical kinetics with (“apheresis”) and without (“control”) CRP apheresis. From the initial increase of the CRP curve a gradient was calculated as well as the AUC between 12 and 72 h after onset of symptoms. A1 and A2 = apheresis sessions. **(B)** Comparison of CRP gradient (mg/L/h) with AUC between 12 and 72 h. High regression coefficient of >0.9 for the control group. The relationship of AUC and gradient is shown in more detail in Supplementary Figure 1. **(C)** The graph shows the mean AUC of each group ± SEM. Highly significant AUC reduction due to CRP apheresis (*p* < 0.001).

According to the study's hypothesis that CRP causes concentration-dependent myocardial damage, further analyses included patients' CRP concentrations. This raises the question how to estimate the prospective CRP concentration in patients undergoing CRP apheresis, which efficiently lowered the CRP concentration. We used the increase of the CRP kinetic in the first up to 36 h (before the apheresis) to predict the Area Under the Curve of CRP (total quantity CRP over 12–72 h, AUC) and calculate a corresponding “CRP gradient.” In the control group, the kinetics of CRP plasma levels (i.e., the “CRP gradient”) within the first hours after STEMI were almost perfectly predictive (*R*^2^ = 0.91) for the patient's total CRP exposure correlating significantly with CRP total quantity over 12–72 h (AUC) (Figures 2A,B). The CRP gradient represents the post-infarction CRP kinetic and allows valid prediction of expected CRP exposure to the patient before apheresis. Consequently, we used the CRP gradient as a prognostic tool to directly correlate CRP plasma concentrations with CMR parameters in both groups.

AUC in the apheresis group differed significantly from AUC in the control group (Figure 2C). The reduction of AUC due to CRP apheresis was ~62.5%.

### CMR Parameters

The CMR parameters were recorded at two time points. In the control group, 2 patients lacked a CMR2 because of severe events (1 death, 1 pacemaker), since they obviously could not be subjected to a 12-week CMR. One control patient refused the 2nd CMR. In the apheresis group, one patient lacked a CMR2, since CMR1 in this patient did not show any infarct area. Further, one apheresis patient refused the 2nd CMR and one was lost to follow-up (see also Table 3).

**Table 3:** Overview of the number of patients for different CMR parameters.

**Readout**	**Number of patients (Control/Apheresis)**
CMR 1	66 (34/32)
CMR 2^a^	60 (31/29)
Infarct size 1	66 (34/32)
Infarct size 2	58 (29/29)
LVEF 1	66 (34/32)
LVEF 2	60 (31/29)
Longitudinal strain 1	66 (34/32)
Longitudinal strain 2	59 (30/29)
Circumferential strain 1	66 (34/32)
Circumferential strain 2	60 (31/29)
Area at risk 1	46 (23/23)
Myocardial Salvage Index	42 (21/21)

a *Originally, the 2nd CMR was to be taken after 10–14 weeks. This was the case in 42 patients (20 control/22 apheresis). Accordingly, in 18 patients, the 2nd MRI was performed after more than 14 weeks (11 control/7 apheresis)*.

Supplementary Table 1 summarizes the results of both CMRs for both groups.

### Propensity Analysis

The mean propensity scores for both groups were: 0.47 (control) and 0.5 (apheresis). They did not differ according to student's *t*-test with subsequent bootstrap analysis (*p* = 0.14). Therefore, according to the estimated propensity scores, the decision to place patients in the treatment arm was not confounded by the three variables “time to stent,” “age,” and “infarct location.”

The linear models were nevertheless corrected with the estimated propensity score in order to compare non-adjusted with adjusted models. Supplementary Figures 2, 3 show that both models highly correlate in regard to all evaluated CMR parameters for the first and second CMR. Subsequent ANOVA analysis further showed that for all CMR parameters the models did not differ significantly.

Therefore, the unadjusted models were used for further analysis and interpretation.

Additionally, it was analyzed whether baseline characteristics (e.g., age, BMI, diabetes, time to stent, all listed in Table 1) of patients (especially control patients) correlate with the size of myocardial damage. No correlation could be found for the baseline characteristics (data not shown).

### CMR Parameters and CRP Gradient

We showed that the calculated CRP gradient ideally represents the CRP AUC of control patients and predicts the hypothetical CRP AUC in apheresis patients, if apheresis had not depleted CRP. Hence, in order to investigate the impact of CRP depletion on left ventricular parameters, we correlated the CMR parameters to the CRP gradient.

#### Myocardial Infarct Size and CRP Gradient

**Figure 3:**
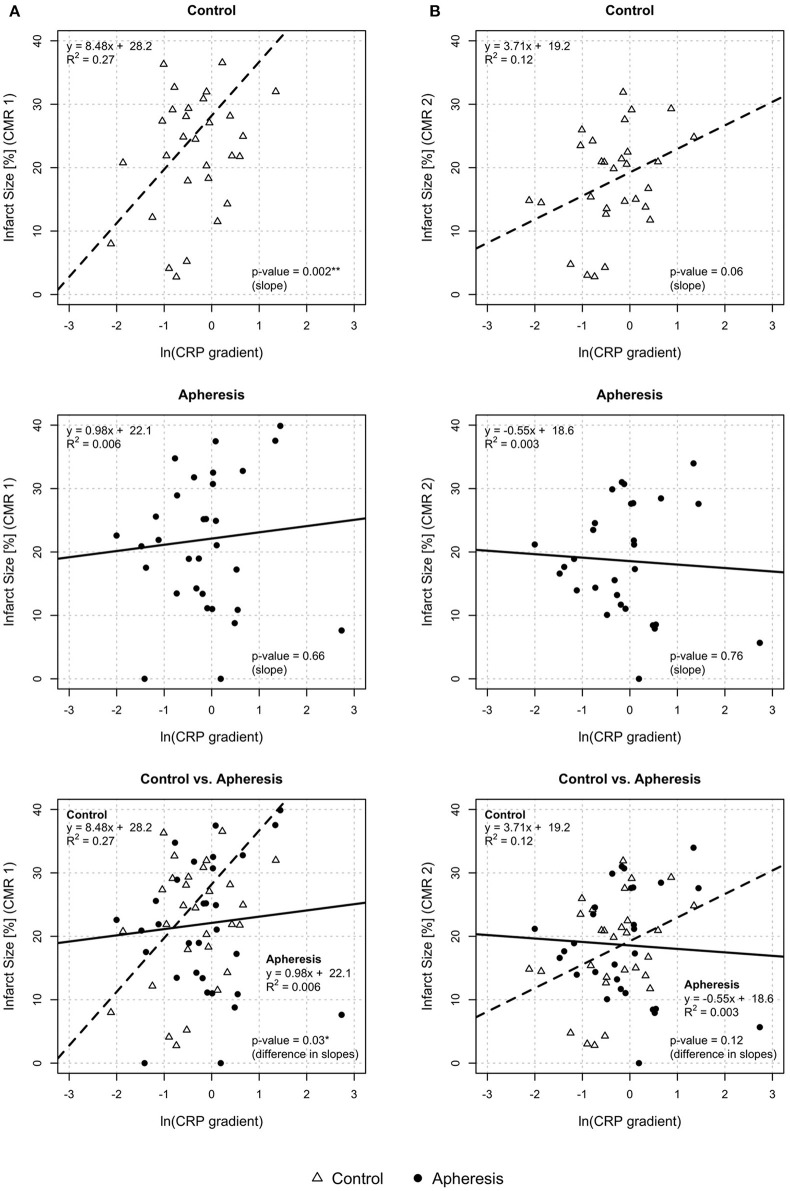
Myocardial infarct size and CRP gradient [ln(CRP gradient)] at CMR1 **(A)** and CMR2 **(B)**. CMR1—Control group: Highly significant increase of infarct size with an increase of CRP gradient (*p* = 0.002). Increase of infarct size by 8.5% with an increase of ln(CRP gradient) by 1 unit. Apheresis group: Small increase of infarct size with an increase of CRP gradient (*p* = 0.66). Importantly, the two linear models differ significantly (*p* = 0.03). CMR2—Control group: Non-significant increase of infarct size with an increase of CRP gradient (*p* = 0.06). Increase of infarct size by 3.7% with an increase of ln(CRP gradient) by 1 unit. Apheresis group: Small loss of infarct size with an increase of CRP gradient (*p* = 0.76). The two linear models differ, but not significantly (*p* = 0.12).

In Figure 3A (CMR1), myocardial infarct size estimated by CMR has been plotted against CRP gradient, separately for both groups. This was based on the linear models relating the infarct size to the CRP gradient. In control patients, a significant increase of infarct size with an increase of CRP gradient was observed (*p* = 0.002) but not in the apheresis group (*p* = 0.66). The *R*^2^ = 0.27 in the controls showed a correlation between increasing CRP concentration and increasing infarct size. In the apheresis group, the *R*^2^ = 0.006 showed that there is no linear correlation between increasing CRP concentration and increasing infarct size. Interestingly, no myocardial infarct area was observed in two patients of the apheresis group. The two linear models differ significantly (*p* = 0.03).

In Figure 3B (CMR2), myocardial infarct size has been plotted against CRP gradient. In control patients, a non-significant increase of infarct size with an increase of CRP gradient was observed (*p* = 0.06) but no increase was observed in the apheresis group (*p* = 0.76). The *R*^2^ = 0.12 in the controls showed only a slight correlation between increasing CRP concentration and increasing infarct size. In the apheresis group, the *R*^2^ = 0.003 showed that there is no correlation between increasing CRP concentration and increasing infarct size. Moreover, the correlation was lost. No myocardial infarction area was observed both here and in CMR1 in one of the two 0% infarct size patients of the apheresis group with a second CMR. The two linear models do not differ significantly (*p* = 0.12). This may be due to missing CMR2-data, as mentioned above.

**Figure 4:**
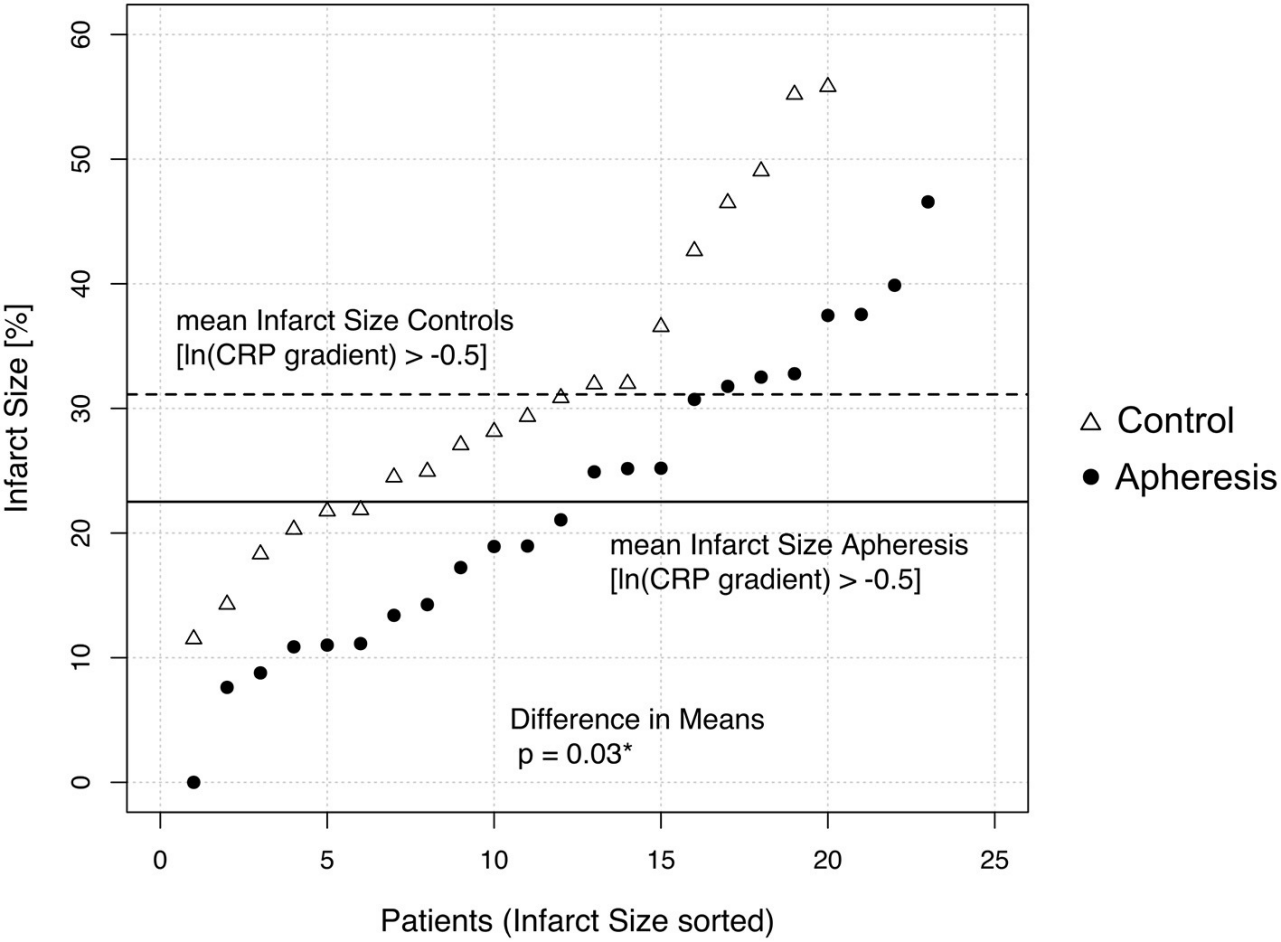
Difference of the Infarct Size at CMR1 between control and apheresis patients with high CRP gradient. Only patients with a CRP gradient higher than 0.6 (>ln(gradient)−0.5) were analyzed and sorted according to their infarct size. Mean Infarct sizes are indicated with dotted (control) or black (apheresis) line. Means differ significantly (*p* = 0.03 after Student's *t*-test).

Obviously, the higher the CRP gradient the more patients benefit from CRP apheresis. By considering only patients with a gradient > 0.6 (≜ ln gradient −0.5) one can directly compare both groups and observe a significant difference in infarct size (31% in controls and 22.5% in apheresis patients; *p* = 0.03 after Student's *t*-test; Figure 4). The gradient of 0.6 corresponds to a peak CRP concentration of ~22 mg/L. Other authors have published that this is the CRP threshold for the difference from a small to a large infarct area (27–31).

#### LVEF and CRP Gradient

**Figure 5:**
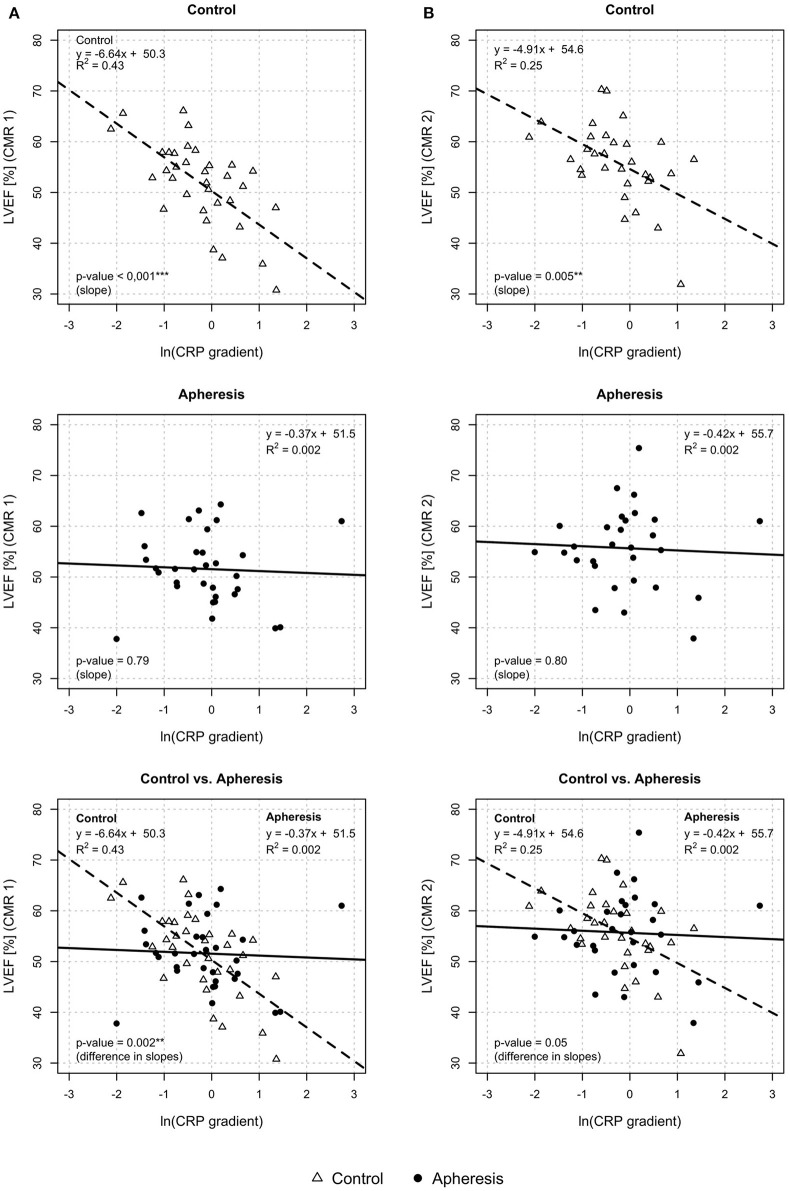
Left ventricular ejection fraction (LVEF) and CRP gradient [ln(CRP gradient)] at CMR1 **(A)** and CMR2 **(B)**. CMR1—control group: Highly significant loss of LVEF with increasing values of the CRP gradient in the control group (*p* < 0.001). Increase of ln(CRP gradient) by 1 unit reduces the LVEF by 6.6%. Apheresis group: Small loss of LVEF with an increase in CRP gradient in the apheresis group (*p* = 0.79). Importantly, the two linear models differ highly significantly (*p* = 0.002). CMR 2—control group: Significant loss of LVEF with increasing values of the CRP gradient in the control group (*p* = 0.005). Increase of ln(CRP gradient) by 1 unit reduced the LVEF by 4.9%. Small loss of LVEF with an increase in CRP gradient (*p* = 0.8). Importantly, the two linear models differ significantly (*p* = 0.05).

In Figure 5A (CMR1), LVEF was plotted against CRP gradient, separately for both groups. In control patients, a significant loss of LVEF with increasing values of the CRP gradient was observed (*p* < 0.001) but not in the apheresis group (*p* = 0.79). The *R*^2^ = 0.43 in the controls showed a correlation between increasing CRP concentration and decreasing LVEF. On the other hand, in the apheresis group the *R*^2^ = 0.002 showed that there is no correlation between increasing CRP concentration and decreasing LVEF. The correlation was lost. The two linear models differ significantly (*p* = 0.002).

In Figure 5B (CMR2), LVEF was plotted against CRP gradient, separately for both groups. In control patients, a significant loss of LVEF with increasing values of the CRP gradient was observed (*p* = 0.005) but not in the apheresis group (*p* = 0.8). The *R*^2^ = 0.25 in the controls showed a correlation between increasing CRP concentration and decreasing LVEF. On the other hand, in the apheresis group the *R*^2^ of 0.002 showed that there is no correlation between increasing CRP concentration and decreasing LVEF. Again, the correlation was lost. The two linear models differ significantly (*p* = 0.05).

#### Longitudinal and Circumferential Strain and CRP Gradient

**Figure 6:**
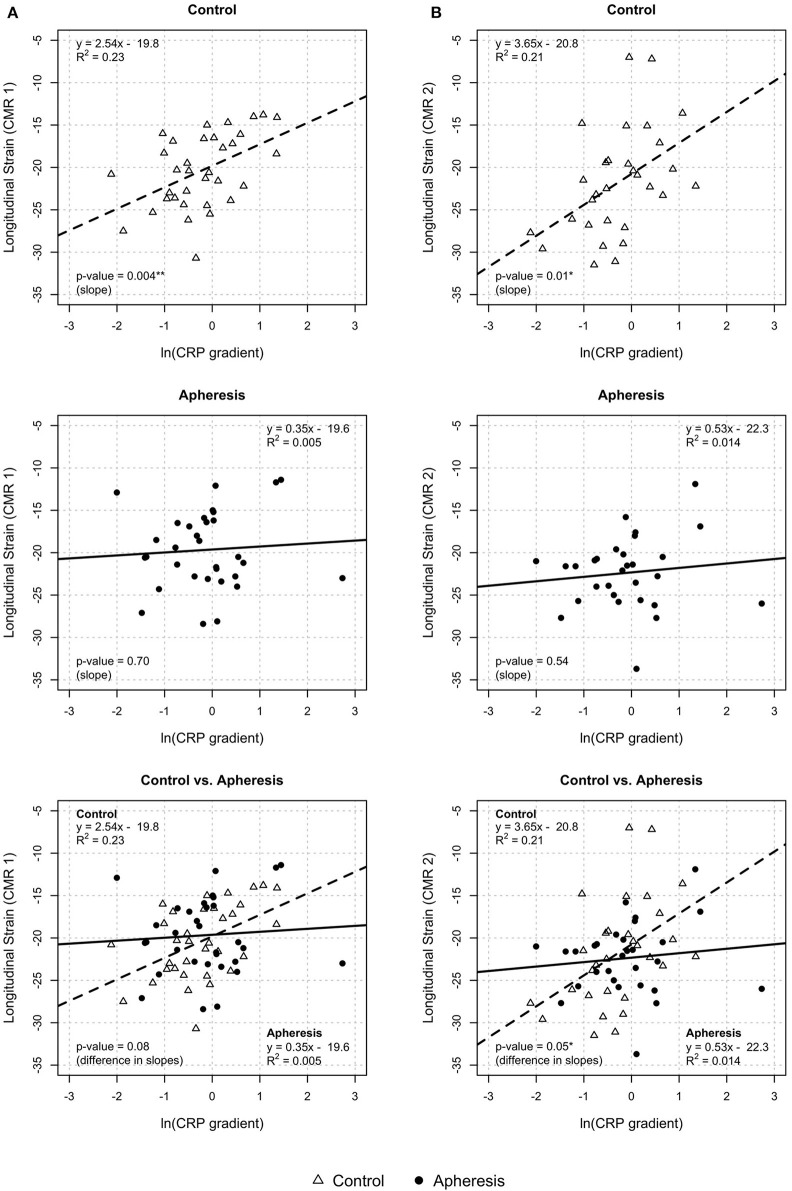
Longitudinal strain CRP gradient [ln(CRP gradient)] at CMR1 **(A)** and CMR2 **(B)**. CMR1—control group: Highly significant increase of longitudinal strain with increasing values of CRP gradient (*p* = 0.004) and increase of ln(CRP gradient) by 1 unit results in an increase of longitudinal strain by about 2.5 units. Apheresis group: Small increase of longitudinal strain with increase of CRP gradient (*p* = 0.7). Here, there is no statistically significant difference between groups (*p* = 0.08). CMR2—control group: significant increase of longitudinal strain with increasing values of CRP gradient (*p* = 0.01). Increase of ln(CRP gradient) by 1 unit results in an increase of longitudinal strain by about 3.6 units. Apheresis group: Small increase of longitudinal strain with increase of CRP gradient (*p* = 0.54). There is a statistically significant difference between groups (*p* = 0.05).

In control patients, a significant increase of longitudinal strain with increasing values of the CRP gradient was observed (*p* = 0.004, Figure 6A) but not in the apheresis group (*p* = 0.7). The *R*^2^ = 0.23 in the control group showed a correlation between increasing CRP concentration and increasing longitudinal strain. In the apheresis group, *R*^2^ = 0.005 showed that there is no correlation between increasing CRP concentration and increasing longitudinal strain. The correlation was lost. The two linear models do not differ significantly (*p* = 0.08).

In Figure 6B (control patients, CMR2), a significant increase of longitudinal strain with increasing values of the CRP gradient was observed (*p* = 0.01) but not in the apheresis group (*p* = 0.54). The *R*^2^ = 0.21 in the controls showed a correlation between increasing CRP concentration and increasing longitudinal strain. In the apheresis group, *R*^2^ = 0.014 showed that there is no correlation between increasing CRP concentration and increasing longitudinal strain. The correlation was lost. The two linear models differ significantly (*p* = 0.05).

**Figure 7:**
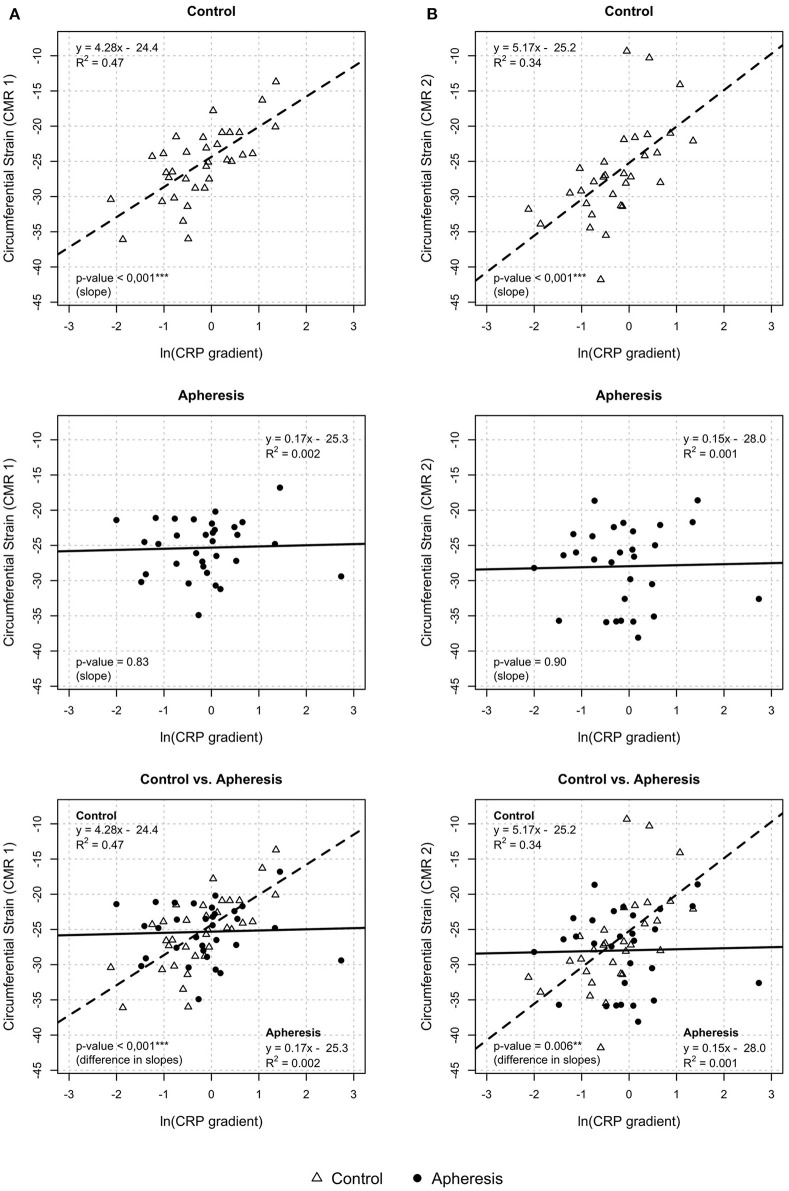
Circumferential strain and CRP gradient [ln(CRP gradient)] at CMR1 **(A)** and CMR2 **(B)**. CMR 1—control group: Highly significant increase of circumferential strain with increasing values of the CRP gradient (*p* < 0.001). Increase of ln(CRP gradient) by 1 unit led to an increase of circumferential strain by about 4.3 units. Apheresis group: Only small increase in circumferential strain with increasing CRP gradient (*p* = 0.83). Importantly, the two linear models differ highly significantly (*p* < 0.001). CMR 2—control group: Highly significant increase of circumferential strain with increasing values of the CRP gradient (*p* < 0.001). Increase of ln(CRP gradient) by 1 unit led to an increase of circumferential strain by about 5.2 units. Apheresis group: No significant increase in circumferential strain with increasing CRP gradient (*p* = 0.9). Importantly, the two linear models differ highly significantly (*p* < 0.006).

Circumferential strain showed a highly significant rise with increasing values of the CRP gradient in control patients (*p* < 0.001, Figure 7A, CMR1) but not in the apheresis group (*p* = 0.83). The *R*^2^ = 0.47 in the controls showed a correlation between increasing CRP concentration and increasing circumferential strain. On the other hand, in the apheresis group *R*^2^ = 0.002 showed that there is no correlation between increasing CRP concentration and increasing circumferential strain. The correlation was lost. The two linear models differ significantly (*p* = 0.001).

In CMR2, the circumferential strain showed a highly significant rise with increasing values of the CRP gradient in control patients (*p* < 0.001, Figure 7B) but not in the apheresis group (*p* = 0.9). The *R*^2^ = 0.34 in the controls showed a correlation between increasing CRP concentration and increasing circumferential strain. In the apheresis group *R*^2^ = 0.001 showed that there is no correlation between increasing CRP concentration and increasing circumferential strain. The correlation was lost. The two linear models differ highly significantly (*p* = 0.006).

Taken together, in all endpoints except longitudinal strain (CMR1) and infarct size (CMR2), the differences between the study arms were statistically significant and in favor of the apheresis group.

### Microvascular Obstruction

In control patients, 15 showed a relative MVO > 0 in CMR1 (i.e., 44.1% of controls; median: 1.99%, range: 0.2–7.2%).

In CRP apheresis patients, only 10 showed a relative MVO > 0 in CMR1 (i.e., 31.2%, median: 0.58%, range: 0.24–19.6%). Obviously, patients with CRP apheresis had a noticeable reduction of MVO. However, there was no statistically significant difference between the two groups due to the small total number of patients (*p* = 0.13, Mann-Whitney-Test). No MVO could be detected in CMR2 for any patient.

## Discussion

The CANTOS and COLCOT studies have impressively demonstrated the great role of low-threshold inflammation in the prognosis of STEMI patients with regard to further cardiovascular events. Both IL-1ß-blockade and colchicine have been shown to reduce mortality by lowering inflammation and especially CRP levels (32, 33).

CRP has long been thought to be pathogenetically involved in human cardiovascular disease (11, 13). Until today, this hypothesis could not be confirmed due to the lack of tools to efficiently block or eliminate CRP from the human circulation in an acute setting. Other pre-clinical studies, which efficiently lowered CRP, did not present feasible protocols for the use in patients after AMI. The use of a small-molecule inhibitor in a rat model of AMI was dependent on the administration of this agent before the incident (11). Another study showed successful inhibition of CRP synthesis only after 3 weeks of treatment with the investigated antisense oligonucleotide in humans (34) and with significant reduction of CRP concentration after a minimum of 7 days in rats (35). Further, targeted anti-inflammatory trials as e.g., the application of the IL-6 receptor antagonist tocilizumab after NSTEMI in patients, used the measured CRP-AUC as primary endpoint (36). The newly developed CRP apheresis, after its recent successful “first-in-man”-application, steps into this gap and directly affects the CRP-AUC without detours (17, 22, 37).

No kind of therapeutic apheresis has ever been investigated as acute treatment after AMI. Other comparable apheresis trials include e.g., chronic lipoprotein apheresis therapy to reduce progressive cardiovascular disease (38).

For our study, the setting of acute STEMI was chosen because of substantial pre-clinical data as well as post-mortem observations on CRP's involvement in post-infarction myocardial damage (8, 9, 11, 17). Additionally, the clinical observation of a rise in post-infarct complications associated with high CRP levels suggests that even short-time CRP depletion after acute STEMI may be beneficial (17, 18).

### Correlation Between CRP and Myocardial Infarct Size

The first *part of CAMI-1* corroborates the correlation between systemic CRP concentrations, size of myocardial damage and restriction of left ventricular systolic function. This correlation strongly indicates a pathogenic contribution of CRP in human acute STEMI: higher levels of CRP concentrations correlate with larger sizes of myocardial damage. None of the other enlisted patients' baseline characteristics shows such pathogenic impact. These results of CAMI-1 prompt an approach to interrupt this important correlation.

### Dissolving the Correlation Between CRP and Myocardial Infarct Size

Corresponding to the above mentioned, eliminating sufficient quantities of CRP from the human circulation in STEMI should reduce myocardial damage and improve left ventricular systolic function. *This second part of CAMI-1* states that CRP removal by CRP apheresis in the setting of acute STEMI results in smaller sizes of myocardial damage and improved left ventricular systolic function. By interfering with the natural course of CRP augmentation with associated myocardial destruction, CRP elimination through CRP apheresis could diminish myocardial harm. This effect was most pronounced in patients exhibiting a high CRP exposure.

Moreover, CRP elimination in STEMI through CRP apheresis showed a trend to improve, as further indication of effectiveness, myocardial perfusion by lowering microvascular obstruction (MVO). MVOs are caused by a complex pathophysiology in which high CRP levels might contribute to increased plaque formation (39–41). In STEMI, MVO persists in a considerable number of patients despite rapid resolution of the coronary occlusion by PCI, deteriorating myocardial perfusion and increasing mortality and hospitalization for heart failure within 1 year (42).

### Clinical Significance of CRP Apheresis

The observed dose-response relationship between CRP concentrations and myocardial infarct size and its beneficial modulation by CRP apheresis establishes a fundamental, innovative approach for the treatment of STEMI. As an additional therapeutic measure to the current guidelines, CRP apheresis has the potential to disrupt the ratio “CRP load/myocardial damage” and improve clinical outcome of STEMI patients.

According to recent studies, mortality of STEMI does not decline anymore (19). And despite major advances in therapy (PCI, antithrombotic therapy, etc.) with substantially reduced intra-hospital mortality, medium- and long-term mortality remains unacceptably high. Survivors of STEMI often develop heart failure with high rates of re-admission and suffer from reduced quality of life and prognosis (43). Therefore, protecting and improving LV-function in STEMI is of outmost importance. Here, CRP apheresis offers a promising treatment option.

Up to now, broad and undirected anti-inflammatory approaches in STEMI have failed: neither steroids nor Methotrexate revealed clinical efficacy (44, 45). One reason seems to be that inflammatory processes have important functions in repair and/or vascular remodeling to ensure tissue stability (46). In addition, medical approaches take too long to achieve sufficient reduction of the CRP level. CRP apheresis, however, specifically targets circulating CRP, removes substantial CRP amounts within hours and thus diminishes CRP-mediated myocardial damage. It does not interfere with beneficial vascular repair mechanisms.

CRP apheresis as a targeted anti-inflammatory strategy is based on the current knowledge of the damaging role of complement activation in mediating neutrophil and monocyte recruitment in acute myocardial infarction (47). Inspired by previous animal studies, CAMI-1 has successfully confirmed the efficacy and feasibility of CRP apheresis thus widening the armamentarium of specific anti-inflammatory treatment strategies. Thereby, an advantage of CRP apheresis over typical pharmaceutical measures may be its induction of CRP redistribution from various compartments into the blood by a concentration gradient. As a result, the CRP concentration in the myocardium, for example, decreases substantially.

### Statistical Considerations of Study Analysis

The original design of the study planned a non-randomized assignment of matched patients to the control and apheresis group. This resulted in the described sample sizes and power. Unfortunately, this design was not feasible in this multi-center pilot study and therefore had to be adjusted during the trial. Patients were assigned to the groups without pair matching and depending on the local infrastructure in terms of staffing ability to perform apheresis. The assignment of patients to apheresis/control in study centers should be independent of the known characteristics of the patients according to the available possibilities. As Table 1 shows, this has been achieved with the exception of the characteristics diabetes and BMI. Propensity score analysis further underscores the assumption that both groups are largely comparable. Statistical multi-level modeling otherwise required in multicenter studies is not necessary in this study, since the infarct-relevant characteristics were created centrally and blinded at a CMR-Core-Lab.

We have shown that CRP concentration significantly correlates with the extent of infarct sizes and the extent of the reduction of the LVEF. Treatment with CRP apheresis overrides this correlation, as it significantly decreases CRP AUC. Direct comparison of infarct sizes between groups was statistically not manageable due to failure to provide matched pairs. This was only possible after retrospectively eliminating patients with a low CRP gradient, resulting in a significant decrease of the infarct size in apheresis patients. In order to compare the outcome of control and apheresis patients by including all patients, CAMI-1 needed to predict the prospective CRP load in apheresis patients without apheresis interference. Therefore, CAMI-1 proposes the *CRP gradient* as a tool to predict the CRP kinetic throughout the course of STEMI. Determined within the first 32 h after STEMI and before the start of 1st apheresis, the CRP gradient captures the patients' total CRP AUC over the first 72 h of acute STEMI with high accuracy (i.e., CRP gradient and CRP AUC highly correlate in controls). Thus, the early determination of the CRP gradient identifies STEMI patients with disease-aggravating CRP courses and enables a timely application of CRP elimination by CRP apheresis. Meanwhile, the prognostic relevance of the CRP gradient has been highlighted by other authors (48, 49). Based on this CRP gradient we could show that CRP apheresis obliterates the correlation of CRP gradient with all measured CMR parameters and therefore indirectly show an effect of apheresis on patient outcome.

One of the major limitations resulting from the change in statistical analysis is the loss of statistical power. For 32 and 34 cases of apheresis and controls, respectively, the study achieves a power of about 0.35 in terms of infarct size without cut-off. Under otherwise identical conditions, about *n* = 115 observations per group would be required for a power of 0.8. For a study with a higher number of cases the representativeness of the sample will have to be checked. Power here describes the probability to detect that H_0_: “there is no difference in infarct size measured 2–9 days after the infarct between control and apheresis patients” has to be rejected, and thus confirming the positive effect of CRP apheresis.

Based on these observations, a future, sufficiently powered, randomized controlled trial should be designed and the logistic challenges of the apheresis procedure as well as the CRP gradient should be taken into account.

### Pathophysiological Reflections

Coronary occlusion in the setting of acute STEMI causes primary damage to the myocardium. Therefore, early revascularization is the major goal of therapy. But reperfusion itself contributes to secondary oxidative injury (50). Tertiary damage can be caused by CRP in collaboration with complement, according to studies in various animal species. CAMI-1 shows a pronounced dose-response relationship between CRP concentration and myocardial infarction damage in the control cohort. Interestingly, some patients treated with CRP apheresis had not even minor infarct scars and a normal LVEF. Potentially, the supply bottleneck in ischemic tissue does not immediately cause tissue necrosis, but causes a conversion of the energy metabolism to glycolysis, which leads to an enormous lack of energy for the individual cardiomyocyte. Then, cardiomyocytes go into stunning until the shortage of energy supply is reversed. Cardiomyocytes probably only survive, if they are not labeled by CRP and thereby disposed to phagocytes (51).

### Limitations

The first part of CAMI-1 clearly highlights the correlation between CRP concentrations (measured as CRP AUC) and extent of myocardial damage in STEMI. In its second part, CAMI-1 exhibits limitations. First and foremost, CAMI-1 is a non-randomized pilot study, and the results are currently inconclusive. Future controlled randomized trials have to definitely prove or disprove CRP's contribution to myocardial damage in STEMI. Secondly, the number of 66 patients included in the final analysis is relatively low. But with an unambiguous dose-response effect of CRP concentration on myocardial damage observed in the control cohort we see an unequivocal loss of this correlation in the apheresis group. Thirdly, the patients' cohort is not representative for all STEMI patients, because only 5% of 1,558 patients screened were finally eligible for the trial. This has its reasons in the proof of concept nature of a pilot trial with careful patient selection. For example, patients with cardiogenic shock were excluded due to safety reasons, although particularly these patients might benefit in future trials. Fourthly, CAMI-1 included only patients with a first AMI 2 to 12 h after the beginning of the symptoms. This narrow time window was chosen to recruit patients with comparable duration of myocardial stunning after AMI, since prognosis depends on the extent of stunning of myocardium after AMI (19). In our study, 20% of STEMI patients were outside this time window.

Fifthly, 33% of STEMI patients did not undergo CRP apheresis because hospitals lacked personal resources for this new and technically special treatment. Sixthly, CAMI-1 was designed more as a pilot- and safety- study than an outcome study. Seventhly, the optimal start point and intensity of CRP apheresis remains to be evaluated and optimized. Eighthly, our final statistical analysis is not typical for medical devices but is similar to studies with drugs, since a concentration-dependent effect could be assumed for the acute phase protein CRP: in order to relate CRP increase to the extent of myocardial damage we used the CRP gradient and propensity analysis during the course of the study.

However, dropouts' rates in the apheresis group were comparable to other trials using MRI analysis and mainly due to the CMR (e.g., panic reaction). Ninthly, based on the changed statistical analysis the power of this study was not sufficient for the analysis of MACEs. And lastly, observations in this study revealed that CRP apheresis might be most beneficial in patients exhibiting a high CRP gradient. This should be implemented in future studies.

### Outlook

With regard to safety, apheresis in general is a well-established technique. CRP apheresis in particular just necessitates the use of a specific CRP absorber. The CAMI-1 trial proves CRP apheresis as a safe treatment without relevant side effects in potentially unstable patients such as acute STEMI. Currently, CRP apheresis in acute STEMI is further evaluated in a recruiting registry and a controlled randomized multi-center trial is planned.

Furthermore, CAMI-1 includes CMRs performed between the 2nd and 9th day after STEMI. Because prognosis of STEMI strongly depends on LV function, the results of our study promise improved outcome after CRP apheresis as yet to prove in larger trials. Nevertheless, the prognostic value of CMR within the first 10 days after the onset of symptoms has been underscored in clinical studies and validated as an endpoint (52) and the statistical analysis of the relatively small number of patients of the CAMI-1 trial already indicates a significant improvement for the treated group.

#### Clinical Perspectives

The CAMI-1 study introduces a new clinical tool: CRP apheresis may prove its usefulness in the treatment of inflammatory diseases such as other subtypes of acute coronary syndromes (53) and stroke (54, 55), in which CRP might be causally involved.

### Conclusion

The results of CAMI-1 underscore the correlation of CRP load and myocardial damage in acute STEMI, the effectiveness and benefits of CRP elimination by CRP apheresis and the safety and feasibility of the latter. For the first time it is shown that specific CRP depletion in acute STEMI seems to result in clinical benefits and offers a new therapeutic approach as acute treatment of inflammation in myocardial infarction.

## Data Availability Statement

The raw data supporting the conclusions of this article will be made available by the authors, without undue reservation.

## Ethics Statement

The studies involving human participants were reviewed and approved by Medical Association Schleswig-Holstein, Germany No 042/15 (I). The patients/participants provided their written informed consent to participate in this study.

## Author Contributions

WR, AS, and CG: designed the research study. WR, JT, FH, CP, HD, HI, SM, PN, CB, and CG: acquired data. HS, SK, and AS: analyzed data. WR, JT, FH, AS, SK, HS, and CG: wrote the manuscript. All authors: contributed to the article and approved the submitted version.

## Conflict of Interest

AS was a founder and is shareholder and managing director of Pentracor GmbH. The remaining authors declare that the research was conducted in the absence of any commercial or financial relationships that could be construed as a potential conflict of interest.
