# Supplementary material for: C-Reactive Protein Apheresis as Anti-inflammatory Therapy in Acute Myocardial Infarction: Results of the CAMI-1 Study
Source: Front Cardiovasc Med. 2021 Mar 10;8:591714. doi: 10.3389/fcvm.2021.591714 (PMC7988098; doi:10.3389/fcvm.2021.591714)
Supplement: Supplementary file 1 [file Data_Sheet_1.pdf]

Supplementary Material

Table of Contents

List of Investigators ………………………………………………………………… 2

Supplementary Methods ………………………………………………………………. 3

Supplementary Results ……………………………………………………………... 4

Supplementary Discussion …………………………………………………………….. 7

Supplementary References …………………………………………………………….. 9

Study protocol CAMI-1………………………………………………………………… 11

Patient CRP kinetics…………………………………………………………………. 18

List of Investigators

Listed in alphabetical order.

Andrik Aschoff[1], Markus Böck[2], Christian Butter[3], Harald Darius[4], Silvius Frimmel[5], Andreas Führer[5], Christoph Dietrich Garlichs[6], Matthias Graf[7], Franz Heigl[8], Reinhard Hettich[8], Michael Hinz[5], Jan Horstkotte[1], Christian Hugo[9], Hüseyin Ince[5], Sebastian Kelle[10a,10b,10c], Nina Kleinfeldt[5], Erdwine Klinker[2], Sebastian Koball[5], Tomas Lapinskas[10a], Stefanie Lehrke[11], Octavian Maniuc[12], Steffen Mitzner[5], Peter Nordbeck[12], Alper Öner[5], Christian Pfluecke[13], Wolfgang Ries[6], Stefan Schmiedel[6], Ahmed Sheriff[14], Horst Skarabis[15], Jan Torzewski[7], Myron Zaczkiewicz[7], Michael Zänker[3], Oliver Zimmermann[7]

[1]Department of Radiology, Kempten, Germany

[2]Transfusion Medicine, University Clinic Würzburg, Germany

[3]Immanuel Clinic Bernau, Heart Center Brandenburg, Germany

[4]Clinic for Cardiology, Angiology, Nephrology, Intensive Care Medicine, Vivantes Clinic Neukölln/Berlin, Germany

[5]Divisions of Cardiology and Nephrology, Department of Internal Medicine, University Medicine Rostock, Germany

[6]Medical Clinic, DIAKO Flensburg, Germany

[7]Cardiovascular Center Oberallgäu-Kempten, Germany

[8]Medical Care Center Kempten-Allgäu, Germany

[9]Department of Internal Medicine III, University Hospital Dresden, Germany

[10a]Department of Internal Medicine/Cardiology, German Heart Center Berlin, Germany

[10b]Charité University Medicine Berlin, Department of Internal Medicine/Cardiology, Campus Virchow, Germany

[10c]DZHK (German Centre for Cardiovascular Research), Partner Site Berlin, Germany

[11]Institute for Diagnostic/Interventional Radiology, DIAKO Flensburg, Germany

[12]Medical Clinic (Cardiology), University Clinic Würzburg, Germany

[13]Department for Internal Medicine/ Cardiology, Heart Center Dresden, Germany

[14]Department of Gastroenterology/Infectiology/ Rheumatology, Charité University Medicine Berlin, Germany

[15]Groß-Oesingen, Germany

Supplementary Methods

1. Statistics

The statistical methods used are comparatively simple, especially since the logarithm of the 'CRP gradient' as well as the residuals of the used linear models were pretty well normally distributed. As the methodological evaluations (bootstrapping, cross-validation, etc.) showed, results can be presented relatively easily in linear models and the adequate (non-)parametric statistical tests. The 'CRP gradient', i.e. the increase of the CRP in the first 32 h, predicts myocardial damage of STEMI (myocardial infarct size, LVEF, longitudinal and circumferential strain) and thus enables an 'ex-ante selection' of STEMI patients for CRP apheresis.

2. CRP apheresis

During apheresis flow of plasma and washing solutions were controlled by a software module for automatic plasma flow management (ADAsorb, Medicap Clinic GmbH, Germany). Plasma separation was achieved by centrifuge (SpectraOptia, TerumoBCT, USA; Com.Tec, Fresenius Kabi Deutschland GmbH, Germany) or filter system (ArtUniversal, FreseniusMedical Care AG, Germany).

3. CMR

Cardiovascular magnetic resonance (CMR) represents the gold standard measurement of left ventricular volumes, mass and function (Myerson et al., 2002). Its low intraobserver and interobserver variability reduces sample size in longitudinal studies (Bellenger et al., 2000; Grothues et al., 2002; Maceira et al., 2006). CMR is currently the noninvasive modality of choice for fibrosis and scar assessment (American College of Cardiology Foundation Task Force on Expert Consensus et al., 2010) and accurately quantifies longitudinal and circumferential strain (Mahfoud et al., 2014).

4. Image Analysis

Left ventricular function and mass: As part of CMR analysis, endocardial and epicardial contours were manually traced excluding the papillary muscles and trabeculations both in systole and diastole. Left ventricular (LV) end-diastolic (EDV) and end-systolic volumes (ESV) were quantified as well as LVEF and the LV myocardial volume and mass. Late enhancement was assessed primarily visually. For semiquantitative analysis of late enhancement, after tracing the endocardial and epicardial contours, the software automatically delineated areas of high signal intensity based on user-identified thresholds (using full-width-half-maximum method of SD of signal intensity of infarcted tissue) of standard deviations above that of the average normal myocardial signal intensity (remote myocardium was defined as that with preserved systolic function and absence of late gadolinium enhancement). The volume of myocardium exhibiting high signal intensity was then quantified and related to that of the slice volume. These volumes were multiplied by the specific gravity of the myocardium (1·05) to calculate the mass in grams.

Strain analysis included 2Ch, 3Ch and 4Ch cine images as well as 3 preselected slices from the LV short-axis stack to correspond to basal, mid-ventricular and apical levels. The endocardial contours were drawn on cine images with QMass version 8·1 and subsequently transferred to QStrain RE version 2·0, where endocardial and epicardial borders were detected throughout the whole cardiac cycle using a tissue tracking algorithm. The long-axis cine images were further used to compute the global myocardial longitudinal strain curve and, respectively, short-axis images were used to compute global circumferential strain curve. Peak global values of longitudinal and circumferential systolic strain were considered the maximal amplitude of the strain curves during the cardiac cycle. Image analysis of left ventricular function and mass was performed offline using commercially

available software (Medis Suite, version 3·1, Netherlands).

Supplementary Results

1. CMR parameters of CMR1 and CMR2 for control and apheresis patients

Supplementary Table 1 summarizes the results of both CMRs for both groups. Infarct size was significantly reduced in both groups at CMR2 compared to CMR1, while LVEF was significantly improved. Only in the apheresis group were both global strains (longitudinal and circumferential) significantly improved after 3 months. It should be noted that the area at risk was only measured for a proportion of patients and therefore the myocardial salvage index was only calculated for 42 patients (21/21; see table 3). It needs to be pointed out that CMR 2 has not been performed for all evaluated patients. Patient compliance 30 days after their leave from the hospital was not perfect and in addition, measuring infarct size by CMR is quite time-consuming and was not done accurately in a few cases. The smaller and non-significant difference in infarct sizes of both groups in CMR2 is explained by the different missing values. 5 control patients with missing values in CMR2 had extremely large infarct sizes in CMR1 (mean = 37%), significantly larger Gradients and AUC, while only 3 apheresis patients with missing values in CMR2 had significantly lower infarct values in CMR1 (mean = 14.5%). Unfortunately, the missing CMR2 data presents a huge bias for the analysis.

Supplementary Table 1: Overview of CMR Parameters between both CMRs and groups.

**Control**			
**Parameter**	**CMR1**	**CMR2**	**p-Value**
Time – days after onset	4.9 ± 2	96.0 ± 22.6	---
Infarct size - %	26.3 ± 13.4	18.0 ± 7.9	< 0.001
LVEF - %	51.8 ± 8.2	55.9 ± 7.8	0.002
Longitudinal strain	-20.4 ± 4.3	-21.8 ± 6.3	0.2
Circumferential strain	-25.3 ± 5.1	-26.6 ± 6.9	0.46
Area at risk - %	39.0 ± 16.7	---	---
Myocardial Salvage Index*	0.5 ± 0.19		---
**Apheresis**			
**Parameter**	**CMR1**	**CMR2**	**p-Value**
Time – days after onset	5.3 ± 2	91.9 ± 11.5	---
Infarct size - %	22.0 ± 11.4	18.6 ± 8.8	0.002
LVEF - %	51.6 ± 7.1	55.7 ± 8.0	0.004
Longitudinal strain	-19.7 ± 4.6	-22.4 ± 4.3	< 0.001
Circumferential strain	-25.3 ± 4.0	28.0 ± 5.7	0.001
Area at risk - %	35.4 ± 12.7	---	---
Myocardial Salvage Index*	0.4 ± 0.25		---

Data are presented as mean ± SD.
*Myocardial salvage index is calculated as follows: (Area at risk 1 – Infarct size 2)/Area at risk 1
P-values were calculated using either a paired Welch-t-Test (normally distributed data) or a paired Wilcoxon rank sum test with continuity correction (at least one group not normally distributed).

2. Relationship Area under the Curve (CRP) and gradient (CRP)

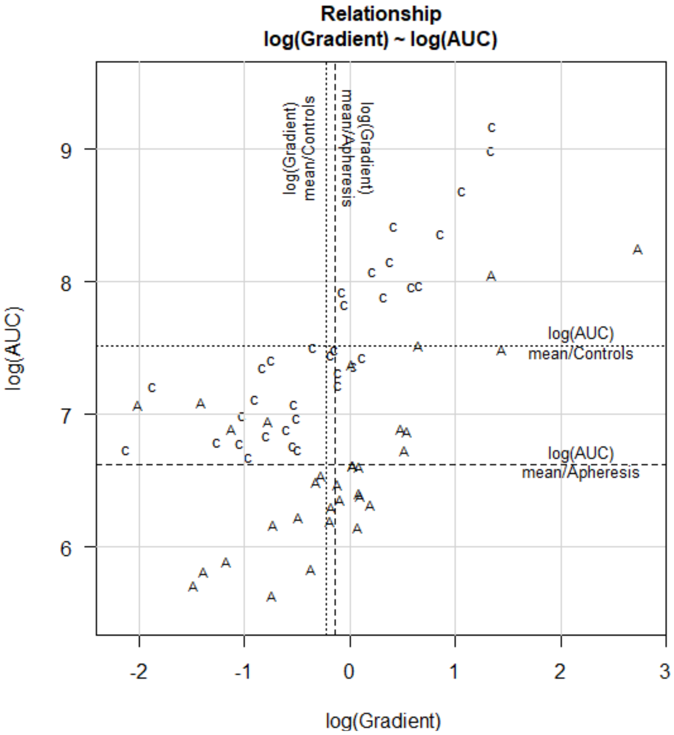

Supplementary Figure 1: Relationship $\log_e$(AUC) and $\log_e$(gradient).
Graph plots the area under the curve (AUC CRP) in regard to the $\log_e$(gradient) for both control (C) and apheresis (A) patients. Mean for both parameters and both groups is marked in dashed lines. Importantly, mean CRP gradient does not differ between both groups, whereas mean AUC does (see also Figure 2). Thus, there was no bias in CRP kinetics between the two groups.

3. Propensity correction for parameters of both CMRs

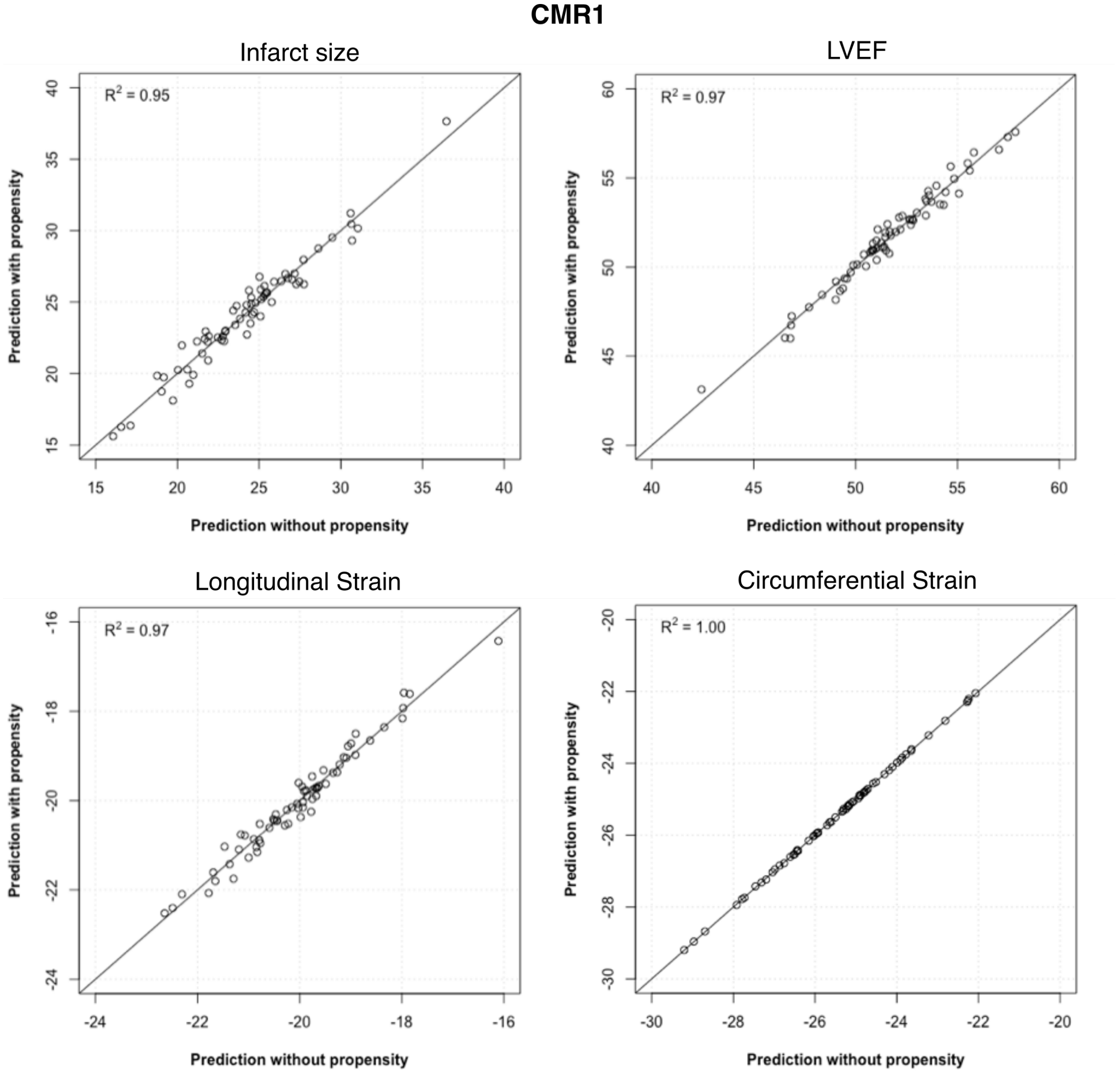

Supplementary Figure 2: Influence of propensity correction on CMR1 parameters.
The 4 plots show the correlation of non-adjusted linear models and propensity-corrected linear models for infarct size, LVEF, longitudinal strain and circumferential strain. Propensity adjustment was calculated based on the variables age, location of infarct and time to stent. None of the models differed significantly. Same analysis for parameters of CMR2 is shown in Supplementary Figure 3.

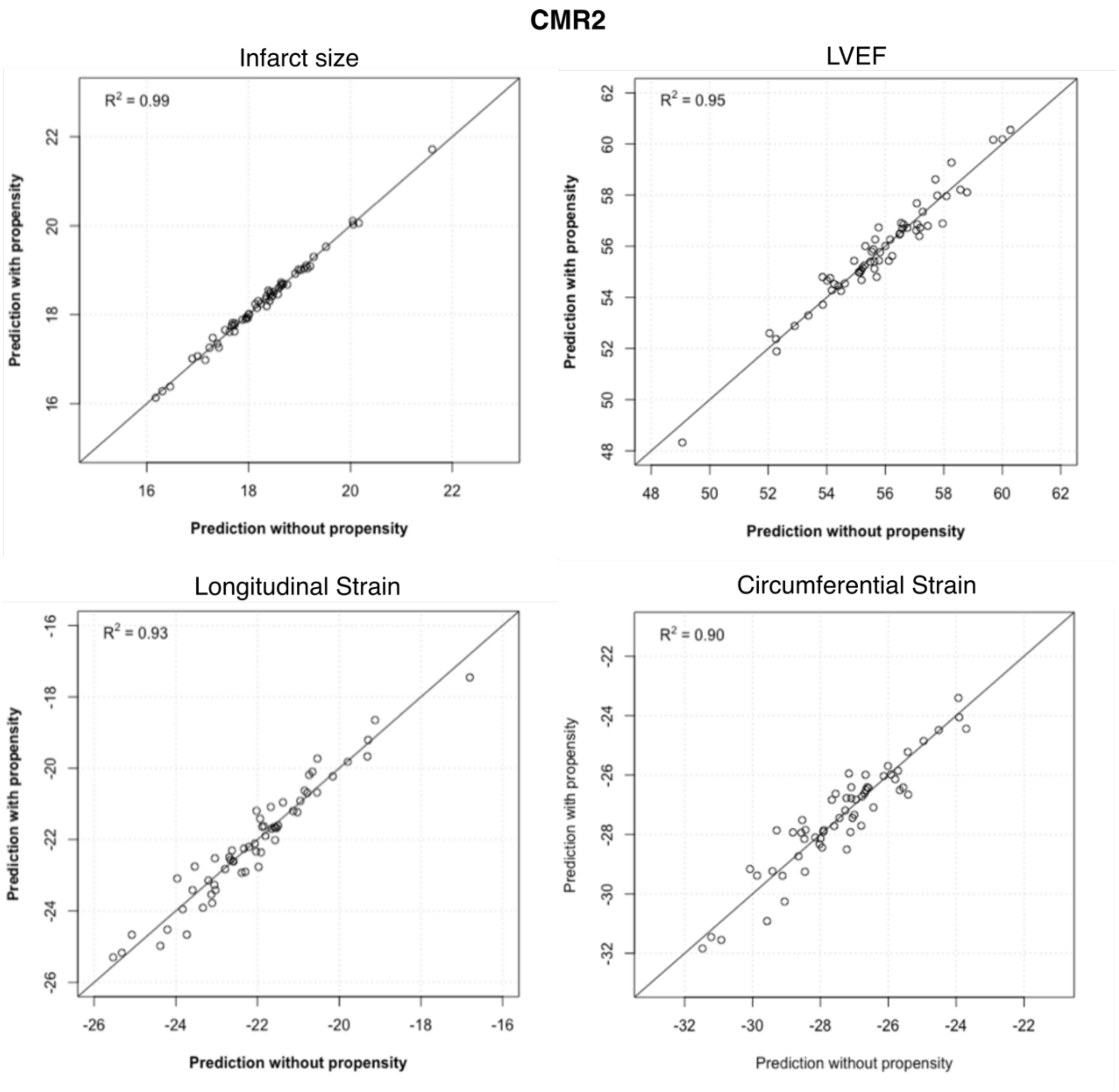

Supplementary Figure 3: Influence of propensity correction on CMR2 parameters.
The 4 plots show the correlation of non-adjusted linear models and propensity-corrected linear models for infarct size, LVEF, longitudinal strain and circumferential strain. Propensity adjustment was calculated based on the variables age, location of infarct and time to stent. None of the models differed significantly.

Supplementary Discussion

Pathophysiologically, ischemic myocardium in the acute phase consists of a primarily edematous infarction zone with energy and oxygen deprived myocardial cells switching their metabolism to glycolysis instead of using the respiratory chain. Due to lack of energy, these cells are unable to repair their membranes. The integrity of the cell membranes is threatened by oxidation processes mediated by another, wound-induced acute phase protein, the secretory phospholipase A2 type IIa (sPLA2 IIa) (Hack and Niessen, 2002; Nijmeijer et al., 2002; Nijmeijer et al., 2003; Krijnen et al., 2006). The amount of sPLA2 IIa increases 2h before the CRP increase. This hardly recognized acute phase protein catabolises phosphatidylcholine (PC) to lysophosphatidylcholine (LPC)(Kudo and Murakami, 2002), the natural ligand of CRP in cell membranes. LPC allows the binding of systemically increased CRP to local hypoxic cells in the area of inflammation surrounding infarcted area thus increasing myocardial infarct size. CRP binding to LPC induces a conformational change of

the CRP pentamer enabling the binding of complement factor C1q to CRP and sequential activation of the complement components up to C3 and C4 (Mevorach et al., 1998; Gershov et al., 2000). CRP and complement only mark myocardial cells as dead or dying for later disposal by phagocytes. The exact mechanism of CRP-dependent myocardial cell death in cells that were initially vital in the first 2 days after myocardial infarction still has to be clarified. According to current knowledge, phagocytes recognize CRP- and complement labelled myocardial cells, categorize them as dead and engulf them (Supplementary Figure 3).

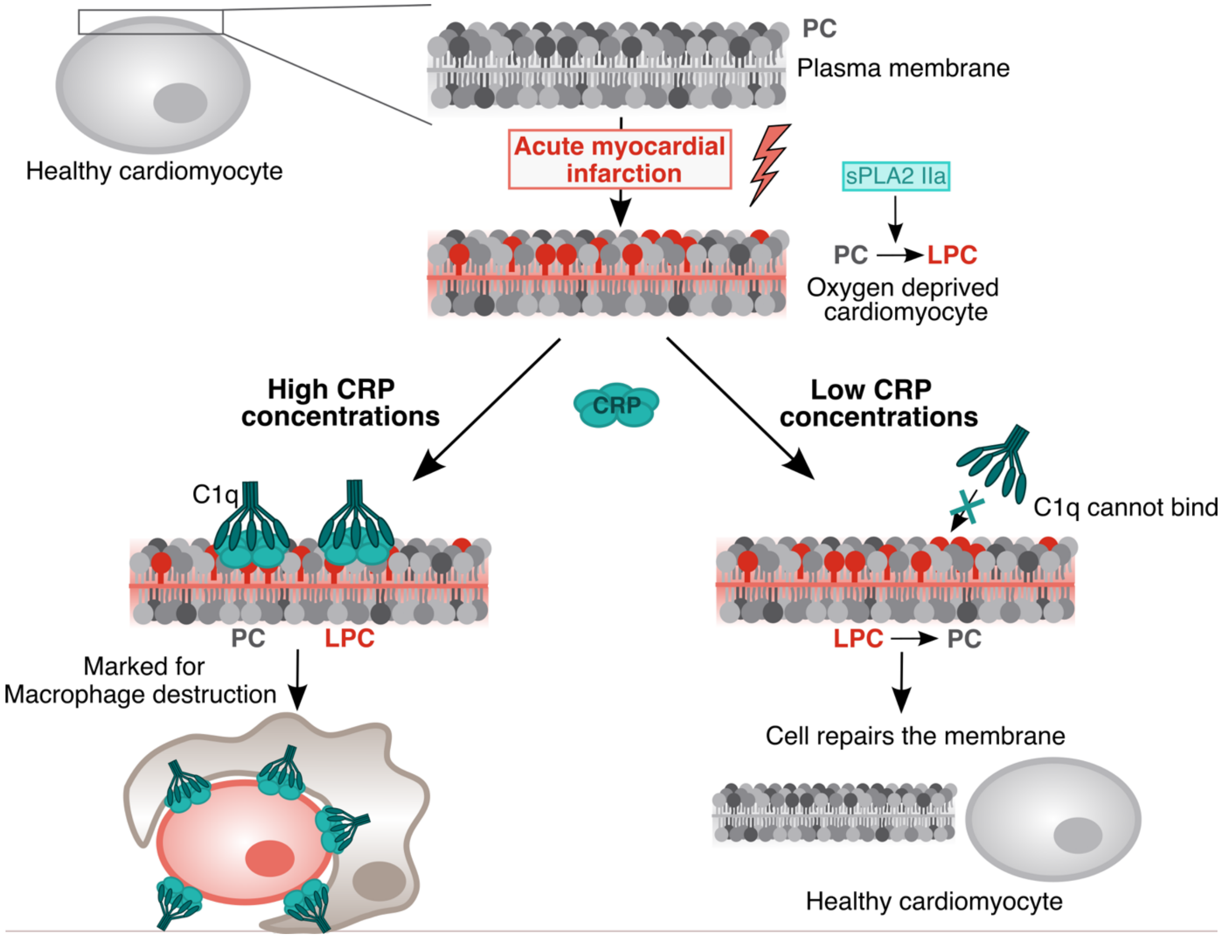

Supplementary Figure 2: Molecular pathomechanism of CRP at the cell membrane.
Acute myocardial infarction leads to oxygen deprivation in cardiomyocytes, which either undergo immediate apoptosis/necrosis or are energy deprived. This stress induces changes in the molecular organization of the plasma membrane bilayer: PC is converted into LPC by action of sPLA2 IIa. CRP can subsequently bind to LPC on energy deprived cells and recruit complement factors leading to opsonization and destruction of cells. Without CRP or in situations with low CRP concentrations (e.g. due to CRP apheresis), destruction of energy deprived cells is not facilitated by complement and cells have enough time to repair the molecular changes and become vital cardiomyocytes again, leading to an overall reduced tissue damage.
PC Phosphatidylcholine; LPC Lysophosphatidylcholine; CRP C-reactive protein; sPLA2 IIa secretory Phospholipase A2 IIa; C1q Complement component 1q.

CRP apheresis after AMI eliminates systemic CRP in the patient, counteracts ongoing CRP production by continuous removal of CRP and thus gives myocardial cells time to repair their membranes. CRP apheresis reduces the probability that CRP binds to lysophosphatidylcholine, thus

reducing the number of cells affected by CRP. It is conceivable that the more intensively CRP apheresis is performed, the more myocardial cells are salvaged from the 'risk zone' of the myocardial infarction.

Supplementary References

American College of Cardiology Foundation Task Force on Expert Consensus, D., Hundley, W.G., Bluemke, D.A., Finn, J.P., Flamm, S.D., Fogel, M.A., et al. (2010). ACCF/ACR/AHA/NASCI/SCMR 2010 expert consensus document on cardiovascular magnetic resonance: a report of the American College of Cardiology Foundation Task Force on Expert Consensus Documents. *J Am Coll Cardiol* 55(23)**,** 2614-2662. doi: 10.1016/j.jacc.2009.11.011.

Bellenger, N.G., Davies, L.C., Francis, J.M., Coats, A.J., and Pennell, D.J. (2000). Reduction in sample size for studies of remodeling in heart failure by the use of cardiovascular magnetic resonance. *J Cardiovasc Magn Reson* 2(4)**,** 271-278.

Gershov, D., Kim, S., Brot, N., and Elkon, K.B. (2000). C-Reactive protein binds to apoptotic cells, protects the cells from assembly of the terminal complement components, and sustains an antiinflammatory innate immune response: implications for systemic autoimmunity. *J Exp Med* 192(9)**,** 1353-1364.

Grothues, F., Smith, G.C., Moon, J.C., Bellenger, N.G., Collins, P., Klein, H.U., et al. (2002). Comparison of interstudy reproducibility of cardiovascular magnetic resonance with two-dimensional echocardiography in normal subjects and in patients with heart failure or left ventricular hypertrophy. *Am J Cardiol* 90(1)**,** 29-34.

Hack, C.E., and Niessen, H.W. (2002). Cardiovascular secrets of secretory phospholipase A(2). *Eur J Clin Invest* 32(6)**,** 381-382.

Krijnen, P.A., Meischl, C., Nijmeijer, R., Visser, C.A., Hack, C.E., and Niessen, H.W. (2006). Inhibition of sPLA2-IIA, C-reactive protein or complement: new therapy for patients with acute myocardial infarction? *Cardiovasc Hematol Disord Drug Targets* 6(2)**,** 113-123.

Kudo, I., and Murakami, M. (2002). Phospholipase A2 enzymes. *Prostaglandins Other Lipid Mediat* 68-69**,** 3-58.

Maceira, A.M., Prasad, S.K., Khan, M., and Pennell, D.J. (2006). Normalized left ventricular systolic and diastolic function by steady state free precession cardiovascular magnetic resonance. *J Cardiovasc Magn Reson* 8(3)**,** 417-426.

Mahfoud, F., Urban, D., Teller, D., Linz, D., Stawowy, P., Hassel, J.H., et al. (2014). Effect of renal denervation on left ventricular mass and function in patients with resistant hypertension: data from a multi-centre cardiovascular magnetic resonance imaging trial. *Eur Heart J* 35(33)**,** 2224-2231b. doi: 10.1093/eurheartj/ehu093.

Mevorach, D., Mascarenhas, J.O., Gershov, D., and Elkon, K.B. (1998). Complement-dependent clearance of apoptotic cells by human macrophages. *J Exp Med* 188(12)**,** 2313-2320.

Myerson, S.G., Bellenger, N.G., and Pennell, D.J. (2002). Assessment of left ventricular mass by cardiovascular magnetic resonance. *Hypertension* 39(3)**,** 750-755.

Nijmeijer, R., Lagrand, W.K., Baidoshvili, A., Lubbers, Y.T., Hermens, W.T., Meijer, C.J., et al. (2002). Secretory type II phospholipase A(2) binds to ischemic myocardium during myocardial infarction in humans. *Cardiovasc Res* 53(1)**,** 138-146.

Nijmeijer, R., Willemsen, M., Meijer, C.J., Visser, C.A., Verheijen, R.H., Gottlieb, R.A., et al. (2003). Type II secretory phospholipase A2 binds to ischemic flip-flopped cardiomyocytes and subsequently induces cell death. *Am J Physiol Heart Circ Physiol* 285(5)**,** H2218-2224. doi: 10.1152/ajpheart.00887.2002.

Study protocol CAMI1

Title of the study	**Selective depletion of C-reactive protein by therapeutic apheresis (CRP-apheresis) in acute myocardial infarction**
Short title	**CAMI1-Study on CRP-apheresis in acute myocardial infarction**
Sponsor	Pentracor GmbH<br>Neuendorfstr. 23 b/d, 16761 Hennigsdorf<br>Tel.: +49 3302 209449-35, Fax: +49 3302 209449-99<br>E-Mail: info@pentracor.de
Principle Investigator (PI)	Dr. med. Wolfgang Ries<br>Diakonissenkrankenhaus Flensburg<br>Medizinische Klinik – Innere Medizin<br>Knuthstraße 1, 24939 Flensburg<br>Tel.: +49 461 812 1331, Fax: +49 461 812 1386<br>E-Mail: rieswo@diako.de
The purpose of the study and its objectives	In patients with acute myocardial infarction, the rapid restoration of coronary perfusion by percutaneous coronary intervention (PCI) is the established strategy for reducing the size of the infarct and improving its consequences (outcome).<br>Subject of the study is the reduction of plasma concentration of C-reactive protein (CRP) by CRP apheresis in patients with ST-segment elevation myocardial infarction (STEMI) in addition to PCI. The aim of the study is to analyse the effect of CRP reduction on myocardial damage. A possible protective effect of CRP apheresis on the development of such damage will be determined by the functional parameters 'size of infarct scar' and 'left ventricular ejection fraction (LVEF)' by cardiac magnetic resonance imaging (MRI).<br><br>**Background**<br>The C-reactive protein is an acute-phase protein. These are proteins whose concentrations increase in the blood during inflammatory (infectious and non-infectious) diseases. CRP is established as a sensitive, reliable and early indicator of inflammatory processes. After an acute-phase stimulus CRP-levels can rise up to more than 1.000 times.<br>Human CRP binds to lyso-phosphatidylcholine with high affinity, which is accessible in the outer membrane of damaged cells. Therefore, it can only interact with damaged cell membranes but not with healthy cells. Such an injury to the cell membranes is formed after a myocardial infarction and reperfusion of the coronary arteries in the infarction zone (so-called area at risk) by the reoxygenation process.<br>The binding of CRP to lyso-phosphatidylcholine on damaged membranes leads to the activation of complement and binding of macrophages.<br>Already 6 hours after the onset of symptoms of an acute myocardial infarction, increased CRP-Levels can be detected in the serum with a peak after approximately 48 hours [1].

	In 1982, a relation between high CRP-levels after acute myocardial infarction and negative effects during follow up were described for the first time [2]. An increased CRP-concentration with an increased incidence of cardiac complications could be detected since then in many clinical trials [3-13].<br>It is assumed that the reason for the injurious effect of the C-reactive protein is its role as a mediator of inflammation. CRP triggers the destruction of heart muscle tissue in conjunction with complement and negatively affects the regeneration of the ischemic tissue [14-16].<br>In addition to its diagnostic and prognostic use as a marker of inflammatory reactions, CRP itself could be involved in the pathogenesis of myocardial infarction.<br><br>Studies on an animal model (rat) suggest that the CRP response not only reflects tissue damage, but also contributes significantly to the severity of ischemic myocardial damage. In these studies it was shown that CRP increases myocardial infarct areas after acute occlusion via a complement dependent mechanism [17]. By adding a low-molecular CRP inhibitor, the enlargement of the infarct areas and the cardiac dysfunction could be interrupted in the course of the disease [18]. From this it can be deduced that a significant part of the final size of myocardial infarction after acute coronary occlusion is determined by complement mediated inflammation and that the CRP is responsible for at least part of this complement activation.<br>Lowering the CRP level after acute myocardial infarction thus appears to be a promising new therapeutic approach to cardioprotection.<br><br>CRP apheresis is a technique of therapeutic apheresis. The term therapeutic apheresis refers to general medical procedures whose therapeutic effect is based on the elimination of components of the blood to which a pathogenic function is attributed within the framework of disease processes. Elimination takes place in adsorbers outside the body in an extracorporeal circuit. To remove the pathogenic substances, plasma is separated from the blood (circulation) and passed over the adsorber. The purified plasma is then reunited with the solid blood components and returned to the patient.<br>Therapeutic apheresis represents an effective and safe therapeutic approach for numerous clinical pictures. Treatment options are available in a wide range of medical disciplines. Apheresis can also be used to treat diseases for which conventional therapy concepts are no longer effective.<br><br>**Hypothesis**<br>- In patients with acute myocardial infarction whose primary therapy has been carried out according to European standards [19] and whose CRP concentration is subsequently reduced by CRP apheresis, tissue damage of the heart (reperfusion damage) and thus the size of the myocardial infarction can be reduced. This can counteract the occurrence of secondary complications of acute myocardial infarction, in

	particular the development of heart failure. Acute complications of myocardial infarction can also be reduced, such as ventricular aneurysm (with an increased risk of stroke) or myocardial rupture, which can occur particularly after major myocardial infarctions and are associated with highly elevated CRP.<br>- The CRP-apheresis can be carried out safely after acute myocardial infarction.<br><br>Primary Endpoint:<br>• Infarction size, determined by MRI of the heart 5 ± 3 days as well as 12 ± 2 weeks after the infarction.<br>Secondary Endpoint:<br>• Incidence of expected and unexpected adverse effects of the CRP-apheresis<br>• LVEF (left ventricular ejection fraction), determined by MRI of the heart 5 ± 3 days as well as 12 ± 2 weeks after the infarction.<br>• Major adverse cardiac events (MACE) 6 and 12 months after the infarction. MACEs are defined as follows: death for any reason, non-fatal re-infarction or stroke, unstable angina pectoris, congestive heart failure followed by hospital treatment and coronary revascularization (percutaneous coronary angioplasty or coronary artery bypass). The time until the first event is used as the end point.
Key results of pre-clinical tests	Preclinical studies on the depletion of the C-reactive protein were successfully performed on an animal model (pig). In these studies, myocardial infarction was triggered by temporary occlusion (60 minutes) of the D2 branch of the ramus Interventricularis anterior (RIVA) using a balloon catheter.<br>The vascular occlusion led to infarcts with sizes of approximately 10% of the volume of the left ventricle, and an increase of the CRP-level in the plasma.<br>Due to the subsequent CRP-apheresis, a significant reduction of the CRP-levels in the treated animals could be achieved [20]. In further studies with this animal model it could be shown that the infarct size was reduced by 30% and the ejection fraction of the heart could be significantly increased [21]. The results of these investigations show a safe, efficient and selective reduction of C-reactive protein plasma levels. Adverse effects (including negative influences on important plasma components) were not observed in the test animals.
Description of the planned measures / methods	The therapy of the acute myocardial infarction (AMI) is carried out in accordance with the guidelines for Percutaneous Coronary Intervention (PCI) (including ESC-guideline 'myocardial revascularization', in 2014) [19].<br><br>CRP apheresis is carried out as an additional therapeutic measure.<br><br>45 patients will receive 2 treatments with an interval of 24 ± 12 hours (after the start of the previous treatment). The first treatment starts 10 - 36 hours after the onset of symptoms of myocardial infarction. If

	approximately 6 hours after the end of the second treatment the CRP-concentration rises to values above 30 mg/L, a third treatment will be performed.<br><br>In each treatment up to 6000 ml of plasma will be processed, preferentially in 6 cycles (change of loading and regeneration of the adsorber) of 1000 ml. The duration of one treatment will be approx. 4 - 6 h.<br><br>45 patients of the control group do not receive CRP-apheresis after PCI.<br><br>All patients receive two MRIs of the heart to evaluate the size of the infarction area and the LVEF:<br>1st MRI: 5 ± 3 days after the infarction,<br>2nd MRI: 12 ± 2 weeks after the infarction.<br>CRP-levels are determined in 12 hours intervals (up to 96 hrs.) starting from the onset of symptoms.<br>To monitor the apheresis, approximately 1.0 ml of plasma will be taken before and after each treatment from the extracorporeal circulation to determine the concentration of different plasma parameters like total protein and fibrinogen.
Number, age and sex of the persons concerned	Approx. 90 patients (incl. 45 controls), both sexes, 18-79 years of age.
Study design	Multicenter, non-randomized, controlled pilot clinical trial.<br>Cardiac MRIs will undergo blinded evaluation by a core-lab.
Inclusion / Exclusion criteria	Inclusion criteria:<br>• STEMI as defined by the guidelines of the European Society of Cardiology (ESC) for the treatment of AMI in patients with ST-segment elevation.<br>• TIMI grade III after PCI (stent implantation)<br>• Killip-Class ≤ II<br>• 2-12 h from onset of symptoms until coronary reperfusion<br>Exclusion criteria:<br>• Age < 18 ≥ 80 years<br>• Previous myocardial infarction<br>• Acute infectious disease (body temperature (auricular, sublingual) > 38.0 °C)<br>• Systolic blood pressure < 100 mmHg<br>• Known hypersensitivity to therapeutic apheresis<br>• Cardiogenic shock<br>• Renal insufficiency<br>• Previous coronary artery bypass surgery<br>• Contraindication for MRI (e.g. non-MRI-capable implants,

	claustrophobia)<br>• Malignant or chronic inflammatory disease<br>• Pregnancy or lactation period<br>• Limited possibility to join the follow-up examination (e.g., patient lives abroad)<br>• Participation in other interventional trial
Target parameter	<u>Clinical Efficacy of CRP apheresis:</u><br>• Infarct size<br>• Left ventricular ejection fraction (LVEF)<br>• Major Adverse Cardiac Events (MACE)<br>Efficacy is measured by the size of the infarct area, the level of ejection from the left ventricle, and the time from treatment to the appearance of MACEs.<br><u>Safety and tolerability of CRP apheresis:</u><br>To assess safety, continuous monitoring of the vital parameters blood pressure, heart rate and oxygen saturation as well as the general well-being of the study participants during the treatments is carried out.<br>In order to identify undesirable events that occur with delay, a state of health survey is carried out one day after each treatment.
Termination criteria	<u>For the study participant:</u><br>• Withdrawal of consent<br>• Occurrence of a new concomitant disease that precludes further study participation<br>• Increased occurrence of unforeseeable serious adverse reactions which are life-threatening<br>• Subsequent determination of the violation of the inclusion criteria<br>• Subsequent occurrence of an exclusion criterion<br><u>For the study:</u><br>• Decision of the study management in the case of unjustifiable risks, taking into account the risk-benefit balance (the risk-benefit ratio for the study participants changes significantly).<br>• New (scientific) findings arise during the course of the clinical trial which may jeopardise the safety of the study participants (positive benefit-risk balance no longer given)<br>• The clinical trial proves to be impracticable.
Statistical methods	The evaluation of the results will be carried out as matched-pairs analysis.<br>The two matching partners must be in the same range of values in the following three criteria:<br>1. Localization of the infarction: anterior wall // posterior wall<br>2. Patient's age: <70 years // ≥ 70 years<br>3. Time from onset of symptoms to coronary recanalization (stenting): 120-240 min // 241-360 min // 361-720 Min.

	For the determination of the necessary sample sizes, the following general assumptions were made: significance level: 5%, statistical power: 80%.<br>Phenotype for main hypothesis: Reduction of size of infarct area.<br>Significance and power were determined after extensive discussion and taking into account preliminary animal studies as well as literature data on mean value and variability of the target phenotype. The hypothesis testing will be carried out by means of t-tests for independent samples with two-sided questions.<br>For the standard deviation a value of 15 is considered realistic, for the group difference a value of 15 is expected.<br>Based on these assumptions, 34 patients per group are to be examined. A drop-out of individual patients must be expected, therefore an inclusion of approx. 45 patients per group (altogether approx. 90 patients) is recommended.
Possible risks, contraindications, measures to be taken in the case of possible incidents	Adverse effects of the adsorber PentraSorb® CRP are not known. However, CRP apheresis can have the same general side effects as other extracorporeal procedures. The following side effects are described for comparable adsorbers with agarose/sepharose matrices:<br>Blood pressure drop, blood pressure increase, tachycardia, bradycardia, cardiac arrhythmia, paresthesia, headache, nausea and vomiting, dizziness, chills, sweating, fever, pain, tremor, cramps, breathing difficulties, edema.<br>Allergic and anaphylactoid reactions cannot be ruled out.<br>In the course of the treatment minor plasma losses occur.<br>The PentraSorb® CRP adsorber is not to be used in cases of known hypersensitivity to therapeutic apheresis, in patients in whom suitable anticoagulation is not possible, or in clinical situations that do not allow for temporary volume changes.<br>If incidents occur, an adequate response is immediately possible as the study participants are under medical supervision during treatment.
Risk-benefit analysis	Therapeutic aphereses represent an effective and safe therapeutic approach for numerous clinical indications. Treatment options are offered in a wide range of medical disciplines. Apheresis can also be used to treat diseases for which conventional therapy approaches are no longer effective. The procedures are considered to cause very few side effects.<br>Of the undesirable effects described in the literature within the framework of therapeutic aphereses, only a very small proportion is causally attributed to the applied adsorbers. These specific side effects include non-specific binding and activation of plasma proteins.<br>A much larger group of undesirable effects, on the other hand, is largely independent of the apheresis systems or adsorber being used and is causally attributed to the extracorporeal circulation, the venous access or the anticoagulation.<br>From these data it can be deduced that CRP apheresis can also be performed safely and that potential side effects will not differ in their probability of occurrence and severity from those of other apheresis systems or comparable adsorbers.<br>According to the results of the animal experiments carried out and the hemocompatibility studies, an additional risk for patients by the newly developed ligand of the adsorber PentraSorb® CRP can also be ruled out with great probability.

	For the risk-benefit assessment of the proposed clinical trial, it can thus be summarized that the great potential benefit of improving cardiac performance through CRP apheresis is offset by a very low risk for the patient.
Follow-up care	Subjects will be treated after CRP apheresis according to the needs of their underlying conditions.
GCP-compliance	This clinical trial is conducted in accordance with the DIN EN ISO 14155:2012-01 standard.

References:

1. Pepys, M.B. and G.M. Hirschfield, *C-reactive protein: a critical update.* J Clin Invest, 2003. **111**(12): p. 1805-1812.
2. de Beer, F.C., et al., *Measurement of serum C-reactive protein concentration in myocardial ischaemia and infarction.* Br Heart J, 1982. **47**(3): p. 239-243.
3. Kinjo, K., et al., *Impact of high-sensitivity C-reactive protein on predicting long-term mortality of acute myocardial infarction.* Am J Cardiol, 2003. **91**(8): p. 931-935.
4. Suleiman, M., et al., *Admission C-reactive protein levels and 30-day mortality in patients with acute myocardial infarction.* Am J Med, 2003. **115**(9): p. 695-701.
5. Suleiman, M., et al., *Early inflammation and risk of long-term development of heart failure and mortality in survivors of acute myocardial infarction predictive role of C-reactive protein.* J Am Coll Cardiol, 2006. **47**(5): p. 962-968.
6. Pietilä, K.O., et al., *Serum C-reactive protein concentration in acute myocardial infarction and its relationship to mortality during 24 months of follow-up in patients under thrombolytic treatment.* Eur Heart J, 1996. **17**(9): p. 1345-1349.
7. Anzai, T., et al., *C-reactive protein as a predictor of infarct expansion and cardiac rupture after a first Q-wave acute myocardial infarction.* Circulation, 1997. **96**(3): p. 778-784.
8. Bursi, F., et al., *C-reactive protein and heart failure after myocardial infarction in the community.* Am J Med, 2007. **120**(7): p. 616-622.
9. Kavsak, P.A., et al., *Elevated C-reactive protein in acute coronary syndrome presentation is an independent predictor of long-term mortality and heart failure.* Clin Biochem, 2007. **40**(5-6): p. 326-329.
10. Ueda, S., et al., *C-reactive protein as a predictor of cardiac rupture after acute myocardial infarction.* Am Heart J, 1996. **131**(5): p. 857-860.
11. Pietila, K., et al., *Serum C-reactive protein and infarct size in myocardial infarct patients with a closed versus an open infarct-related coronary artery after thrombolytic therapy.* Eur Heart J, 1993. **14**(7): p. 915-9.
12. Dimitrijević, O., et al., *Serial measurements of C-reactive protein after acute myocardial infarction in predicting one-year outcome.* Int Heart J, 2006. **47**(6): p. 833-842.
13. Ohlmann, P., et al., *Prognostic value of C-reactive protein and cardiac troponin I in primary percutaneous interventions for ST-elevation myocardial infarction.* Am Heart J, 2006. **152**(6): p. 1161-1167.
14. Beranek, J.T., *C-reactive protein and complement in myocardial infarction and postinfarction heart failure.* Eur Heart J, 1997. **18**(11): p. 1834-1836.
15. Hack, C.E., et al., *A role for secretory phospholipase A2 and C-reactive protein in the removal of injured cells.* Immunol Today, 1997. **18**(3): p. 111-115.
16. Kitsis, R.N. and I. Jialal, *Limiting myocardial damage during acute myocardial infarction by inhibiting C-reactive protein.* N Engl J Med, 2006. **355**(5): p. 513-515.
17. Griselli, M., et al., *C-reactive protein and complement are important mediators of tissue damage in acute myocardial infarction.* J Exp Med, 1999. **190**(12): p. 1733-1740.
18. Pepys, M.B., et al., *Targeting C-reactive protein for the treatment of cardiovascular disease.* Nature, 2006. **440**(7088): p. 1217-1221.
19. Windecker, S., et al., *2014 ESC/EACTS Guidelines on myocardial revascularization: The Task Force on Myocardial Revascularization of the European Society of Cardiology (ESC) and the European Association for Cardio-Thoracic Surgery (EACTS) Developed with the special contribution of the European Association of Percutaneous Cardiovascular Interventions (EAPCI).* EuroIntervention, 2014.
20. Slagman, A.C., et al., *Specific removal of C-reactive protein by apheresis in a porcine cardiac infarction model.* Blood Purif, 2011. **31**(1-3): p. 9-17.
21. Sheriff, A., et al., *Selective apheresis of C-reactive protein: a new therapeutic option in myocardial infarction?* J Clin Apher, 2015. **30**(1): p. 15-21.

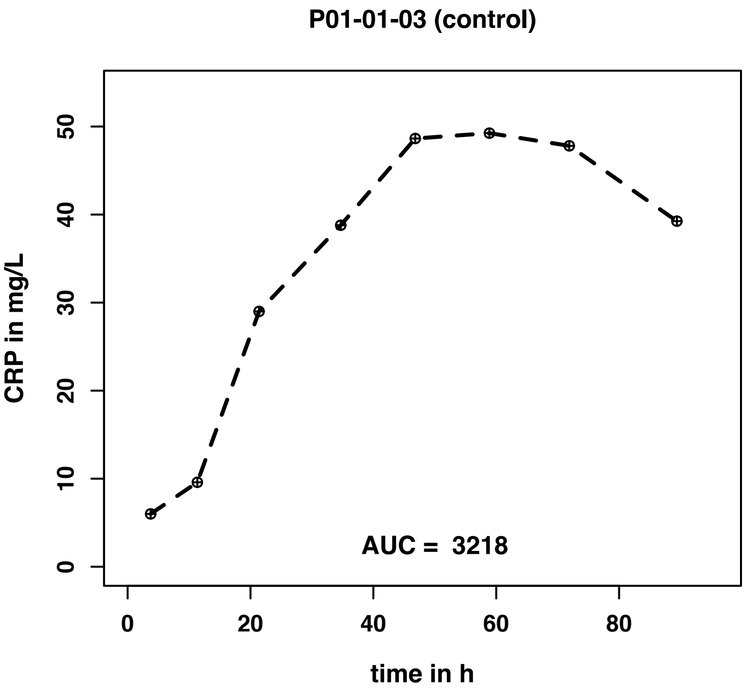

P01-01-03 (control)
CRP in mg/L
time in h
AUC = 3218

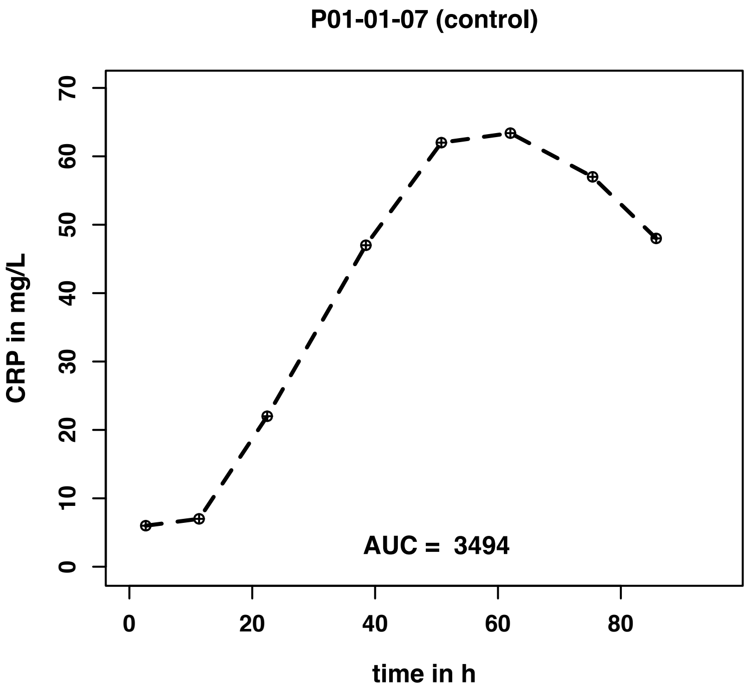

P01-01-07 (control)
CRP in mg/L
time in h
AUC = 3494

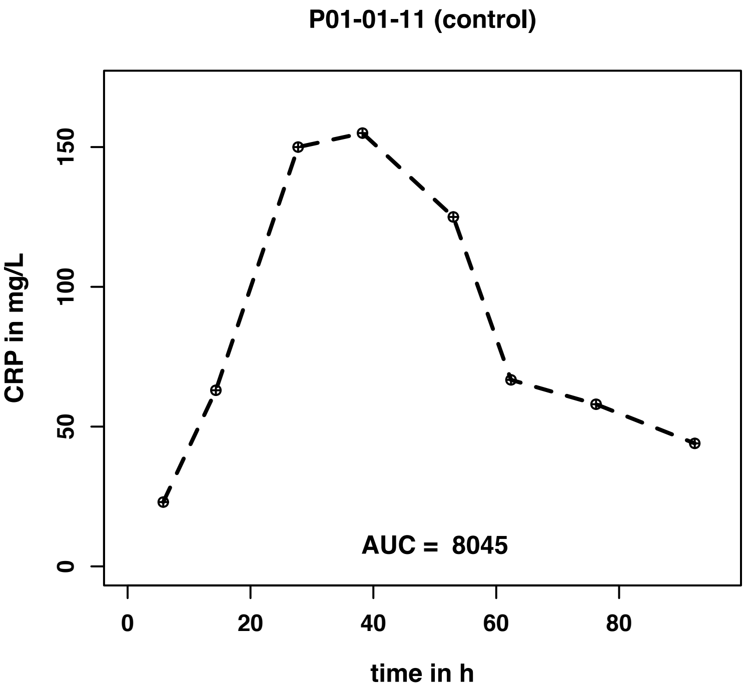

P01-01-11 (control)
CRP in mg/L
time in h
AUC = 8045

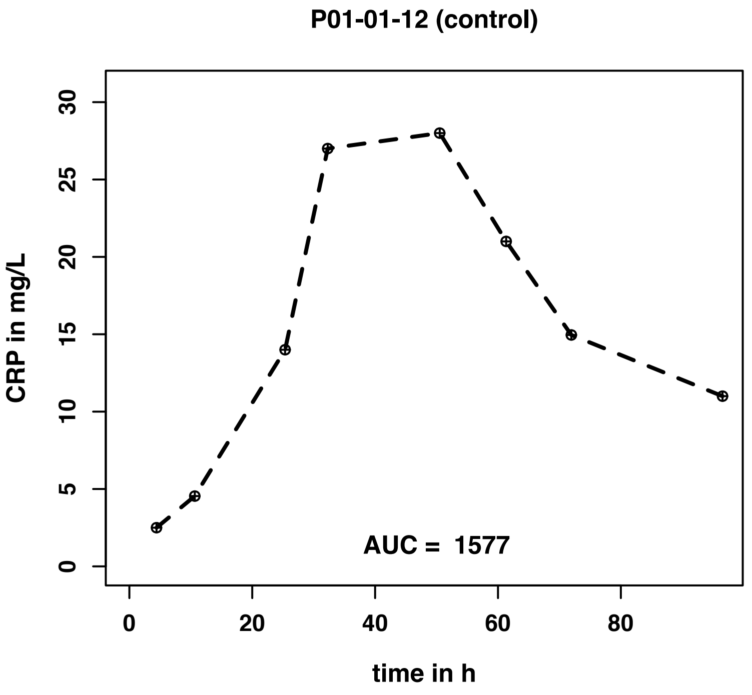

P01-01-12 (control)
CRP in mg/L
time in h
AUC = 1577

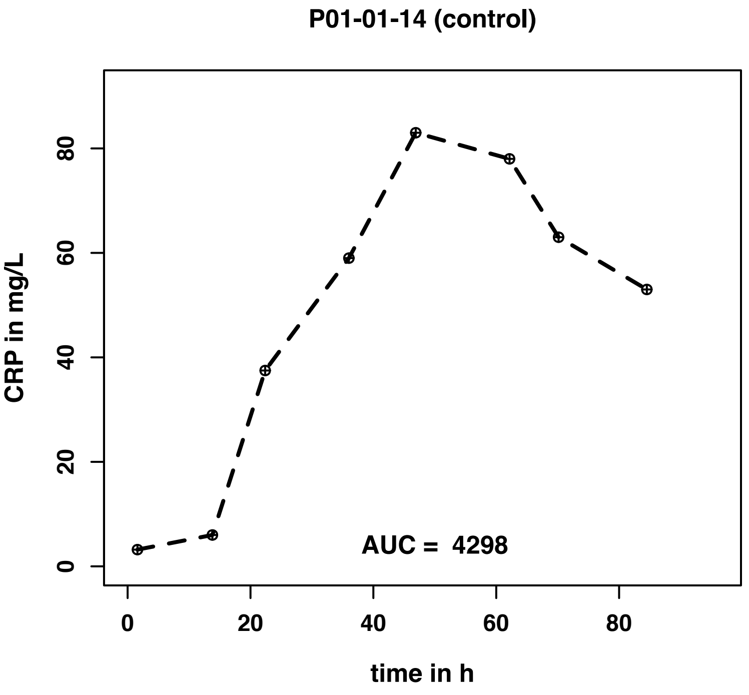

P01-01-14 (control)
CRP in mg/L
time in h
AUC = 4298

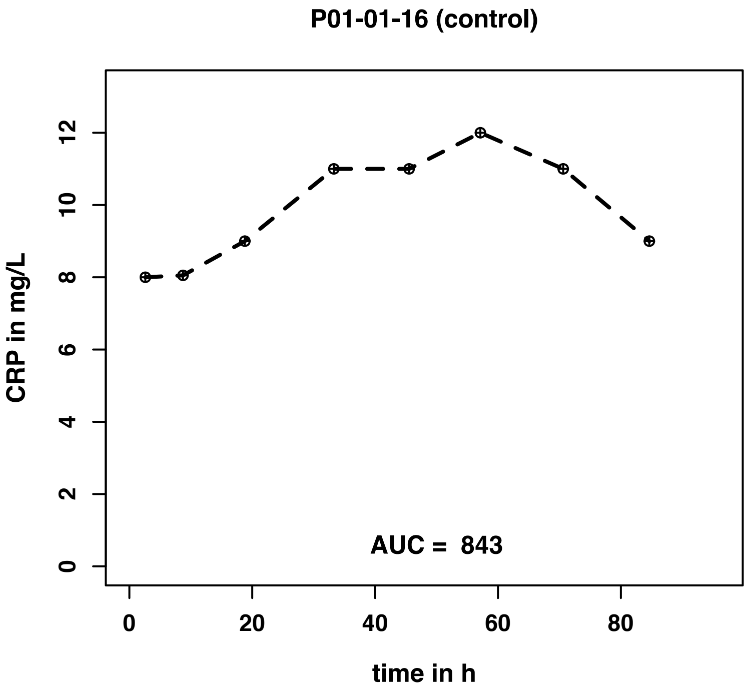

P01-01-16 (control)
CRP in mg/L
time in h
AUC = 843

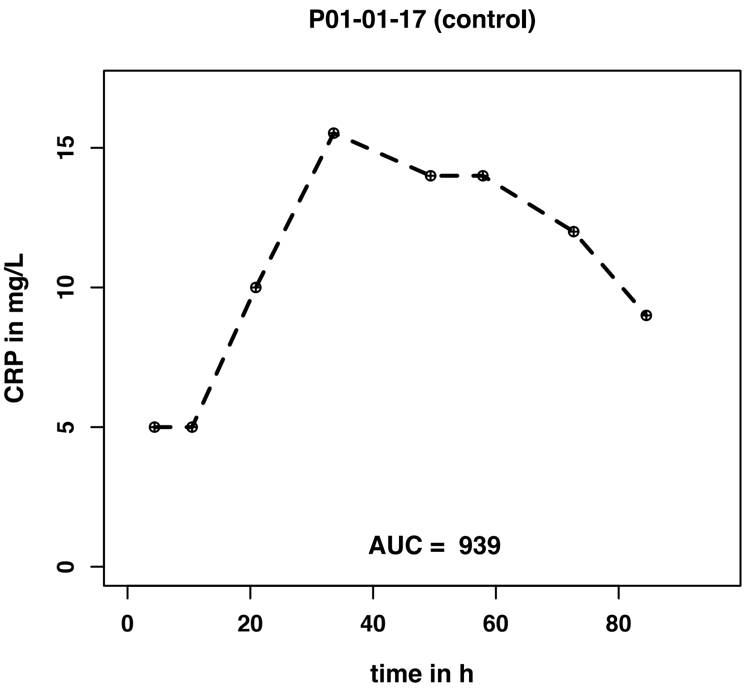
P01-01-17 (control)
CRP in mg/L
0
5
10
15
0
20
40
60
80
time in h
AUC = 939

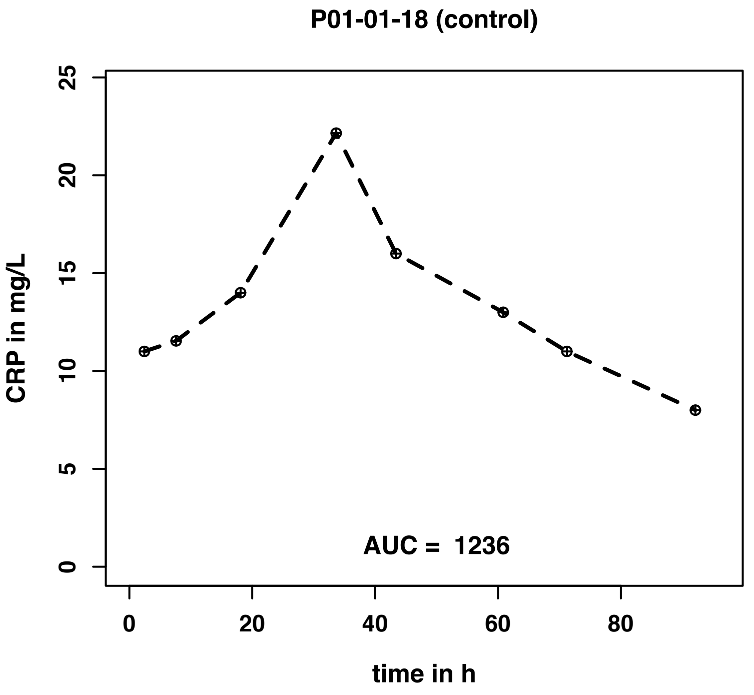
P01-01-18 (control)
CRP in mg/L
0
5
10
15
20
25
0
20
40
60
80
time in h
AUC = 1236

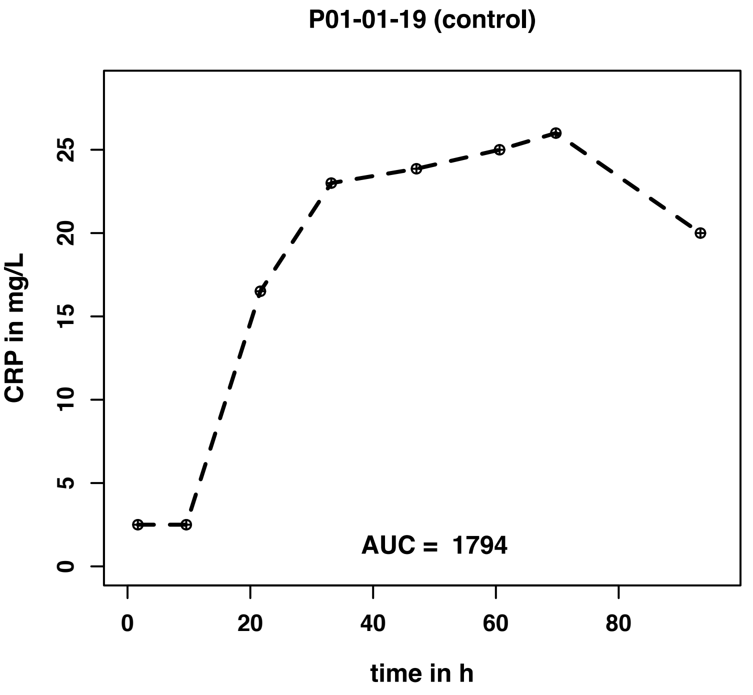
P01-01-19 (control)
CRP in mg/L
0
5
10
15
20
25
0
20
40
60
80
time in h
AUC = 1794

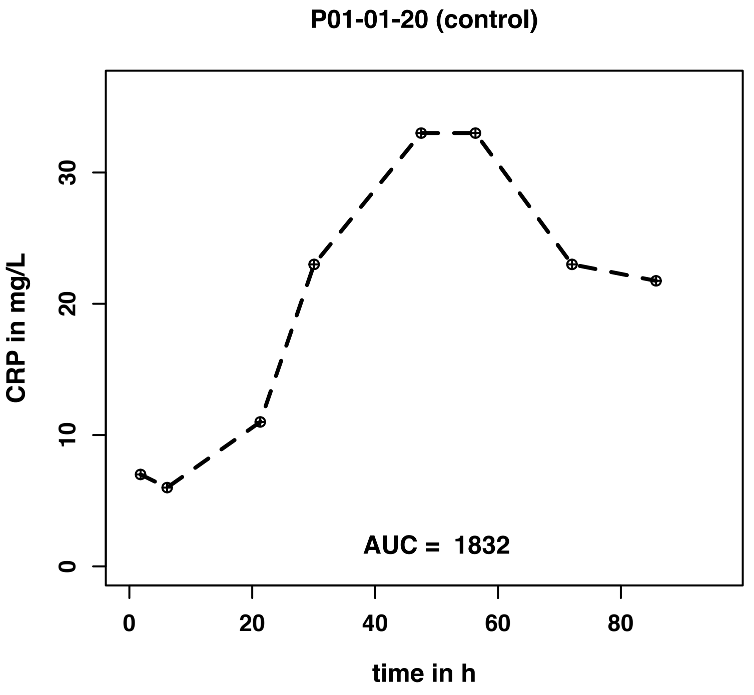
P01-01-20 (control)
CRP in mg/L
0
10
20
30
0
20
40
60
80
time in h
AUC = 1832

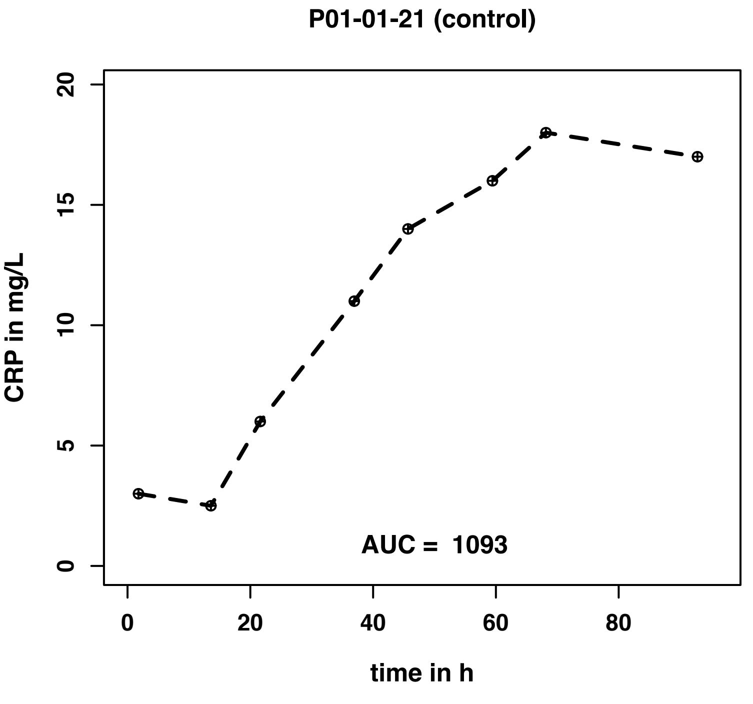
P01-01-21 (control)
CRP in mg/L
0
5
10
15
20
0
20
40
60
80
time in h
AUC = 1093

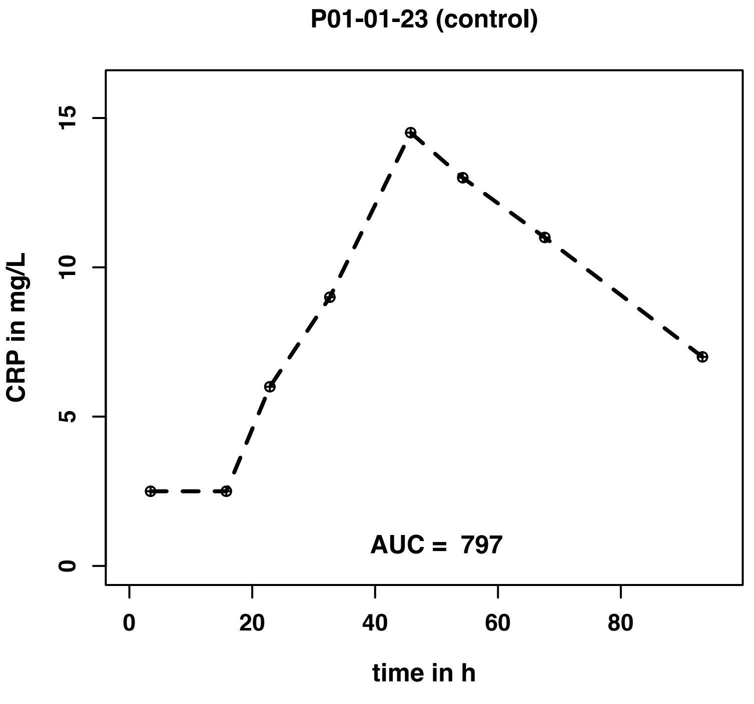
P01-01-23 (control)
CRP in mg/L
0
5
10
15
0
20
40
60
80
time in h
AUC = 797

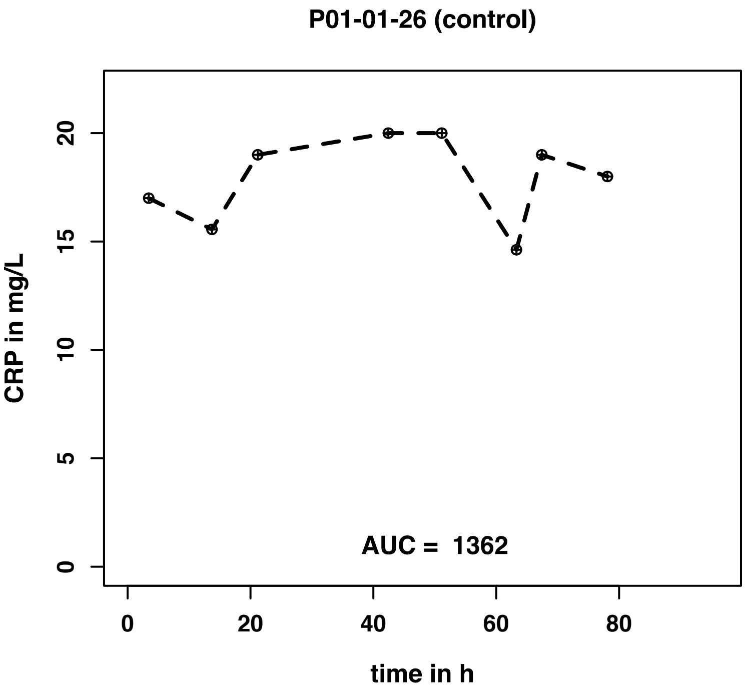
P01-01-26 (control)
CRP in mg/L
0 5 10 15 20
AUC = 1362
0 20 40 60 80
time in h

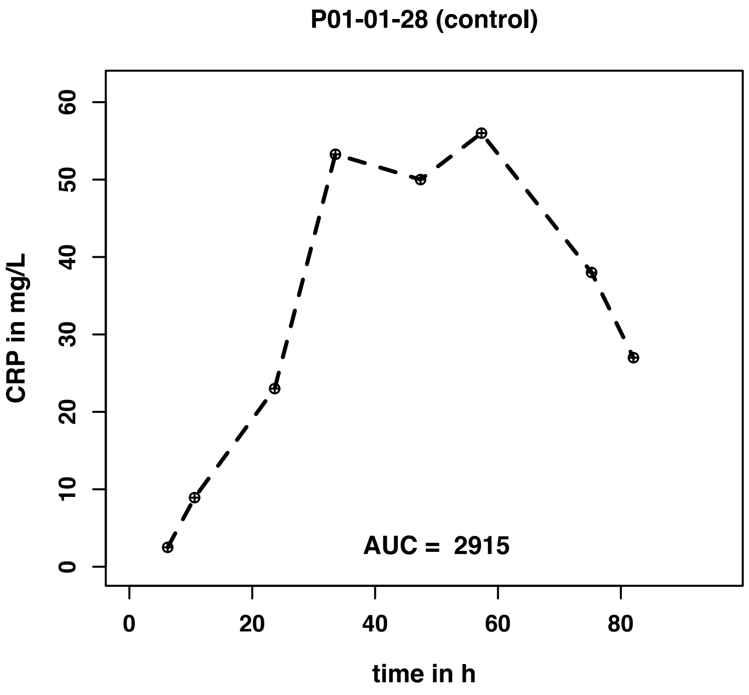
P01-01-28 (control)
CRP in mg/L
0 10 20 30 40 50 60
AUC = 2915
0 20 40 60 80
time in h

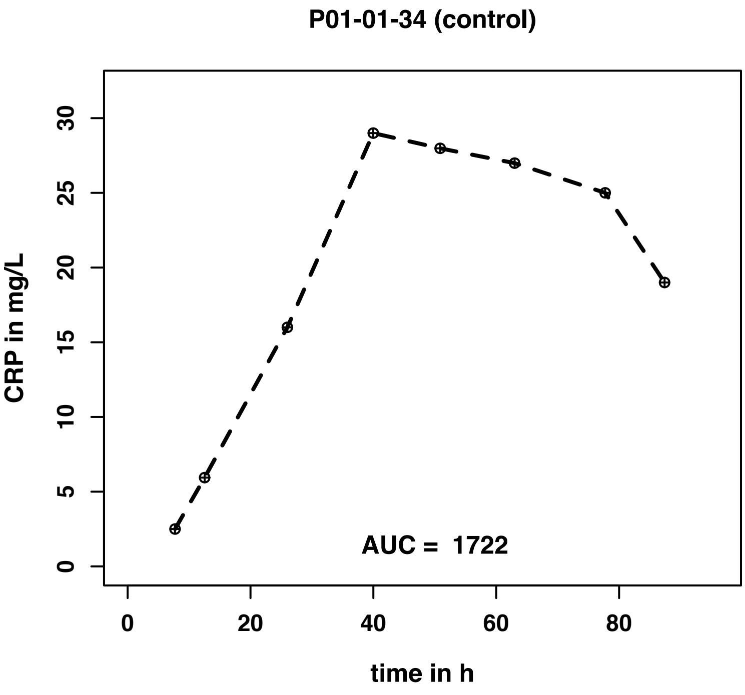
P01-01-34 (control)
CRP in mg/L
0 5 10 15 20 25 30
AUC = 1722
0 20 40 60 80
time in h

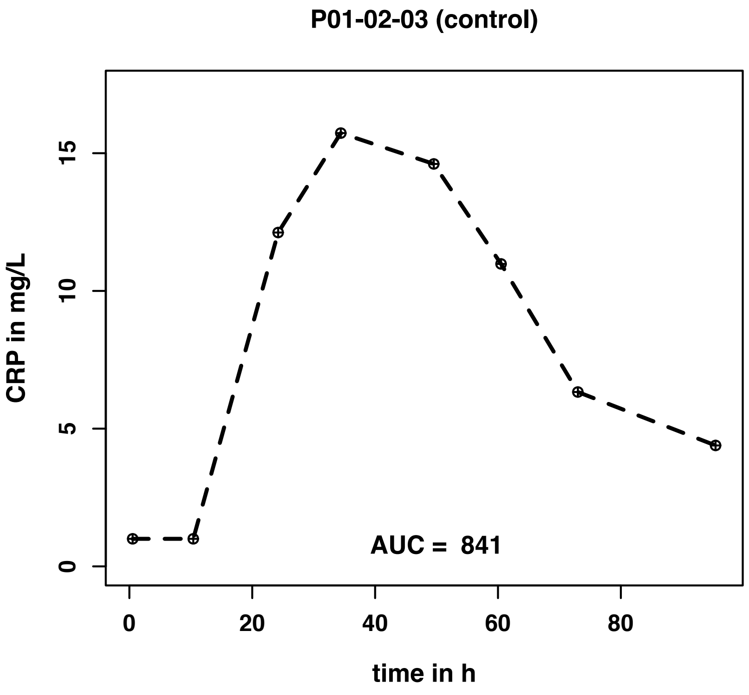
P01-02-03 (control)
CRP in mg/L
0 5 10 15
AUC = 841
0 20 40 60 80
time in h

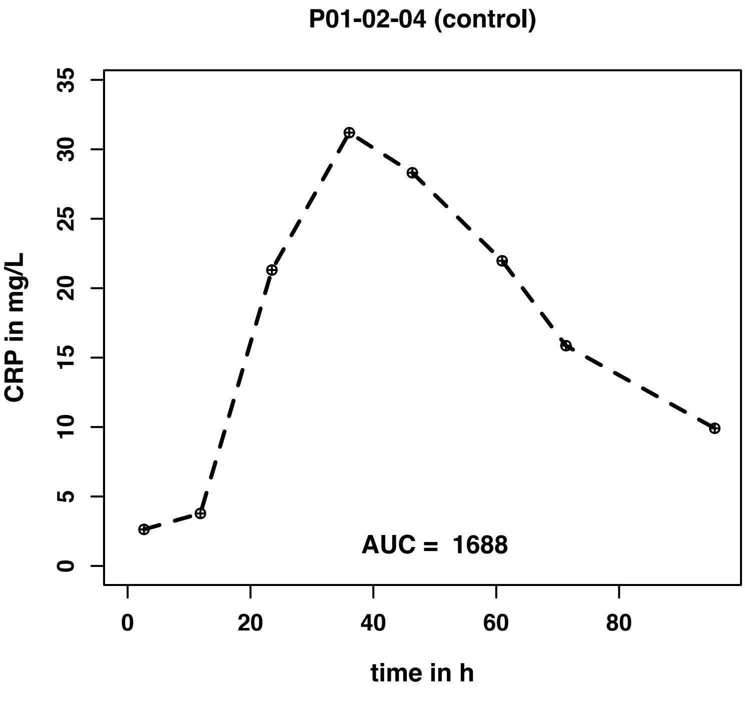
P01-02-04 (control)
CRP in mg/L
0 5 10 15 20 25 30 35
AUC = 1688
0 20 40 60 80
time in h

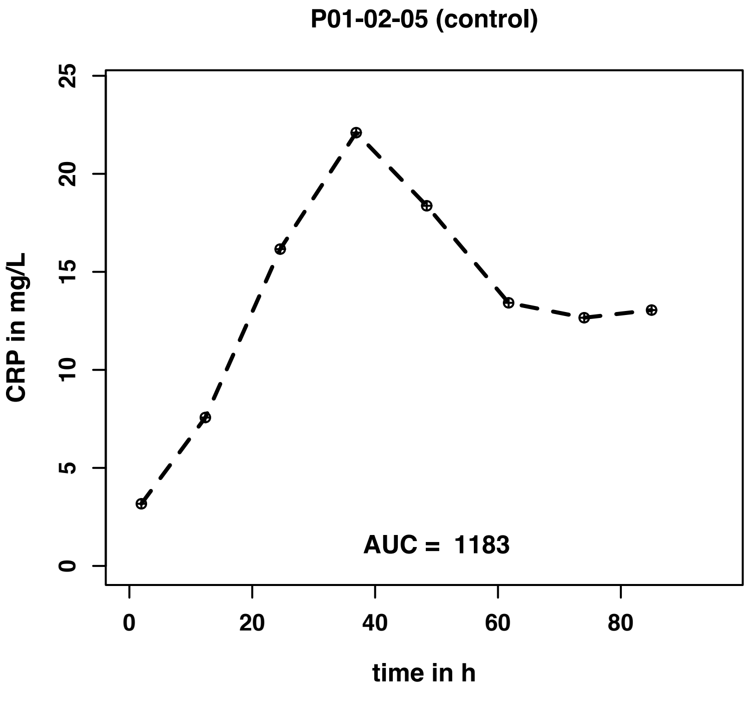
P01-02-05 (control)
CRP in mg/L
0 5 10 15 20 25
AUC = 1183
0 20 40 60 80
time in h

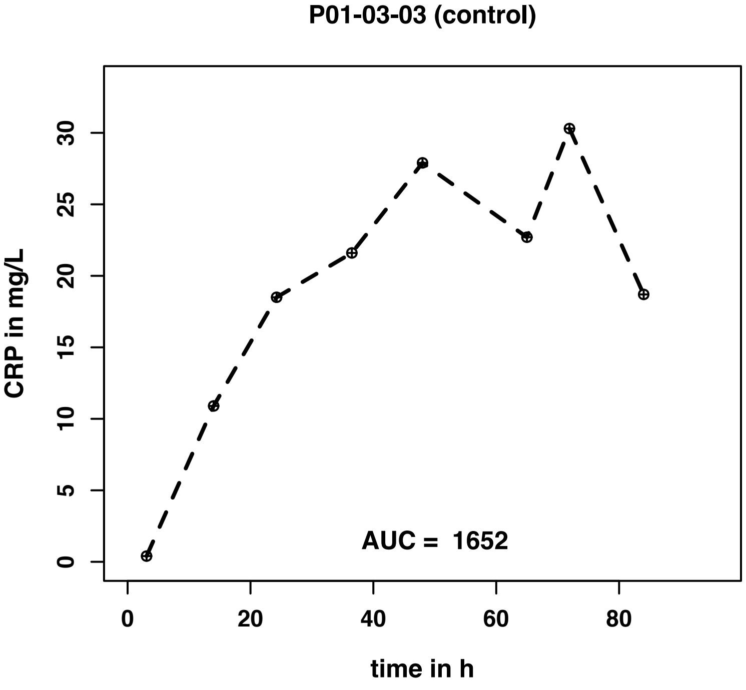
P01-03-03 (control)
CRP in mg/L
0 5 10 15 20 25 30
AUC = 1652
0 20 40 60 80
time in h

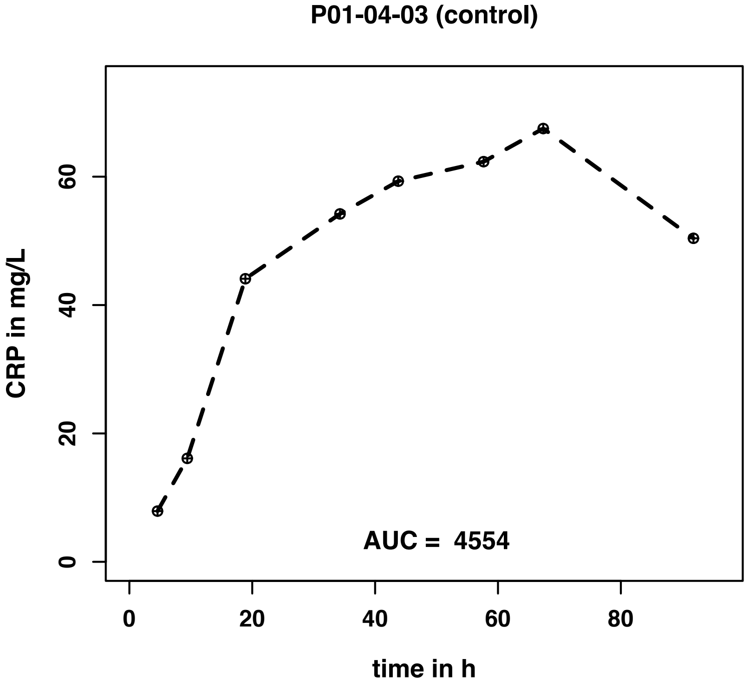
P01-04-03 (control)
CRP in mg/L
0 20 40 60
AUC = 4554
0 20 40 60 80
time in h

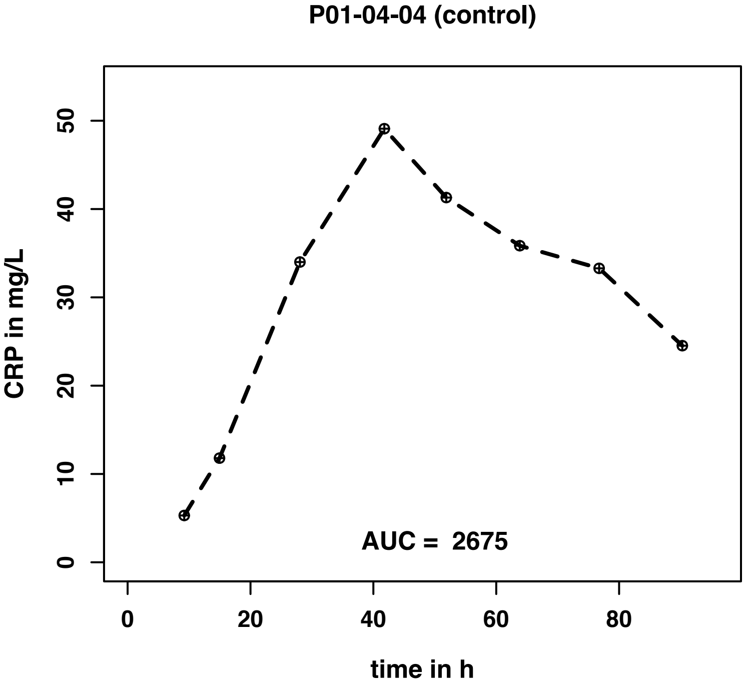
P01-04-04 (control)
CRP in mg/L
0 10 20 30 40 50
AUC = 2675
0 20 40 60 80
time in h

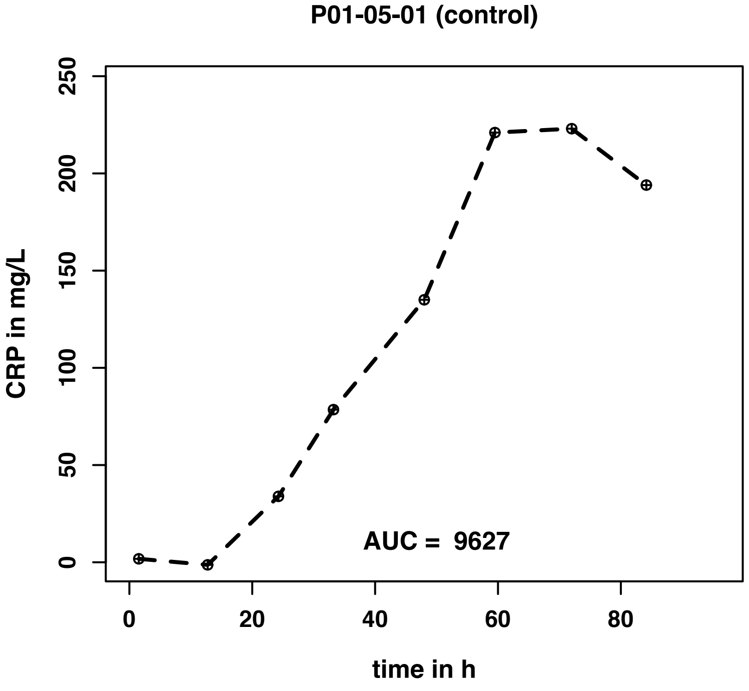
P01-05-01 (control)
CRP in mg/L
0 50 100 150 200 250
AUC = 9627
0 20 40 60 80
time in h

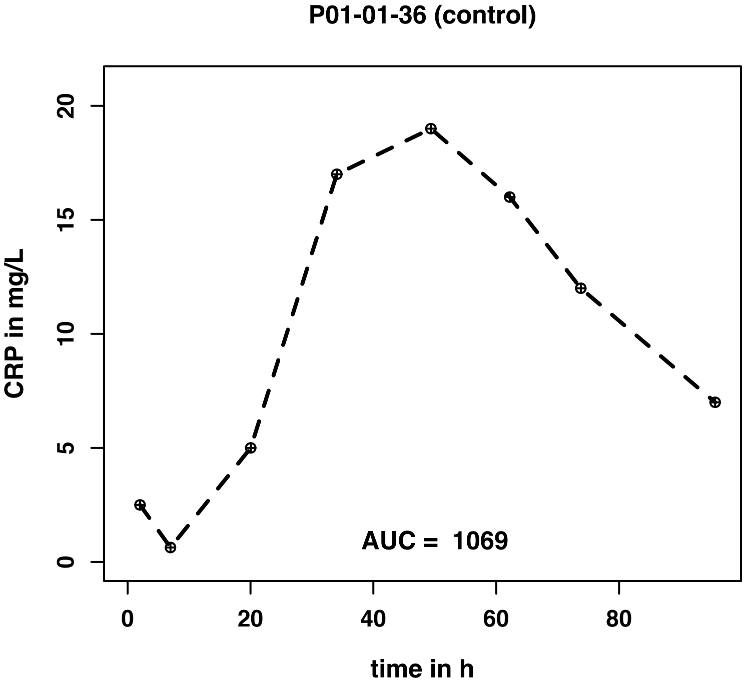
P01-01-36 (control)
CRP in mg/L
0 5 10 15 20
AUC = 1069
0 20 40 60 80
time in h

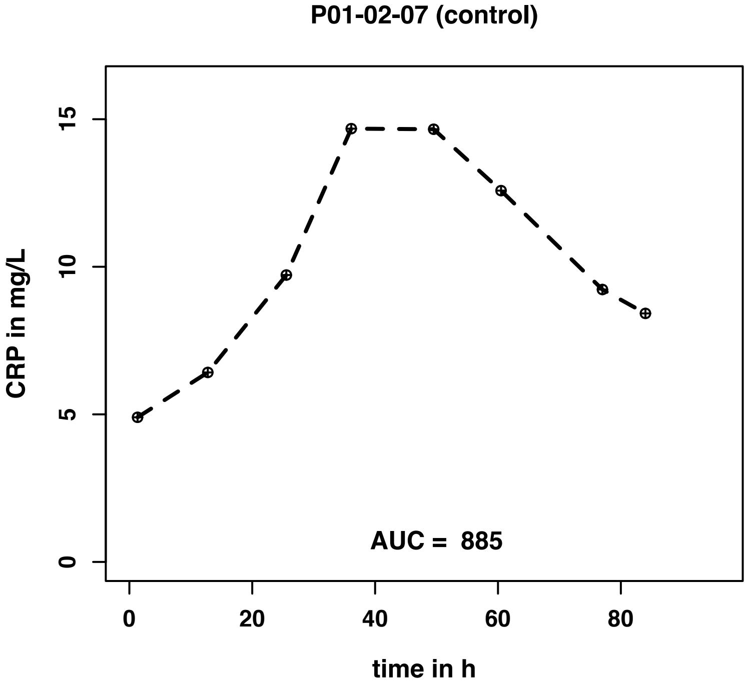
P01-02-07 (control)
CRP in mg/L
0 5 10 15
AUC = 885
0 20 40 60 80
time in h

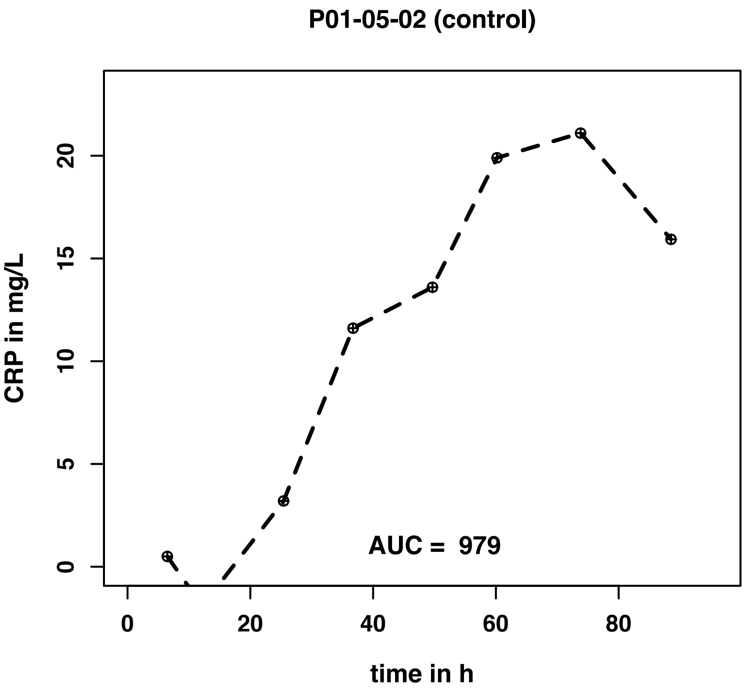
P01-05-02 (control)
CRP in mg/L
0
5
10
15
20
AUC = 979
0
20
40
60
80
time in h

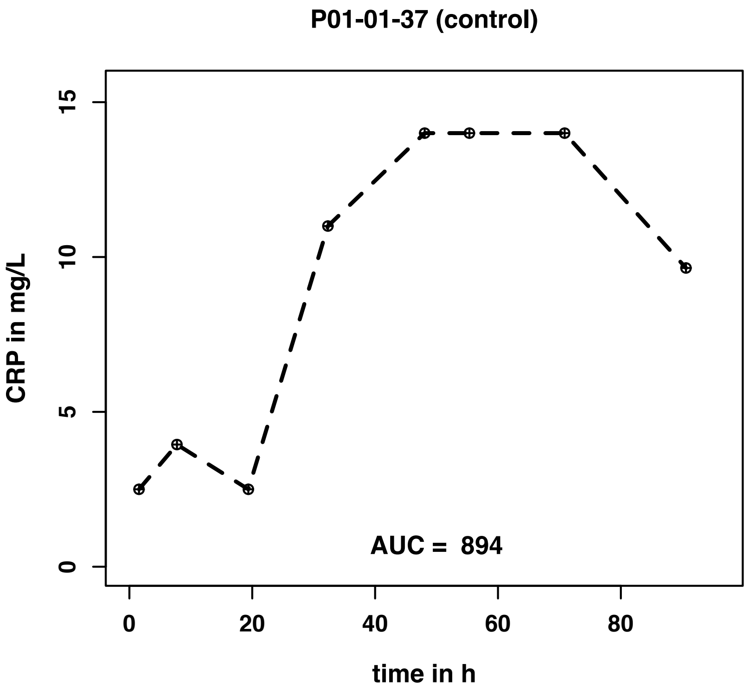
P01-01-37 (control)
CRP in mg/L
0
5
10
15
AUC = 894
0
20
40
60
80
time in h

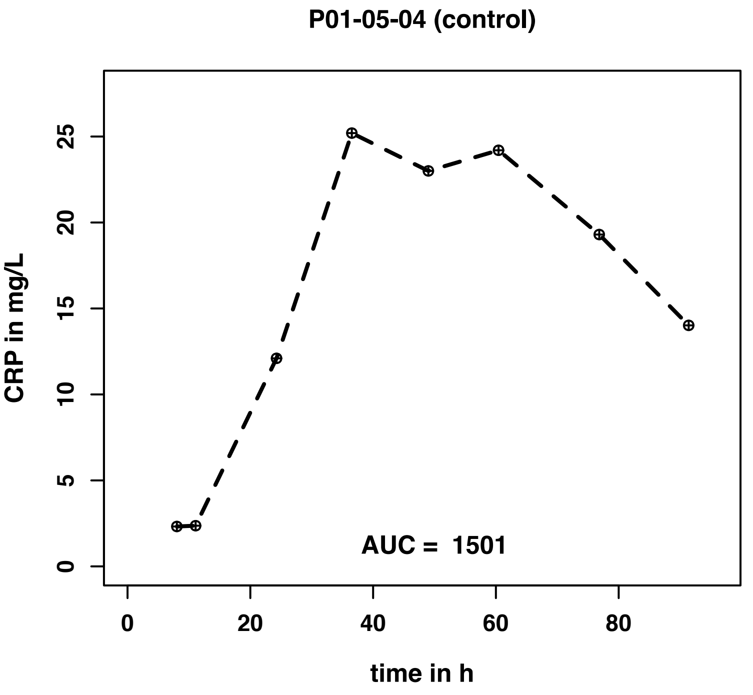
P01-05-04 (control)
CRP in mg/L
0
5
10
15
20
25
AUC = 1501
0
20
40
60
80
time in h

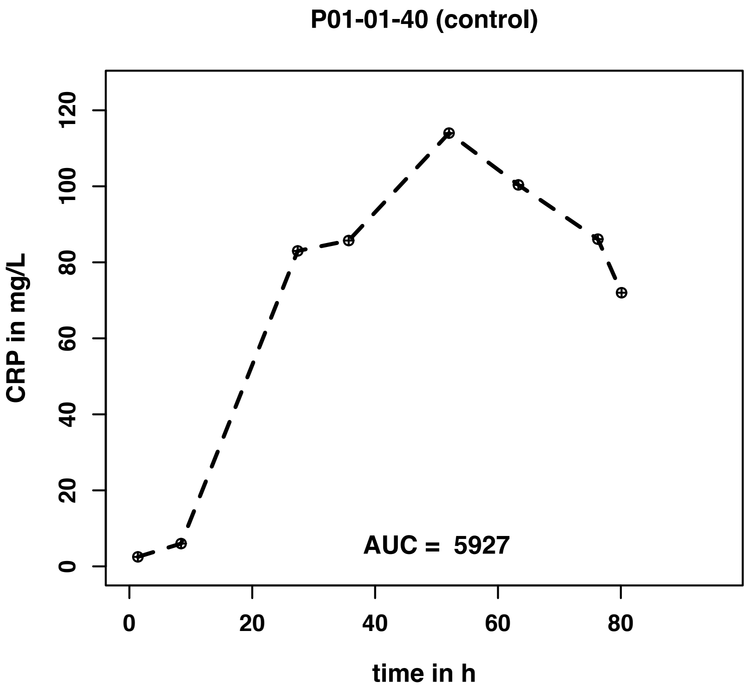
P01-01-40 (control)
CRP in mg/L
0
20
40
60
80
100
120
AUC = 5927
0
20
40
60
80
time in h

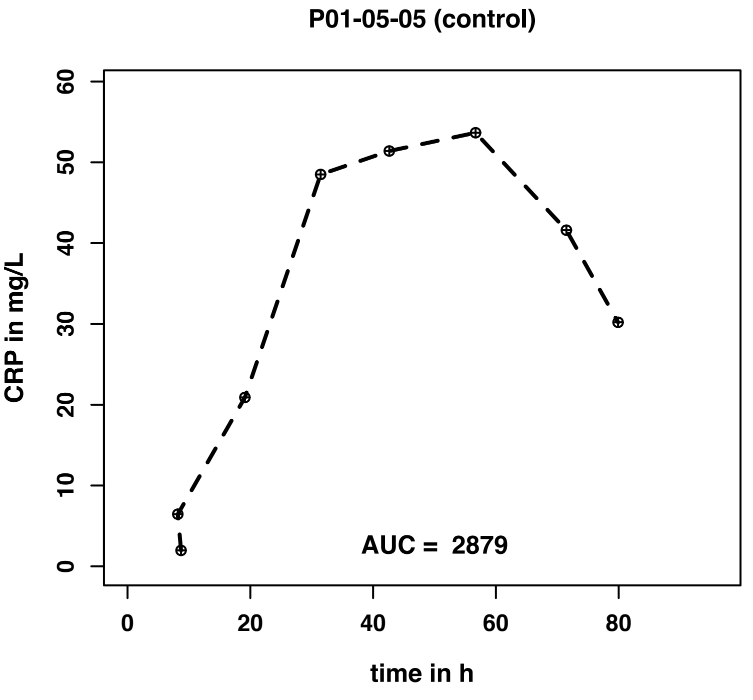
P01-05-05 (control)
CRP in mg/L
0
10
20
30
40
50
60
AUC = 2879
0
20
40
60
80
time in h

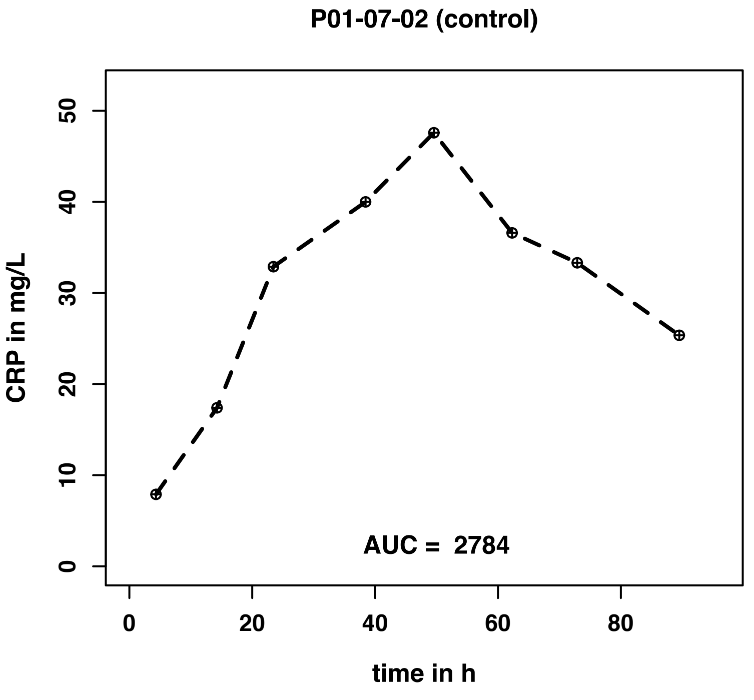
P01-07-02 (control)
CRP in mg/L
0
10
20
30
40
50
AUC = 2784
0
20
40
60
80
time in h

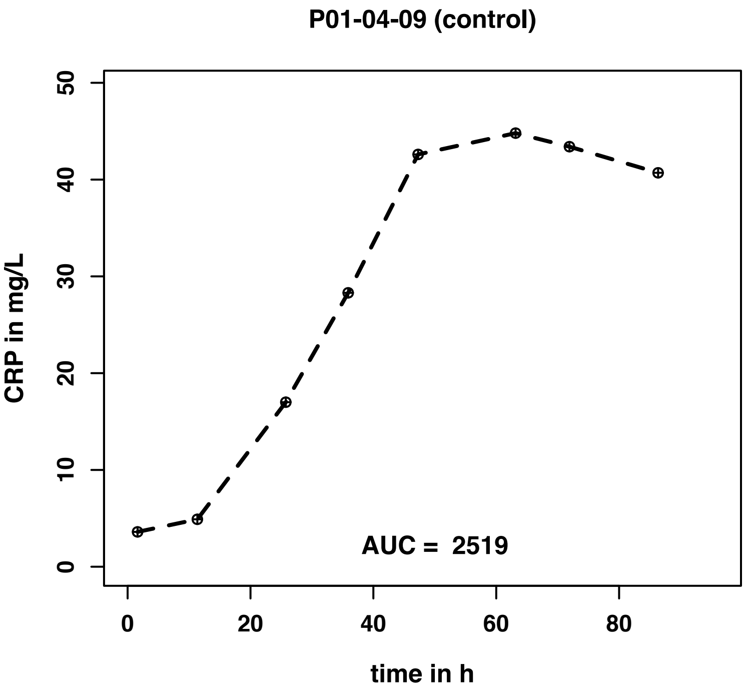

P01-04-09 (control)
CRP in mg/L
0
10
20
30
40
50
AUC = 2519
0
20
40
60
80
time in h

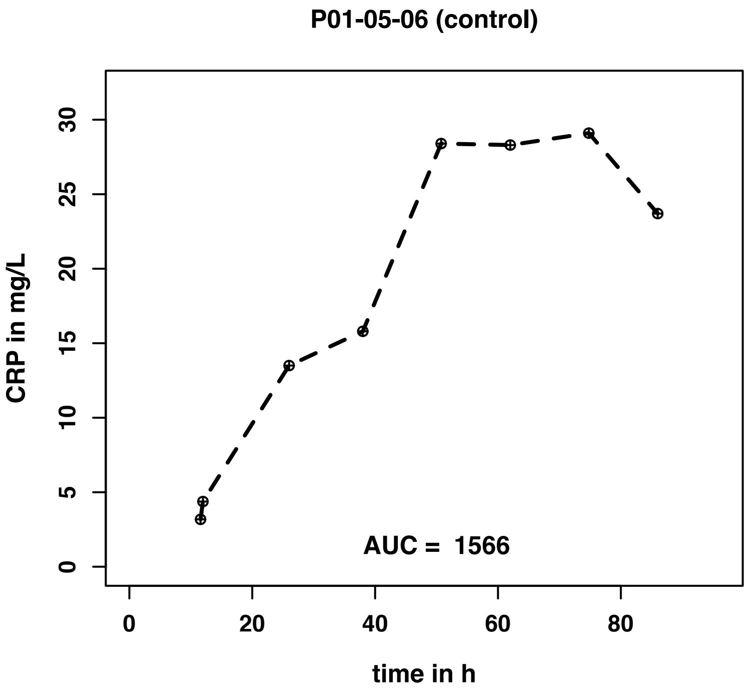

P01-05-06 (control)
CRP in mg/L
0
5
10
15
20
25
30
AUC = 1566
0
20
40
60
80
time in h

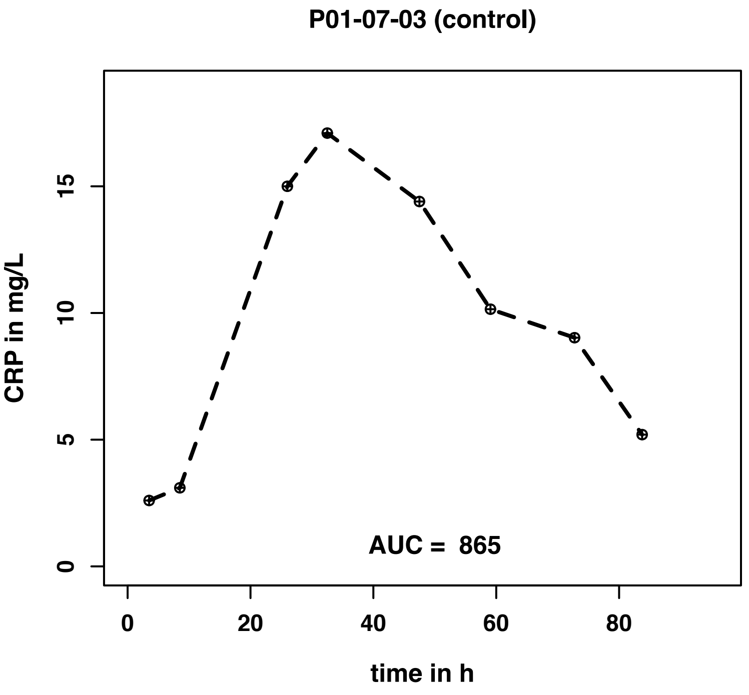

P01-07-03 (control)
CRP in mg/L
0
5
10
15
AUC = 865
0
20
40
60
80
time in h

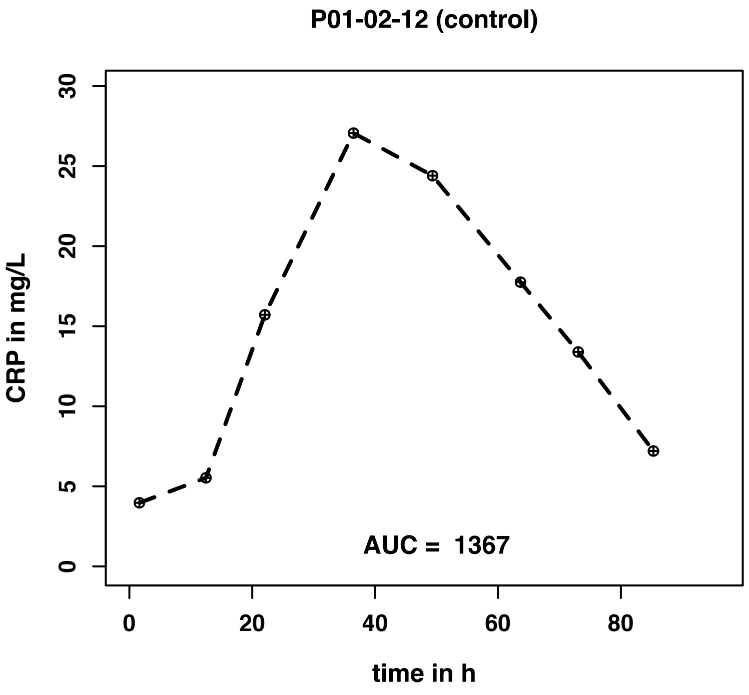

P01-02-12 (control)
CRP in mg/L
0
5
10
15
20
25
30
AUC = 1367
0
20
40
60
80
time in h

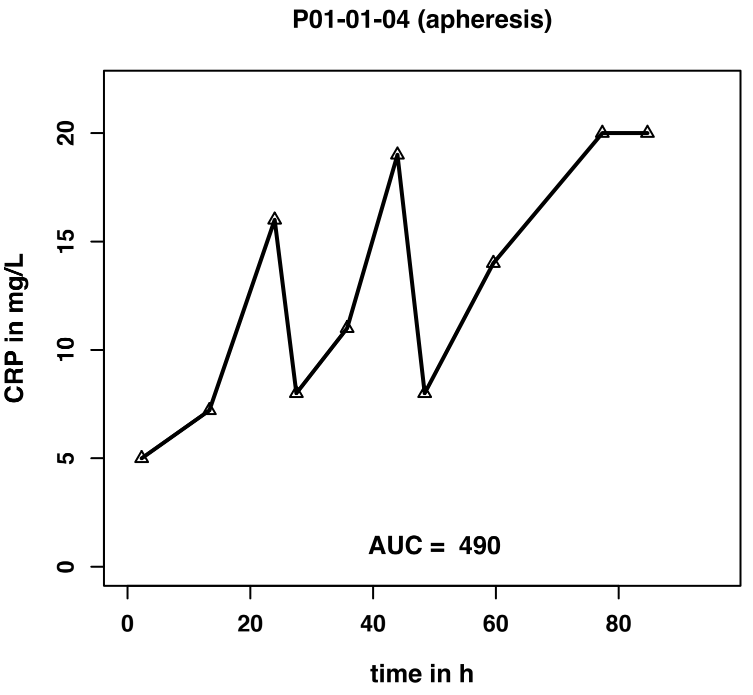
P01-01-04 (apheresis)
CRP in mg/L
time in h
AUC = 490

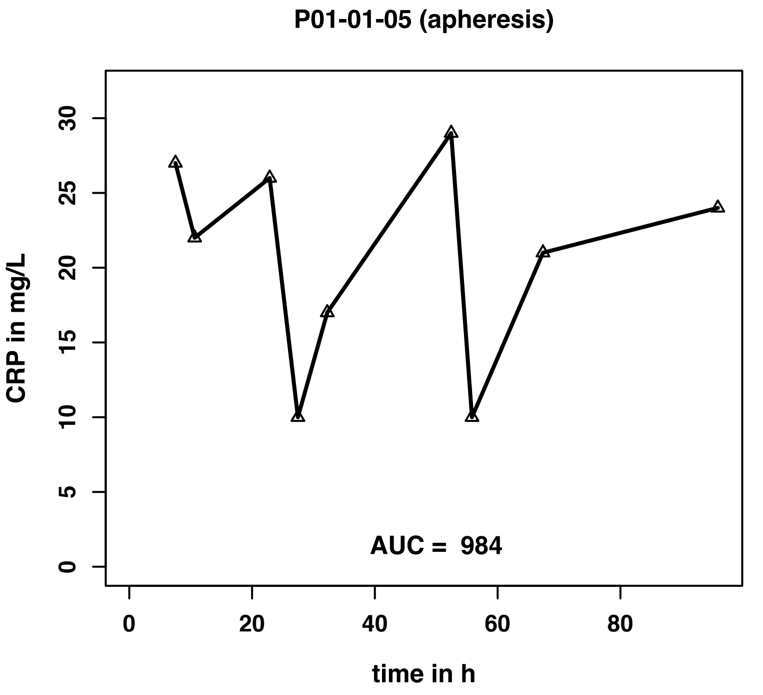
P01-01-05 (apheresis)
CRP in mg/L
time in h
AUC = 984

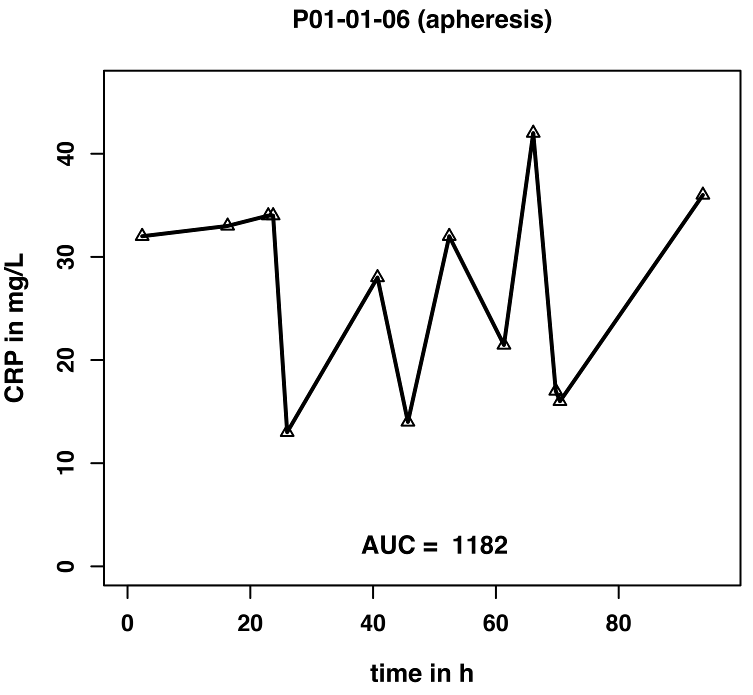
P01-01-06 (apheresis)
CRP in mg/L
time in h
AUC = 1182

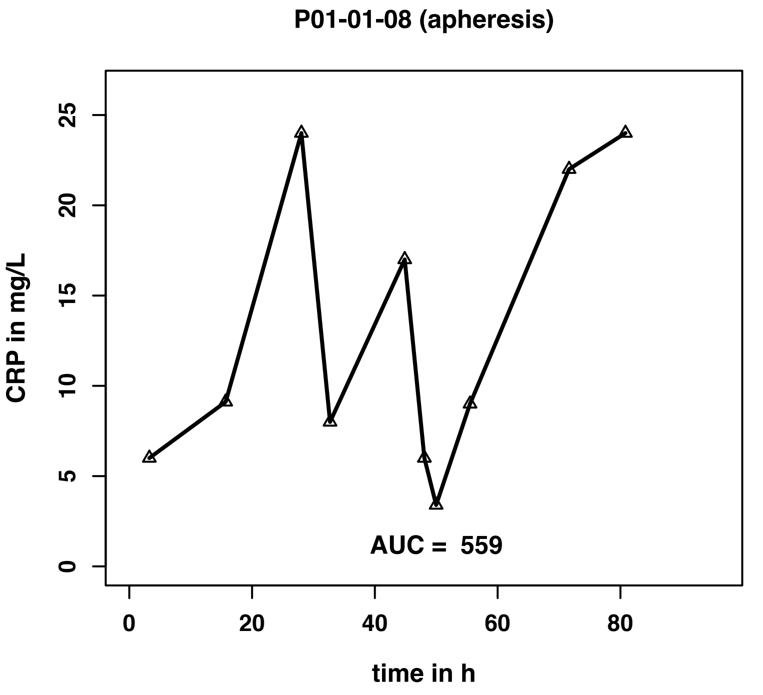
P01-01-08 (apheresis)
CRP in mg/L
time in h
AUC = 559

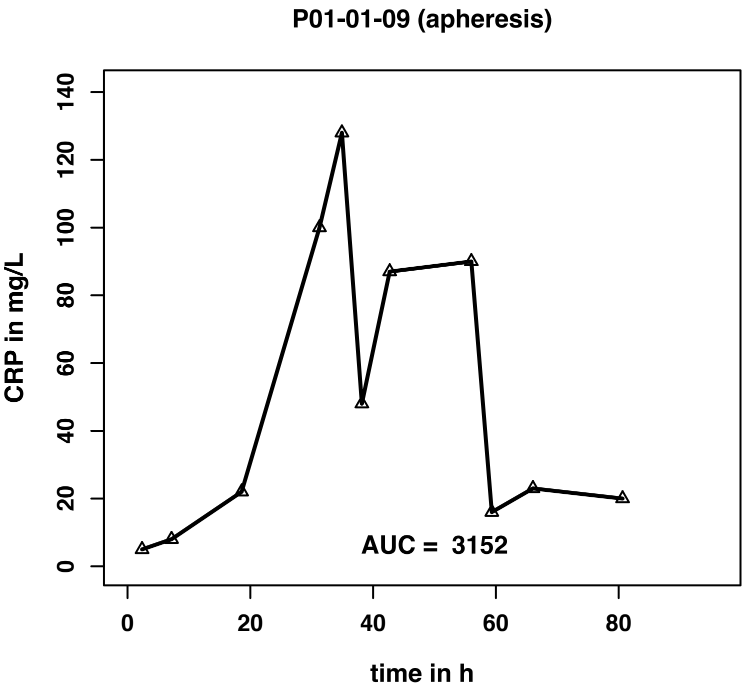
P01-01-09 (apheresis)
CRP in mg/L
time in h
AUC = 3152

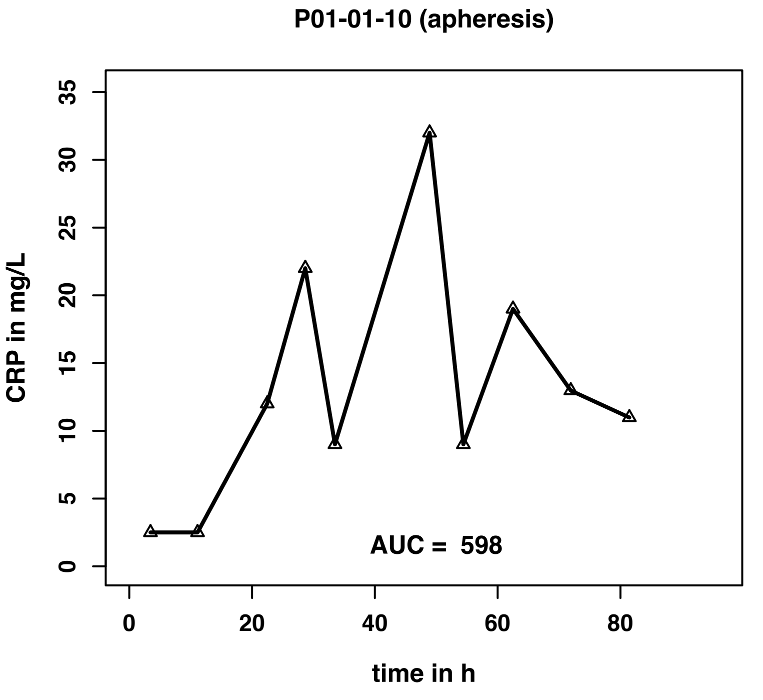
P01-01-10 (apheresis)
CRP in mg/L
time in h
AUC = 598

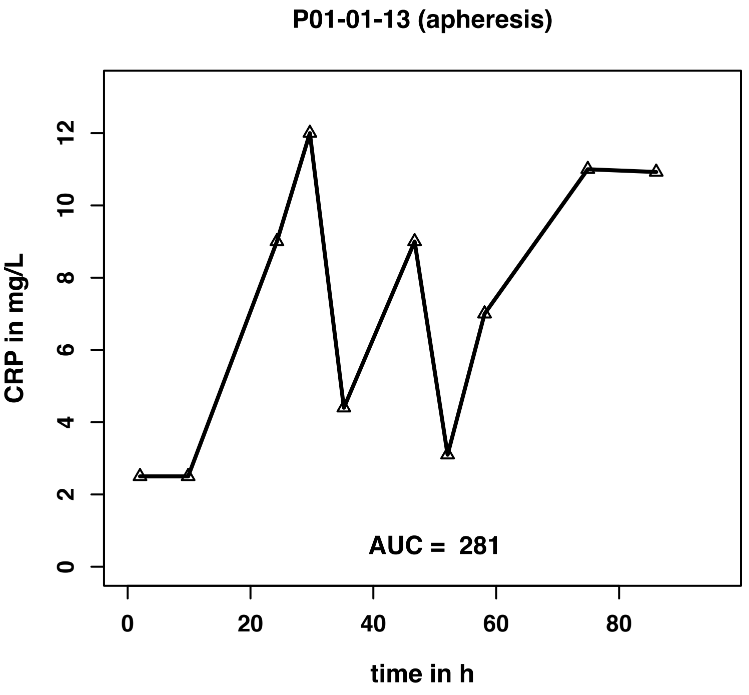
P01-01-13 (apheresis)
CRP in mg/L
time in h
AUC = 281
0
2
4
6
8
10
12
0
20
40
60
80

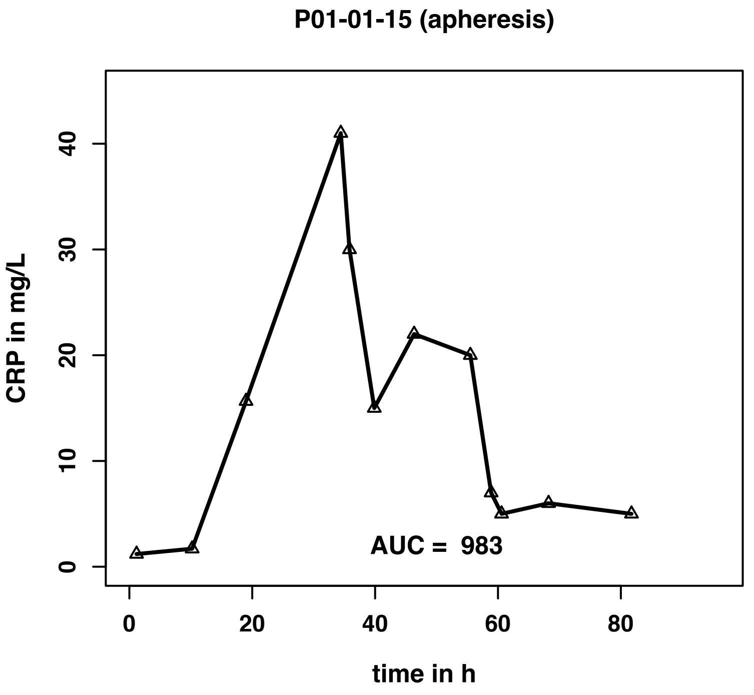
P01-01-15 (apheresis)
CRP in mg/L
time in h
AUC = 983
0
10
20
30
40
0
20
40
60
80

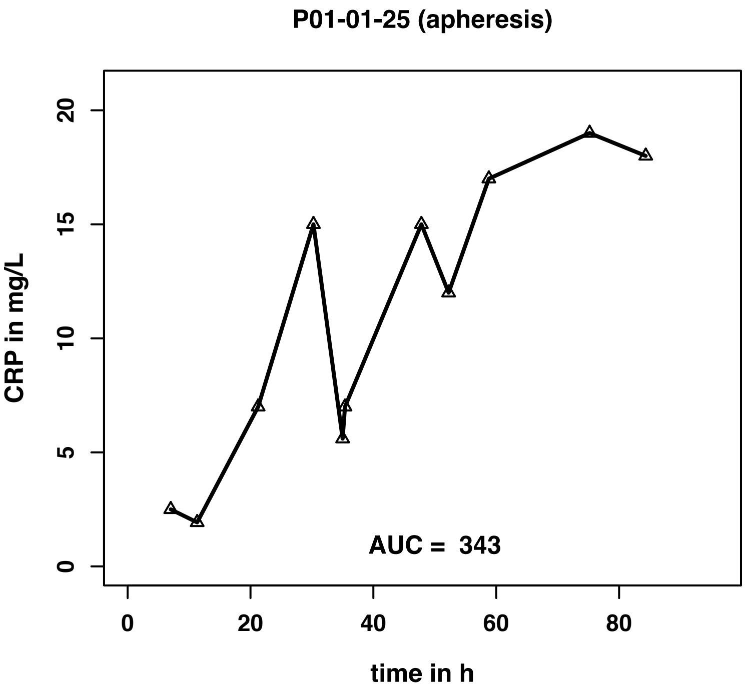
P01-01-25 (apheresis)
CRP in mg/L
time in h
AUC = 343
0
5
10
15
20
0
20
40
60
80

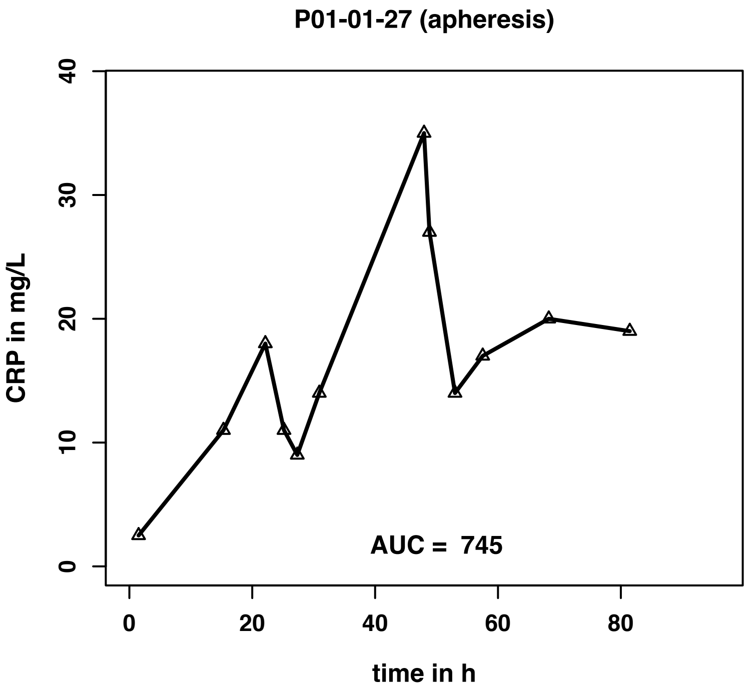
P01-01-27 (apheresis)
CRP in mg/L
time in h
AUC = 745
0
10
20
30
40
0
20
40
60
80

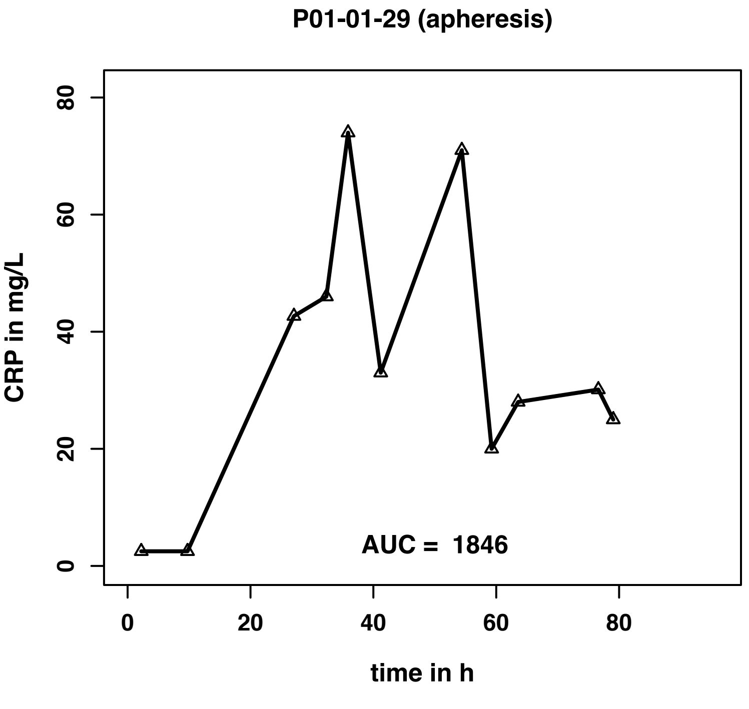
P01-01-29 (apheresis)
CRP in mg/L
time in h
AUC = 1846
0
20
40
60
80
0
20
40
60
80

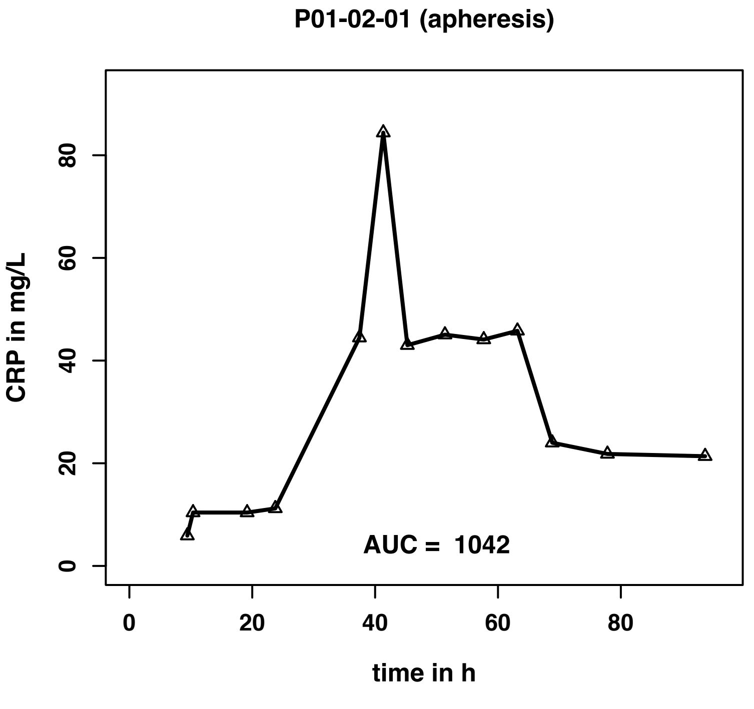
P01-02-01 (apheresis)
CRP in mg/L
time in h
AUC = 1042
0
20
40
60
80
0
20
40
60
80

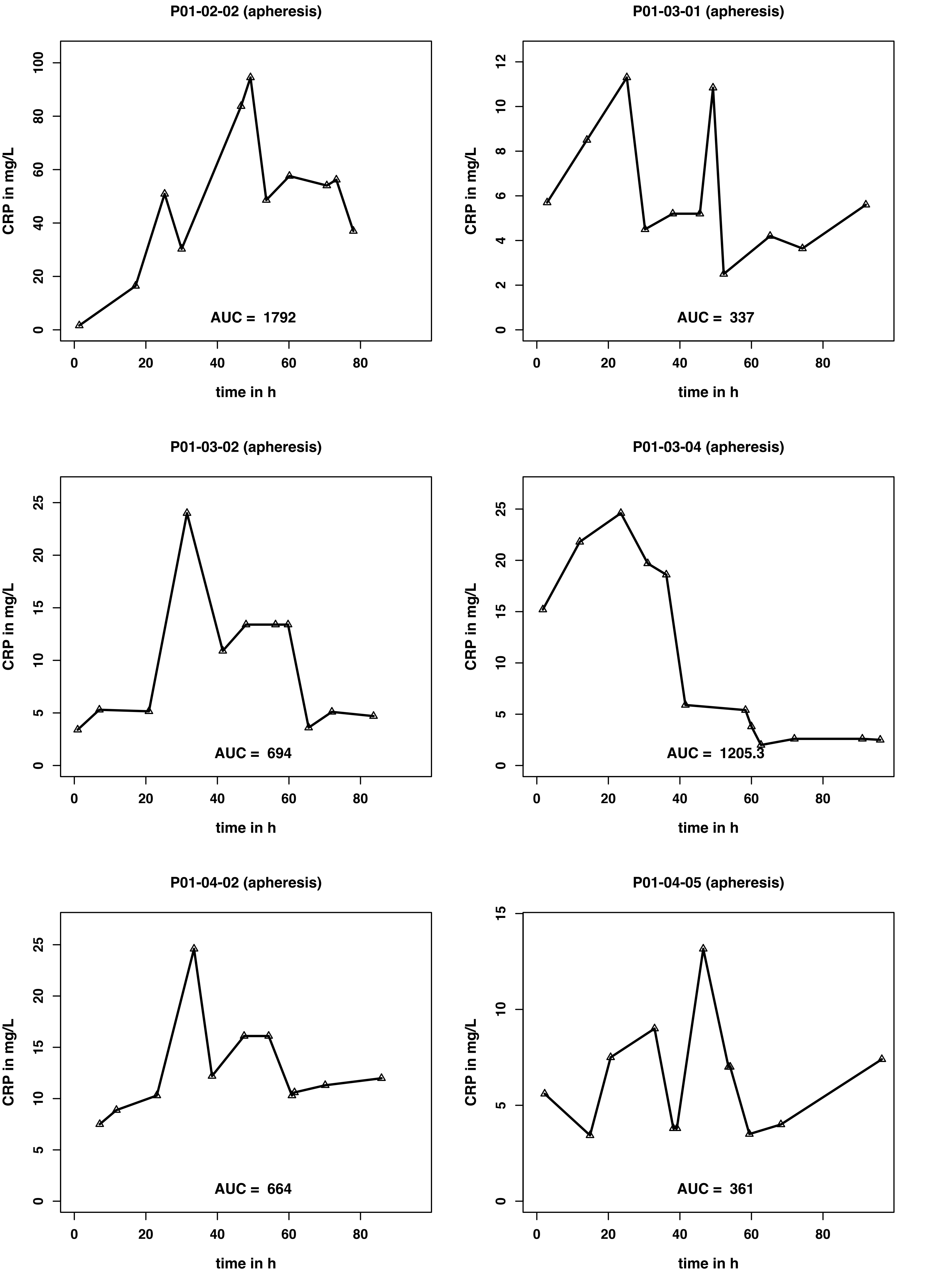

P01-02-02 (apheresis)
CRP in mg/L
time in h
AUC = 1792
P01-03-01 (apheresis)
CRP in mg/L
time in h
AUC = 337
P01-03-02 (apheresis)
CRP in mg/L
time in h
AUC = 694
P01-03-04 (apheresis)
CRP in mg/L
time in h
AUC = 1205.3
P01-04-02 (apheresis)
CRP in mg/L
time in h
AUC = 664
P01-04-05 (apheresis)
CRP in mg/L
time in h
AUC = 361

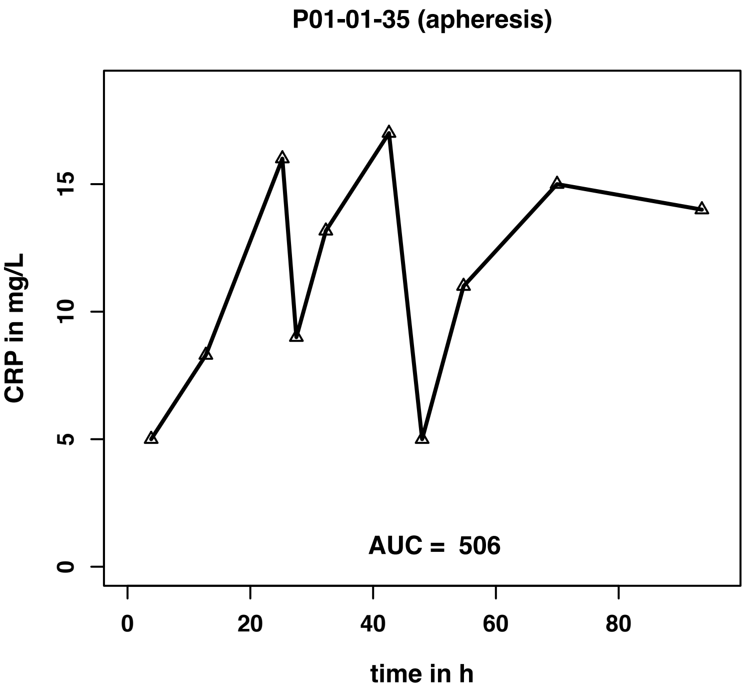
P01-01-35 (apheresis)
CRP in mg/L
time in h
AUC = 506

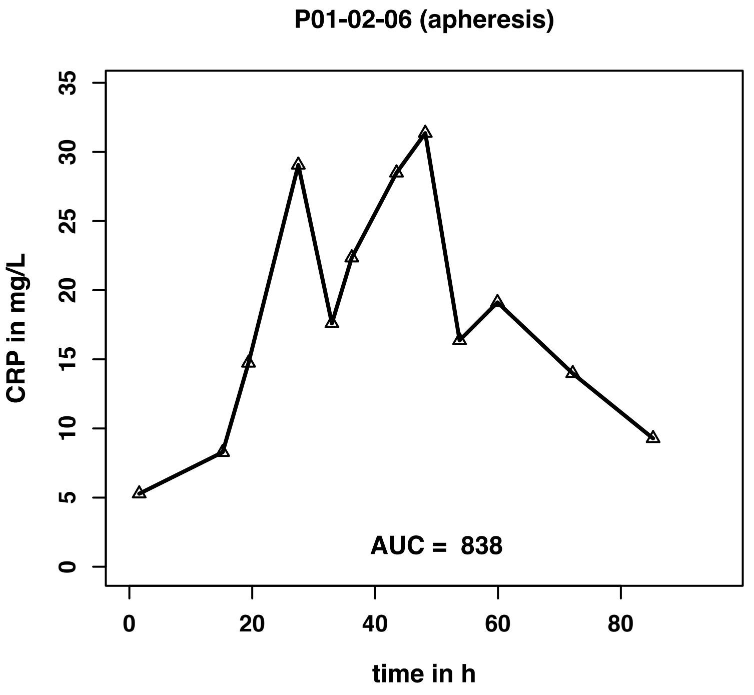
P01-02-06 (apheresis)
CRP in mg/L
time in h
AUC = 838

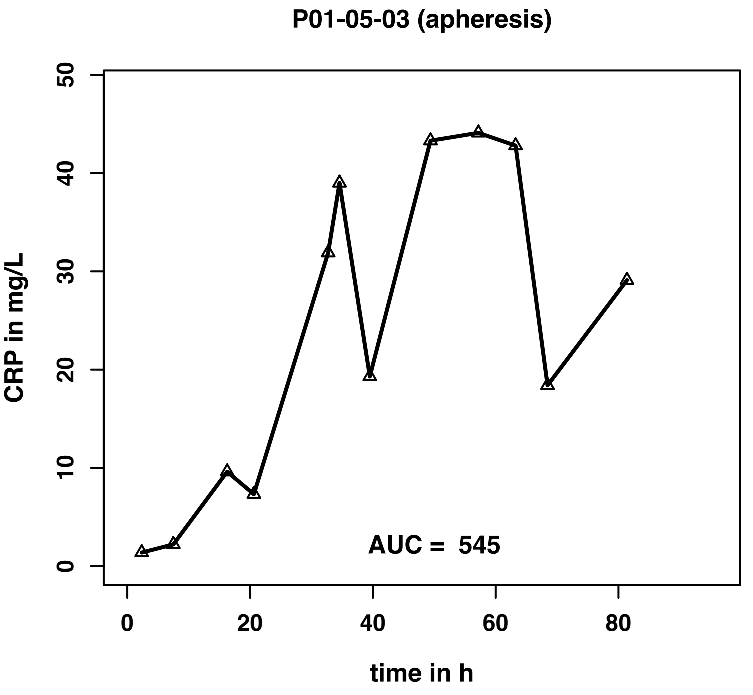
P01-05-03 (apheresis)
CRP in mg/L
time in h
AUC = 545

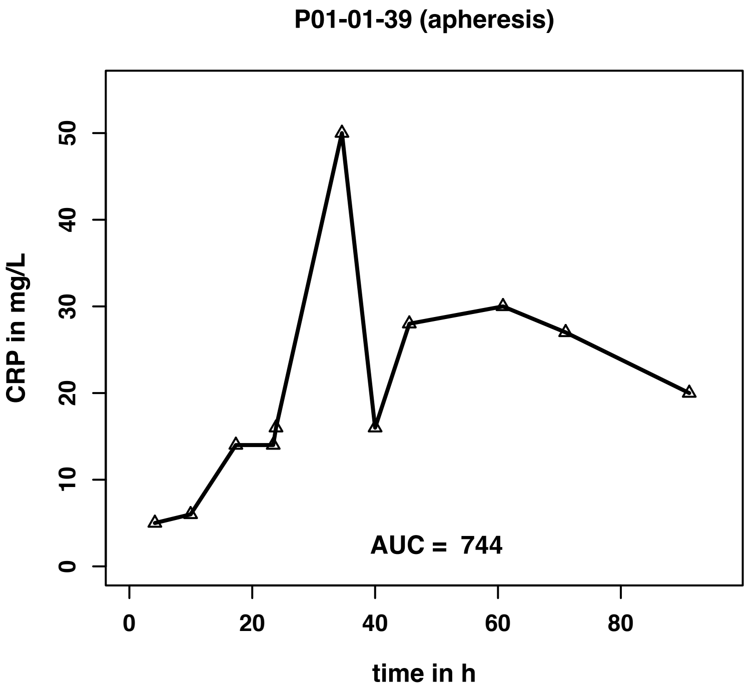
P01-01-39 (apheresis)
CRP in mg/L
time in h
AUC = 744

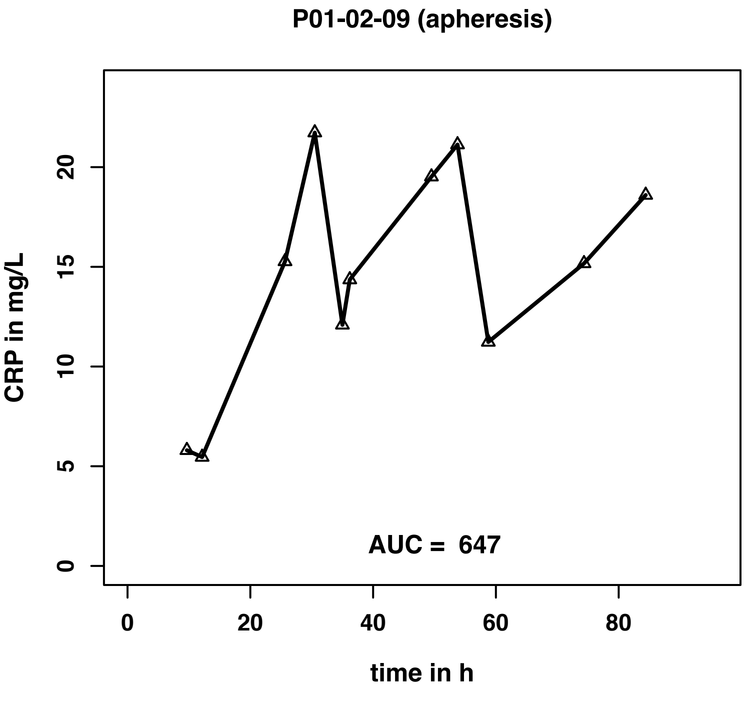
P01-02-09 (apheresis)
CRP in mg/L
time in h
AUC = 647

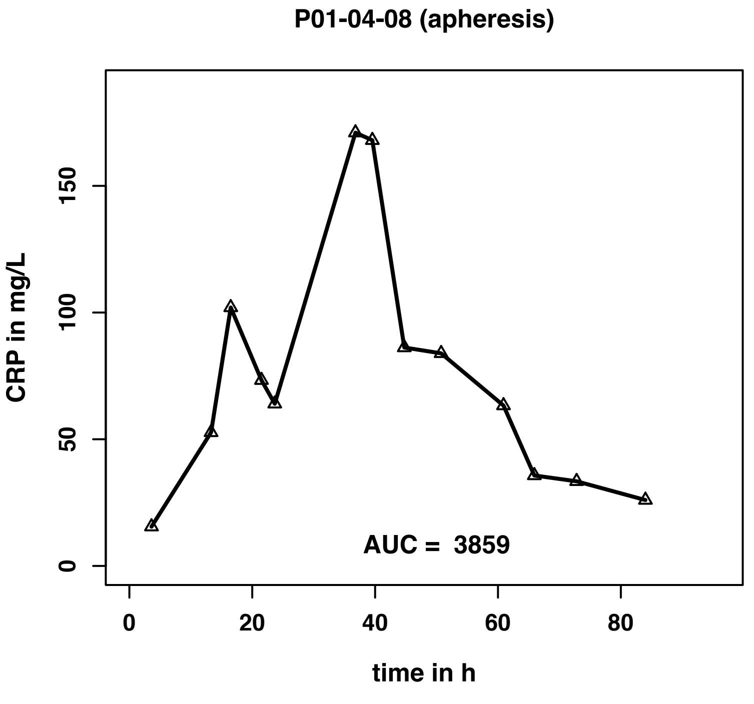
P01-04-08 (apheresis)
CRP in mg/L
time in h
AUC = 3859

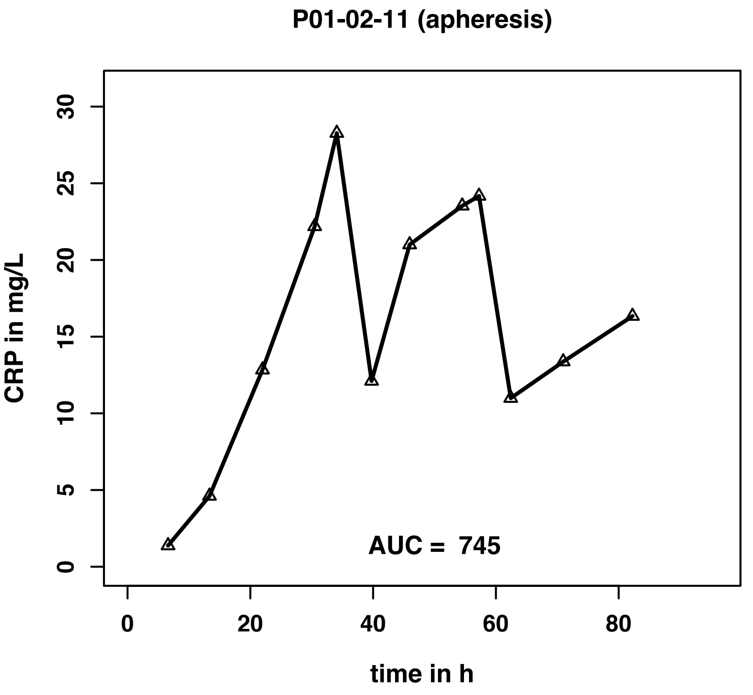
P01-02-11 (apheresis)
CRP in mg/L
time in h
AUC = 745

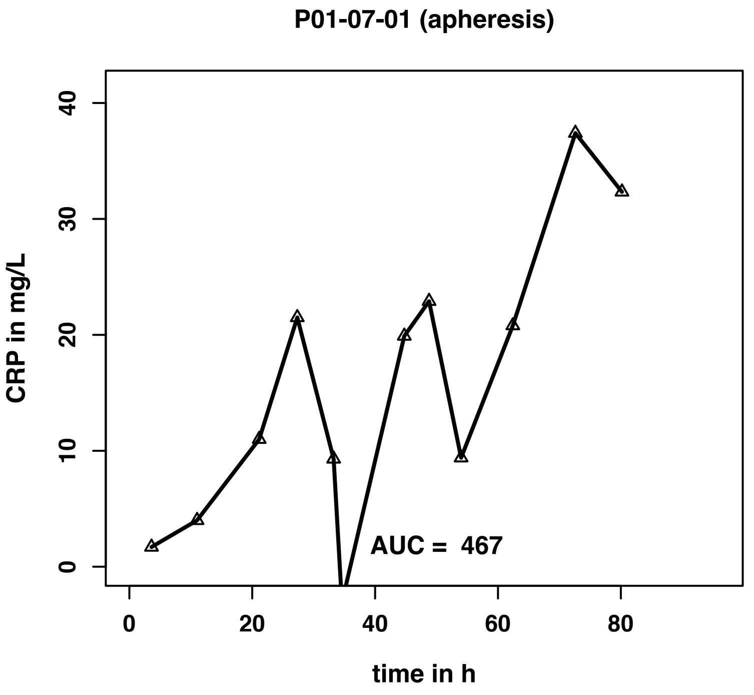
P01-07-01 (apheresis)
CRP in mg/L
time in h
AUC = 467

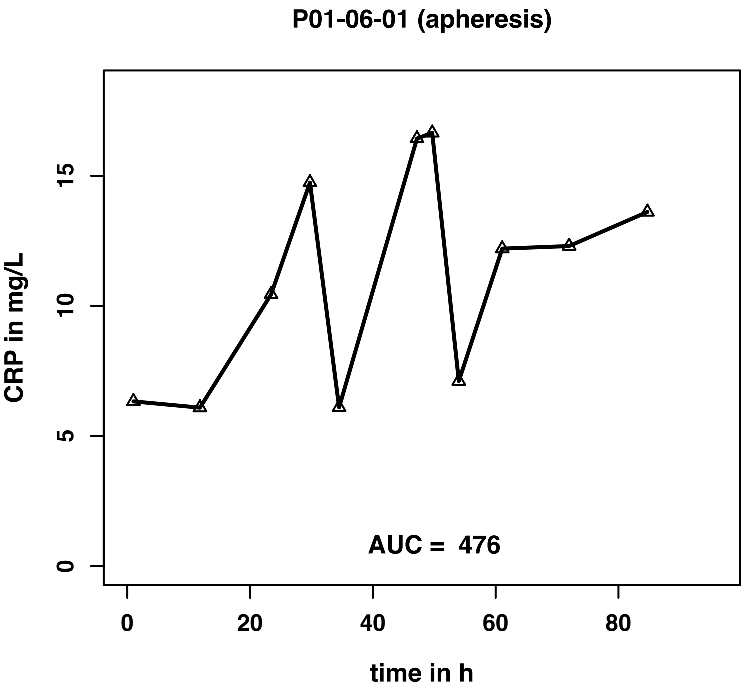
P01-06-01 (apheresis)
CRP in mg/L
time in h
AUC = 476

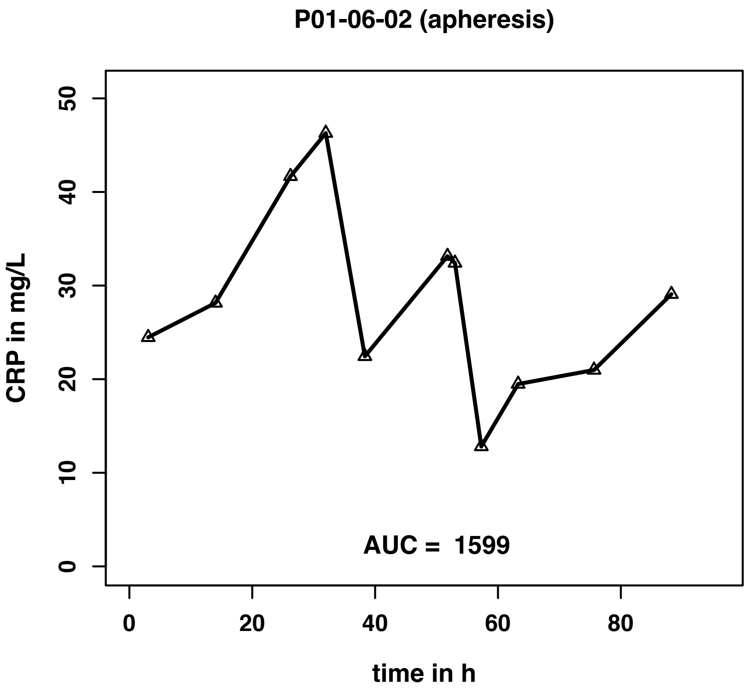
P01-06-02 (apheresis)
CRP in mg/L
time in h
AUC = 1599

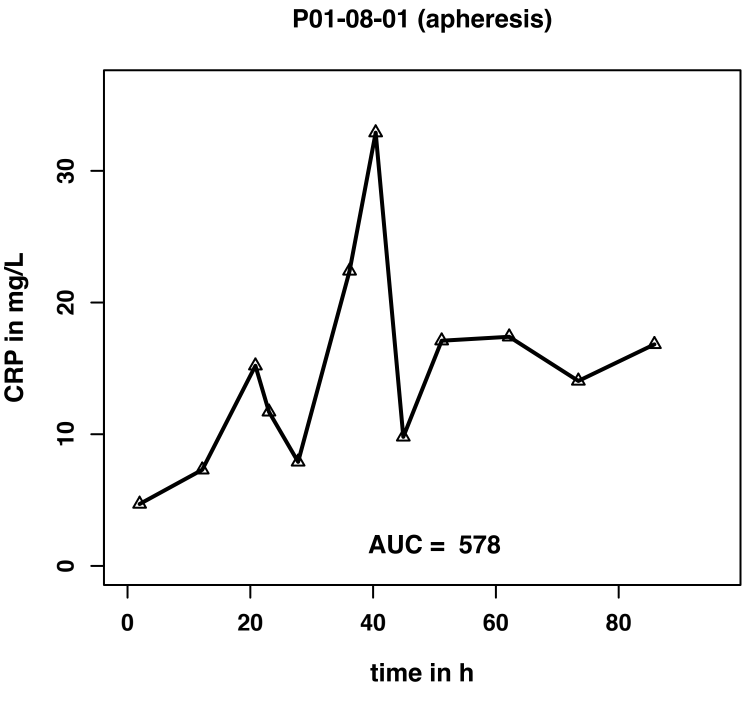
P01-08-01 (apheresis)
CRP in mg/L
time in h
AUC = 578

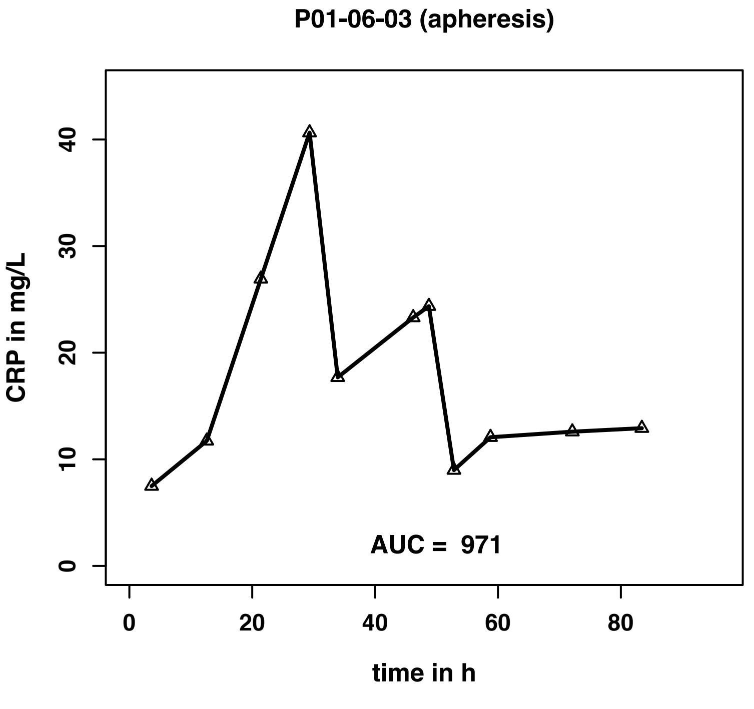
P01-06-03 (apheresis)
CRP in mg/L
time in h
AUC = 971

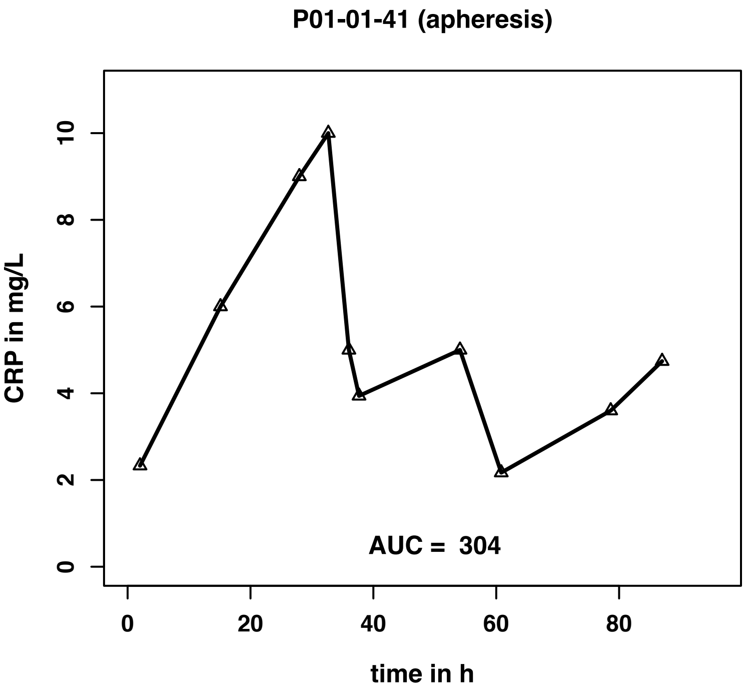

P01-01-41 (apheresis)
CRP in mg/L
time in h
AUC = 304
0
2
4
6
8
10
0
20
40
60
80

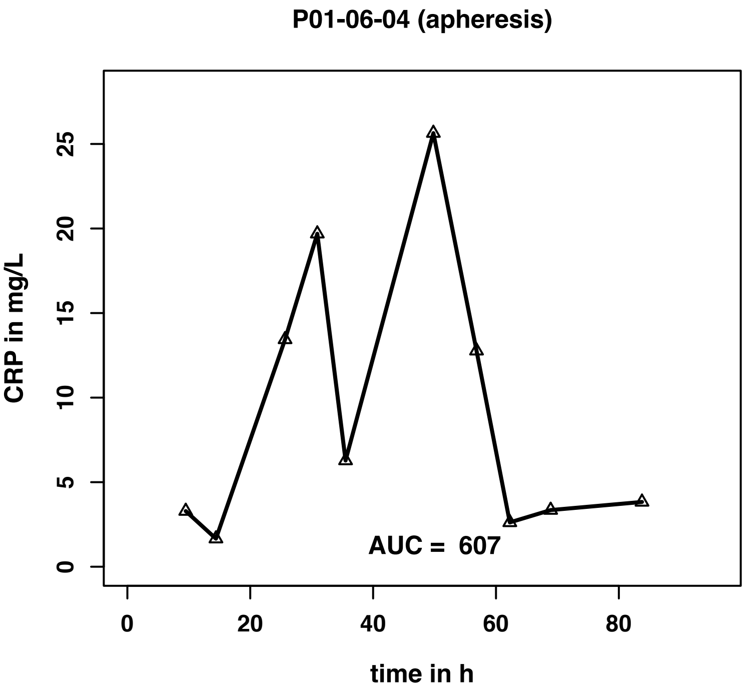

P01-06-04 (apheresis)
CRP in mg/L
time in h
AUC = 607
0
5
10
15
20
25
0
20
40
60
80
